# Supplementary material for: In vitro and in vivo inhibition of the host TRPC4 channel attenuates Zika virus infection
Source: EMBO Mol Med. 2024 Jul 15;16(8):3. doi: 10.1038/s44321-024-00103-4 (PMC11319825; doi:10.1038/s44321-024-00103-4)
Supplement: Supplementary file 1 — Appendix [file 44321_2024_103_MOESM1_ESM.pdf]

Appendix

Appendix Table S1 (p. 1)
Appendix Table S2 (p. 2-3)

Appendix Table S1. mRNA levels of TRPC1, TRPC3, TRPC5 and TRPC6 ZIKV-infected BHK cells

TRPC channels	Relative RNA (mean ± SEM)	
	Mock	ZIKV
TRPC1	1.22 ± 0.30	1.24 ± 0.45
TRPC3	1.02 ± 0.10	0.78 ± 0.03
TRPC5	1.02 ± 0.11	0.76 ± 0.21
TRPC6	1.05 ± 0.16	0.94 ± 0.18

mRNA levels of TRPC1, TRPC3, TRPC5 and TRPC6 were not changed in ZIKV-infected BHK cells compared to controls (n = 3 biological replicates). The mRNA copies of ZIKV were 1.50 ± 0.21×10^3 in ZIKV infected cells at 72 hpi. Data are presented as mean ± SEM.

Appendix Table S2. Statistical methods and the determination of P-values.

Figure number	Exact p-values (from left to right)	statistical test
Figure 1D	**p=0.004	unpaired T-test
Figure 1F left	**p=0.002	unpaired T-test
Figure 1F right	**p=0.003	unpaired T-test
Figure 2B	**p=0.002	One-Way ANOVA followed by the Tukey test
Figure 2C	***p=0.000154	One-Way ANOVA followed by the Tukey test
Figure 2D	***p=0.000000552	unpaired T-test
Figure 3B	*p=0.03; **p=0.005; *p=0.01	One-Way ANOVA on ranks followed Dunnett's test
Figure 3C	***p=0.0001	One-Way ANOVA followed by Student-Newman-Keuls Method
Figure 3D	***p=0.00005; *p=0.03	One-Way ANOVA followed by Student-Newman-Keuls Method
Figure 3F	***p=0.0003	One-Way ANOVA followed by Student-Newman-Keuls Method
Figure 3G	****p=0.00007, 0.00001, 0.00004, 0.00003, 0.0003	S One-Way ANOVA followed by Student-Newman-Keuls Method
Figure 4A	***p=0.00012; **p=0.002; ***p=0.0011	One-Way ANOVA followed by the Dunnett's test
Figure 4B	***p=0.00045; **p=0.002; ***p=0.0002	One-Way ANOVA followed by the Dunnett's test
Figure 4C	***p=0.0008; ***p=0.0006	One-Way ANOVA followed by the Dunnett's test
Figure 4D	**p=0.004; **p=0.004	One-Way ANOVA followed by the Dunnett's test
Figure 4G	*p=003; **p=0.004	One-Way ANOVA followed by the Dunnett's test
Figure 4H	*p=0.02; ***p=0.001	One-Way ANOVA followed by the Dunnett's test
Figure 4J left	**p=0.002	unpaired T-test
Figure 4J right	**p=0.002	unpaired T-test
Figure 4L left	**p=0.003	unpaired T-test
Figure 4L right	**p=0.003	unpaired T-test
Figure 4M	**p=0.009	unpaired T-test

Figure 5B	*p=0.01<br>****p=0.00006, 0.00003, 0.0009, 0.00004, 0.00007	unpaired T-test
Figure 5D	***p=0.0005; **p=0.004; **p=0.004; *p=0.02	One-Way ANOVA followed by the Dunnett's test
Figure 5E	*p=0.04	unpaired T-test
Figure 6B	*p=0.02; *p=0.04	One-Way ANOVA test followed by the Dunnett's test
Figure 6C	*p=0.04; *p=0.02	One-Way ANOVA test followed by the Dunnett's test
Figure 6D	*p=0.02; *p=0.01	One-Way ANOVA test followed by the Dunnett's test
Figure 7C	***p=0.0006; ***p=0.0005	One-Way ANOVA test followed by the Dunnett's test
Figure 7F	**p=0.003; **p=0.001; ***p=0.00004; *p=0.02; **p=0.002; ***p=0.00001; *p=0.03	One-Way ANOVA test followed by the Dunnett's test
Figure EV1D	**p=0.008; **p=0.002	unpaired T-test
Figure EV2F	*p=0.03; *p=0.06	One-Way ANOVA test followed by the Dunnett's test
Figure EV2G	*p=0.02; *p=0.04	MANOVA with Bonferroni correction followed by the Dunnett's test
Figure EV3B	**p=0.007; **p=0.005	unpaired T-test
Figure EV3C	**p=0.007; **p=0.005	unpaired T-test
Figure EV3E	**p=0.003	unpaired T-test
