# Supplementary figures and images for: In vitro and in vivo inhibition of the host TRPC4 channel attenuates Zika virus infection
Source: EMBO Mol Med. 2024 Jul 15;16(8):3. doi: 10.1038/s44321-024-00103-4 (PMC11319825; doi:10.1038/s44321-024-00103-4)

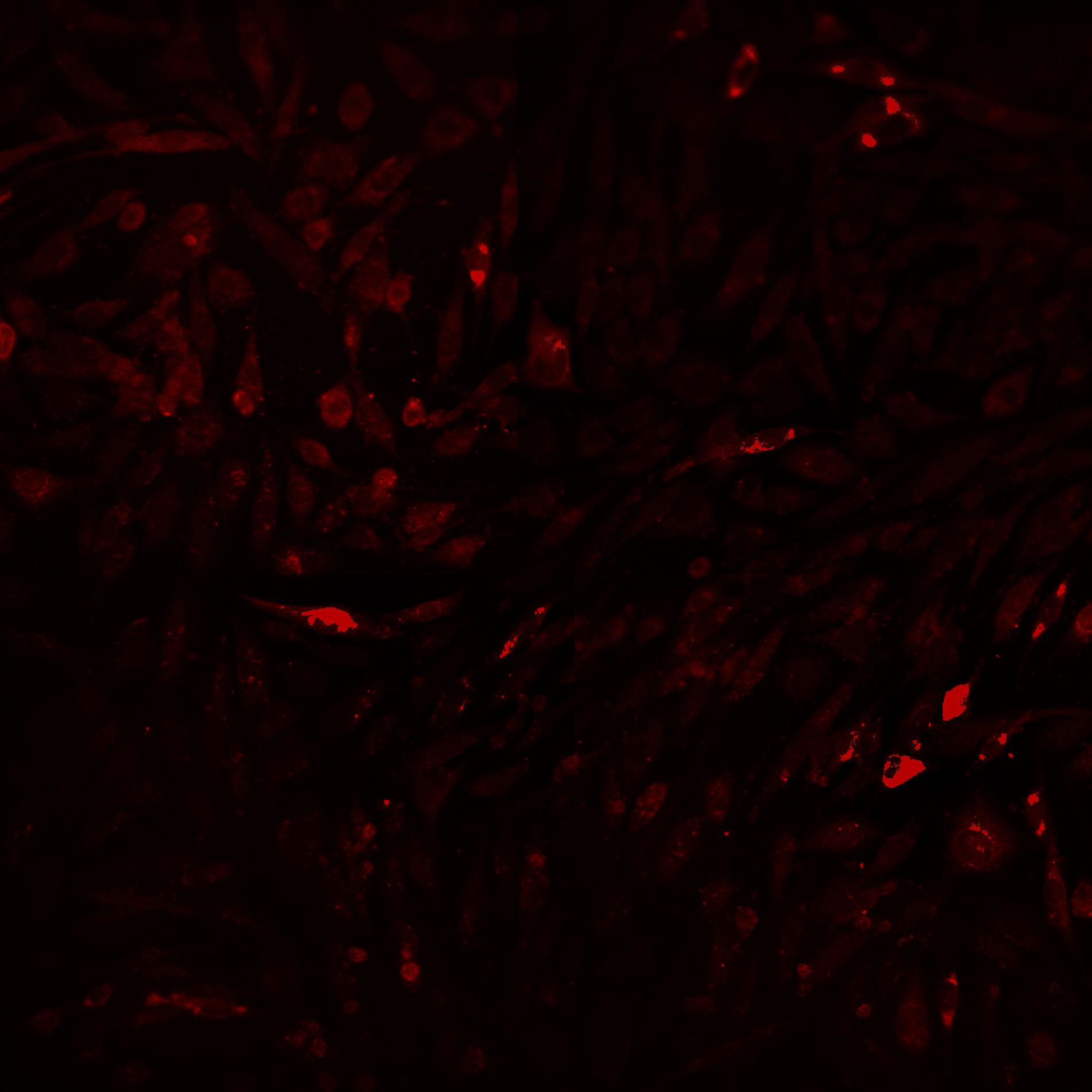

Supplement: Supplementary file 3 — Source data Fig. 1 [file 44321_2024_103_MOESM3_ESM.zip › Figure 1/1A/3 BHK-ctr-1-1000 V EC4 3 R.jpg]

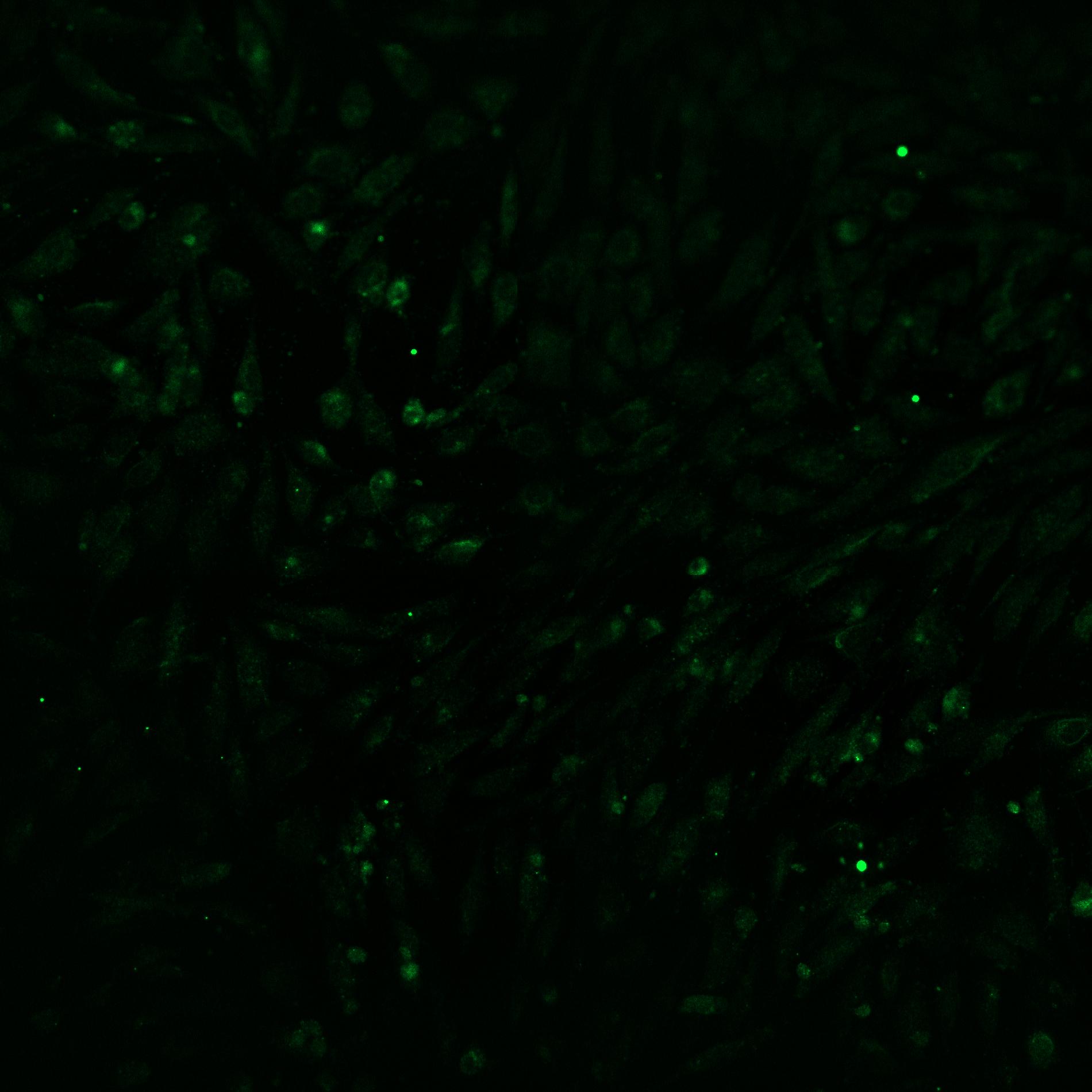

Supplement: Supplementary file 3 — Source data Fig. 1 [file 44321_2024_103_MOESM3_ESM.zip › Figure 1/1A/3 BHK-ctr-1-1000 V EC4 3-G.jpg]

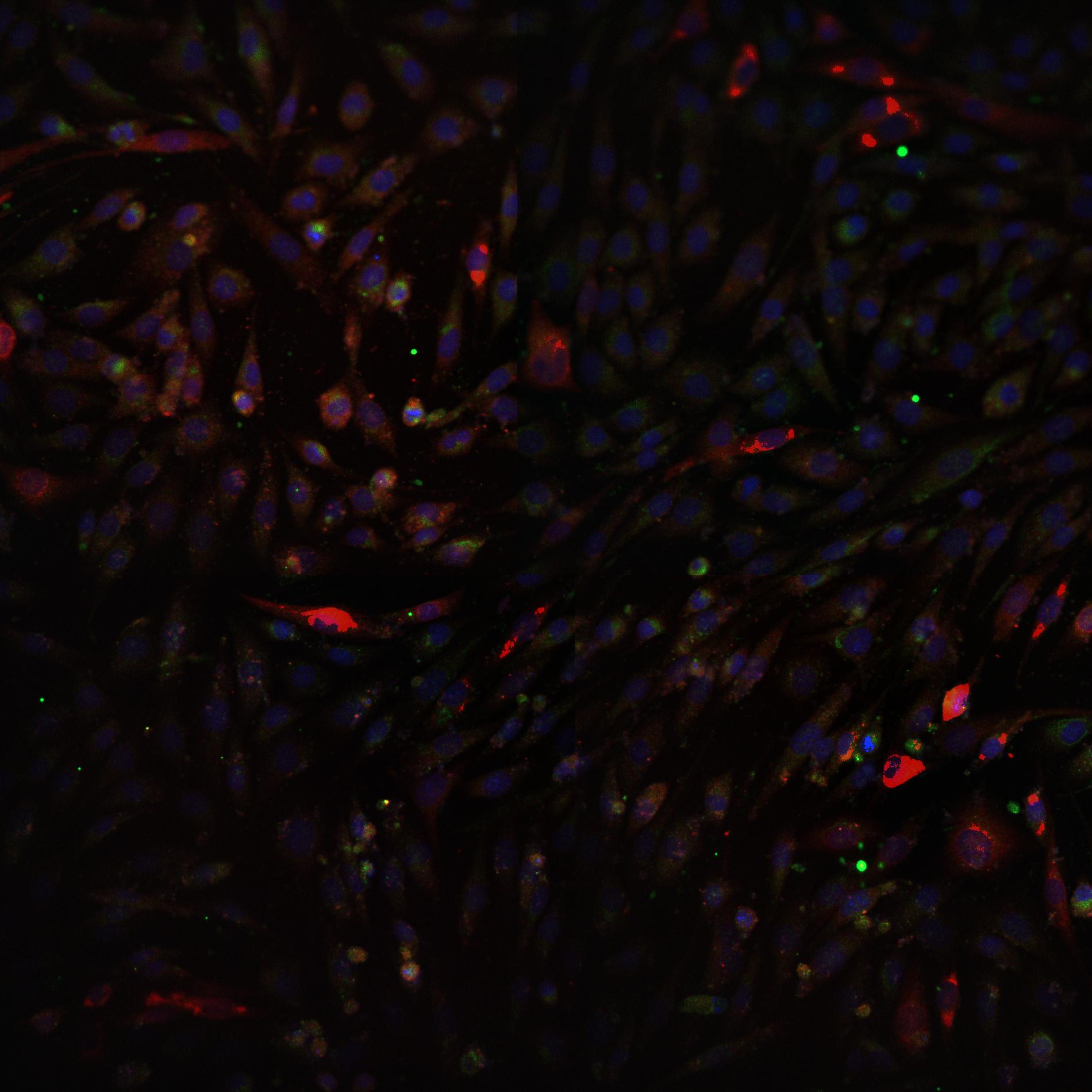

Supplement: Supplementary file 3 — Source data Fig. 1 [file 44321_2024_103_MOESM3_ESM.zip › Figure 1/1A/3 BHK-ctr-1-1000 V EC4 M.jpg]

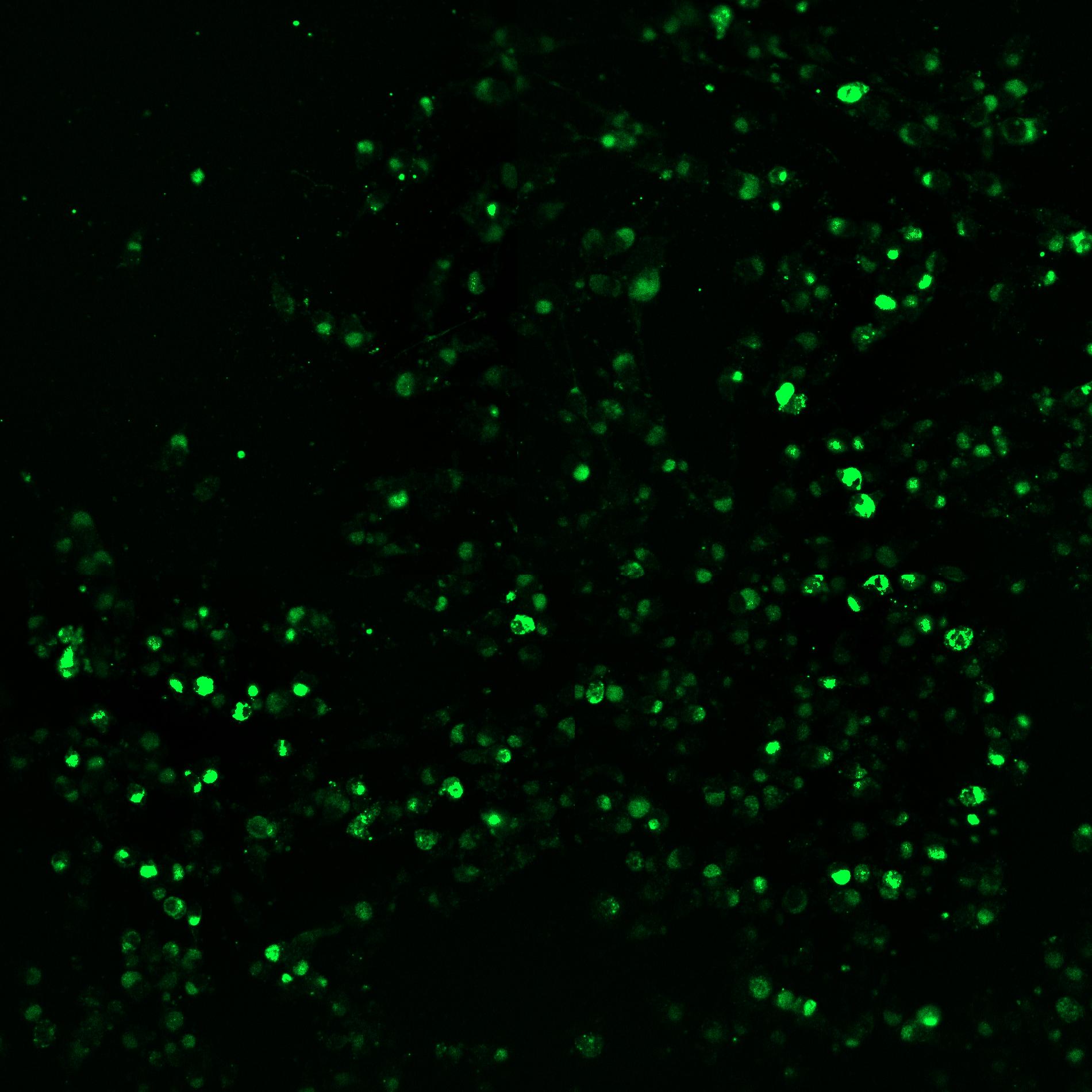

Supplement: Supplementary file 3 — Source data Fig. 1 [file 44321_2024_103_MOESM3_ESM.zip › Figure 1/1A/BHK-ctr -1-100 V EC4 2G.jpg]

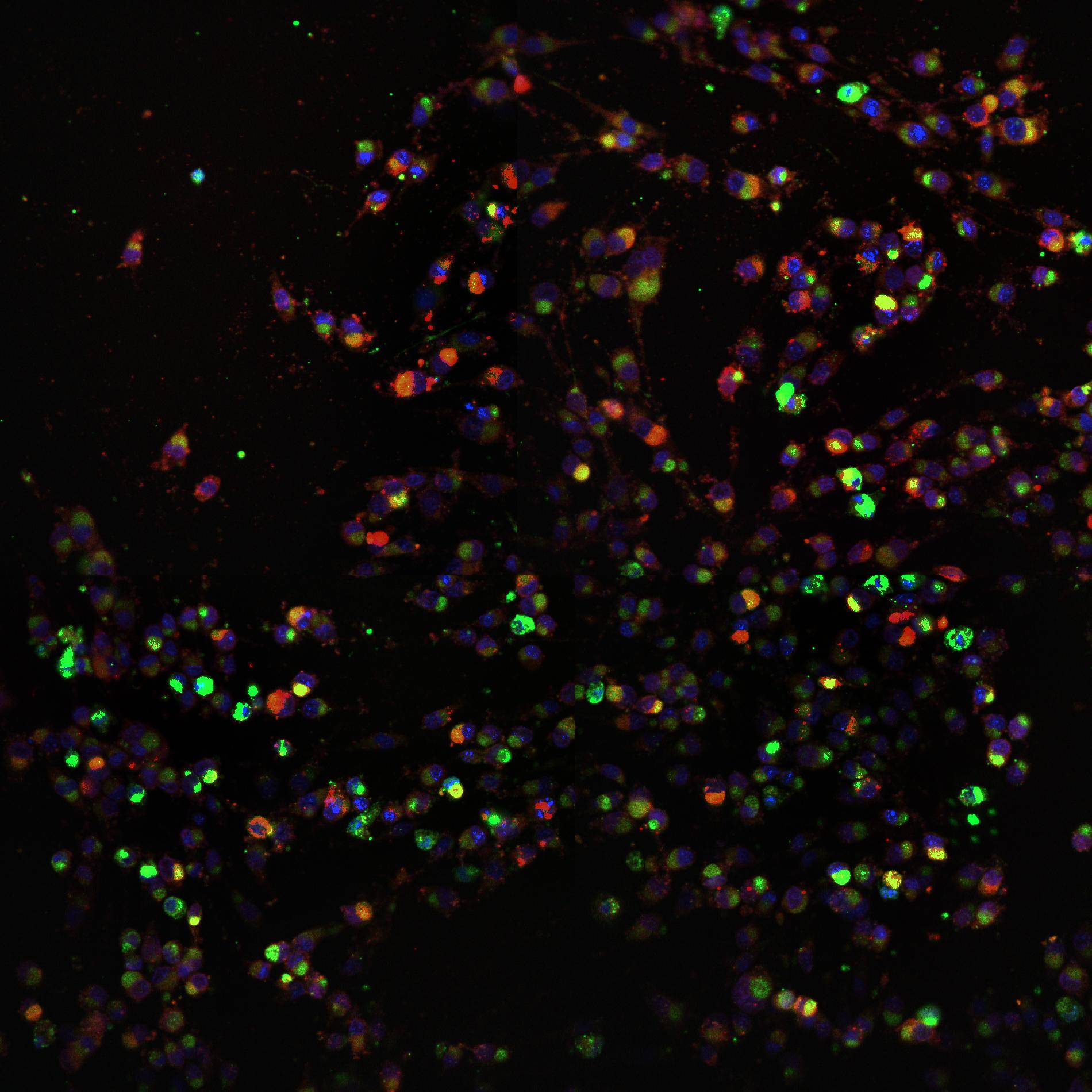

Supplement: Supplementary file 3 — Source data Fig. 1 [file 44321_2024_103_MOESM3_ESM.zip › Figure 1/1A/BHK-ctr -1-100 V EC4 2M.jpg]

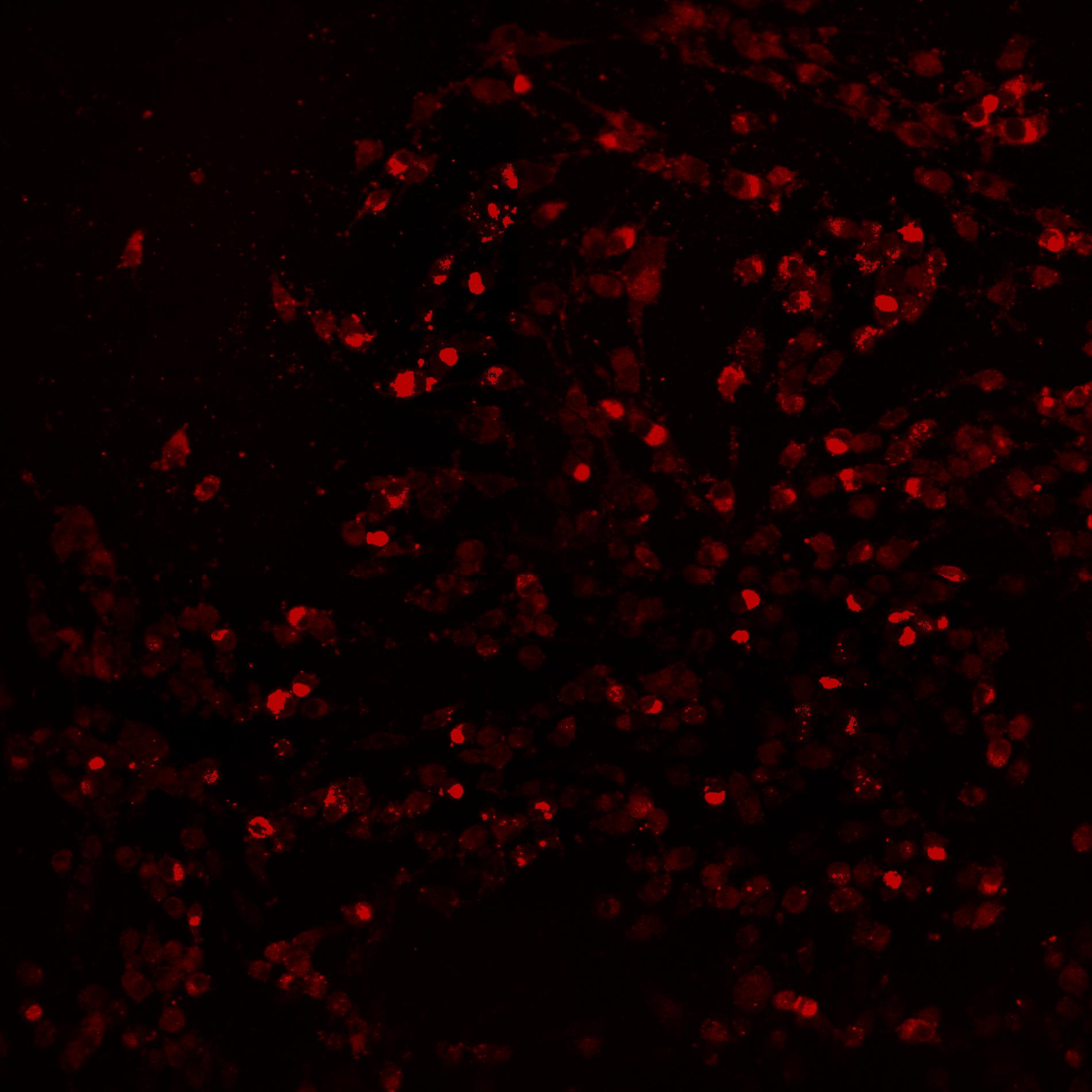

Supplement: Supplementary file 3 — Source data Fig. 1 [file 44321_2024_103_MOESM3_ESM.zip › Figure 1/1A/BHK-ctr -1-100 V EC4 2R.jpg]

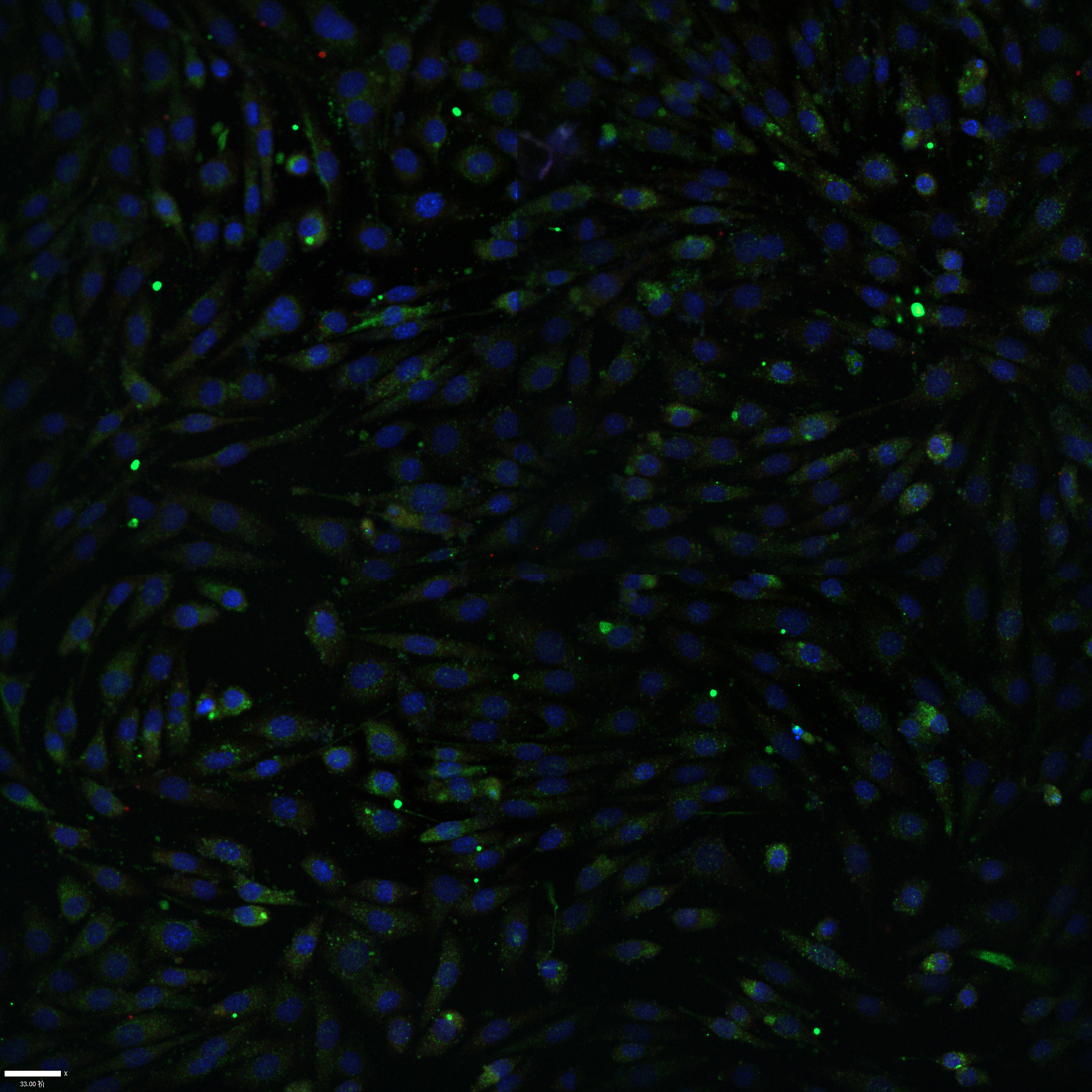

Supplement: Supplementary file 3 — Source data Fig. 1 [file 44321_2024_103_MOESM3_ESM.zip › Figure 1/1A/BHK-ctr-no V EC4 m.tif]

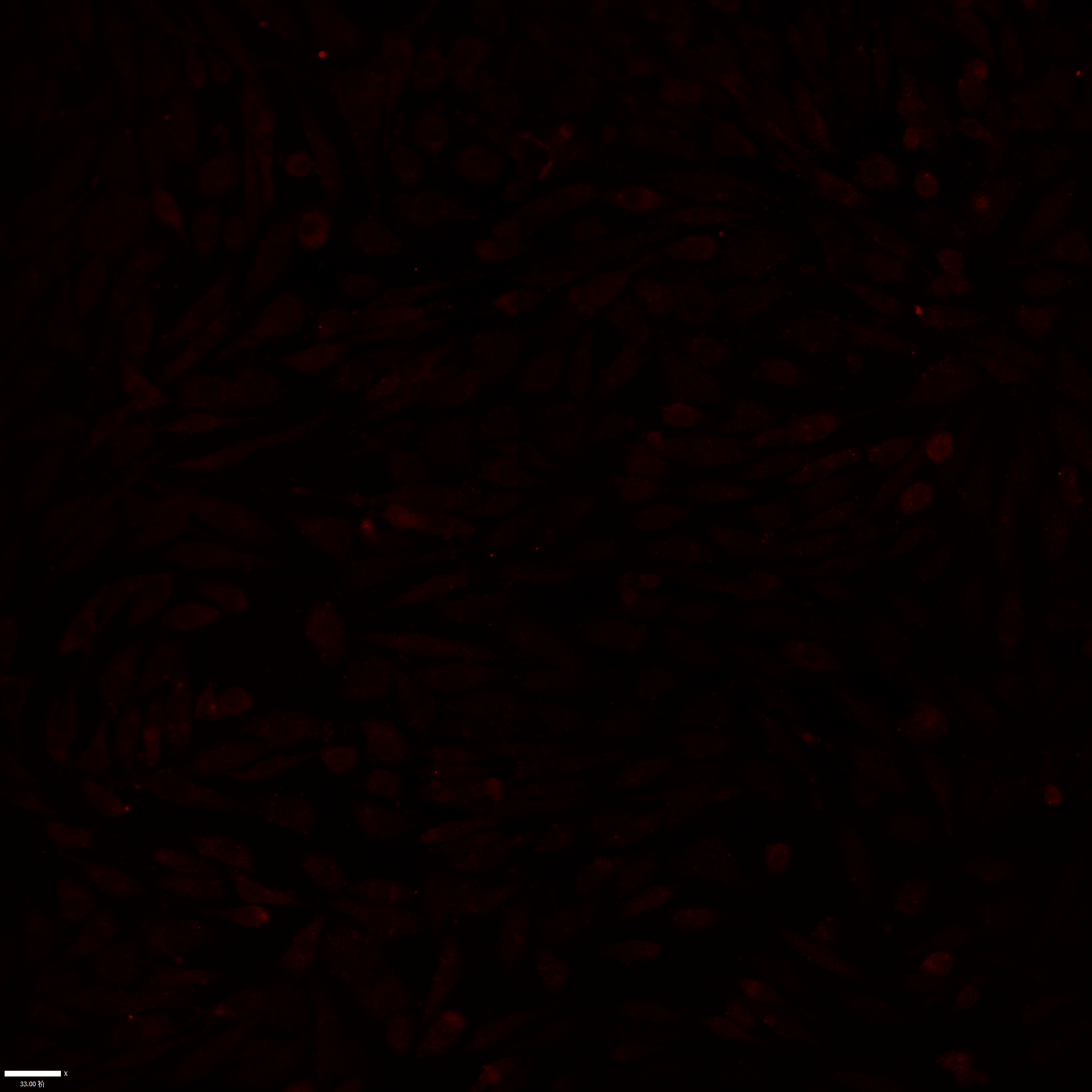

Supplement: Supplementary file 3 — Source data Fig. 1 [file 44321_2024_103_MOESM3_ESM.zip › Figure 1/1A/BHK-ctr-no V EC4 red.tif]

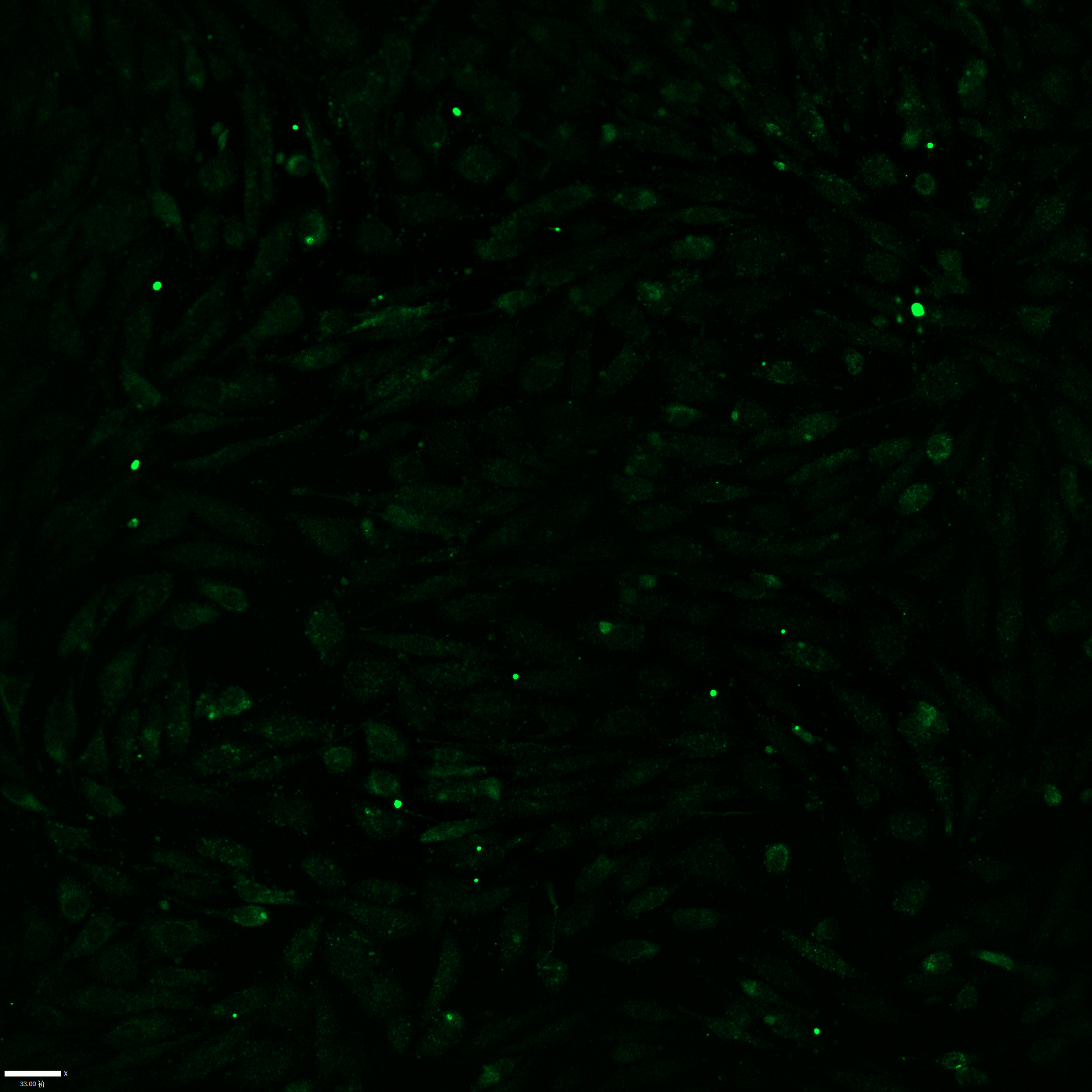

Supplement: Supplementary file 3 — Source data Fig. 1 [file 44321_2024_103_MOESM3_ESM.zip › Figure 1/1A/BHK-ctr-no V EC4.tif]

## Slide 1
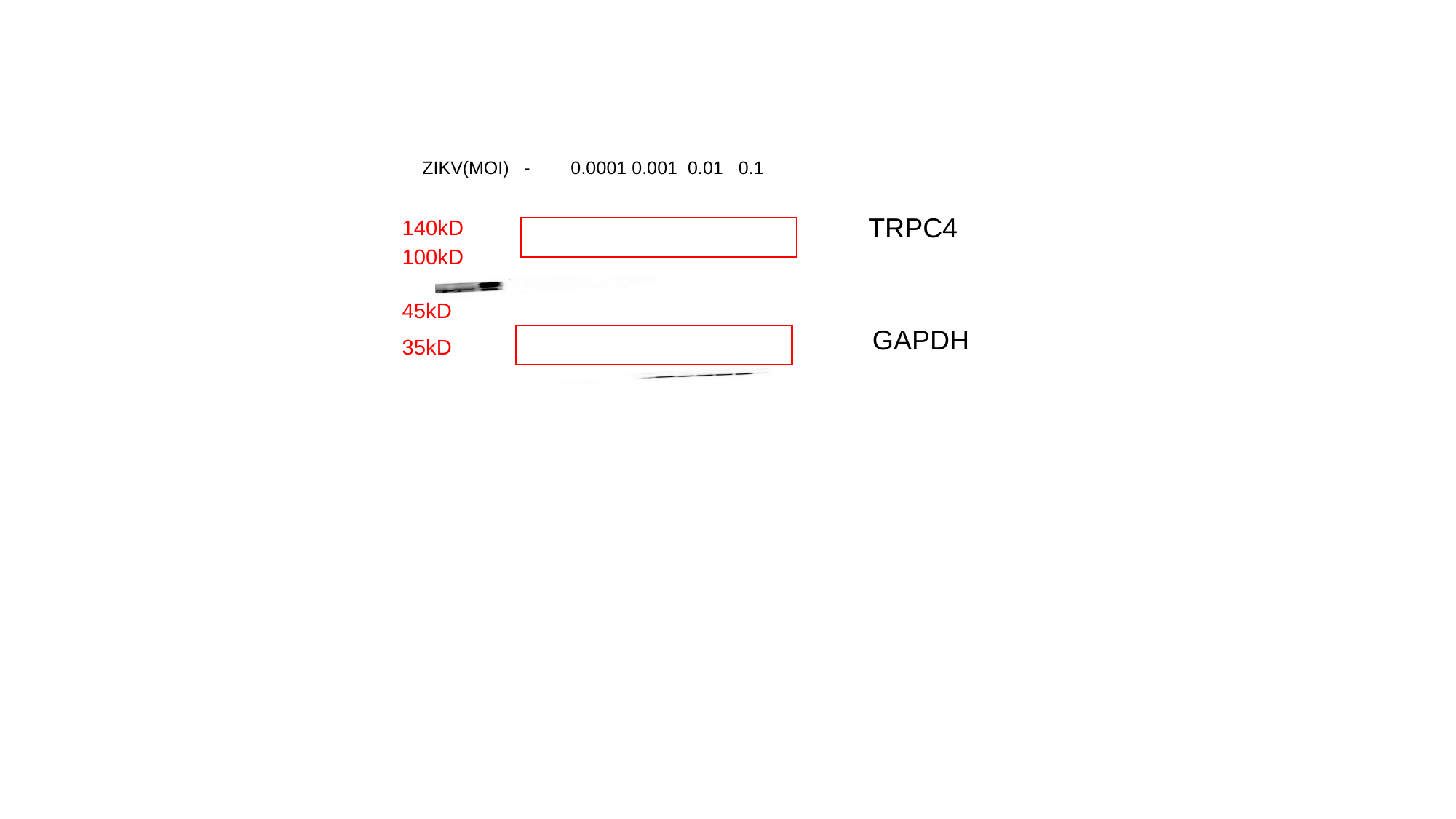

ZIKV(MOI) - 0.0001 0.001 0.01 0.1
TRPC4
140kD
100kD
45kD
GAPDH
35kD

Supplement: Supplementary file 3 — Source data Fig. 1 [file 44321_2024_103_MOESM3_ESM.zip › Figure 1/1C/WB bands.pptx]

## Slide 1
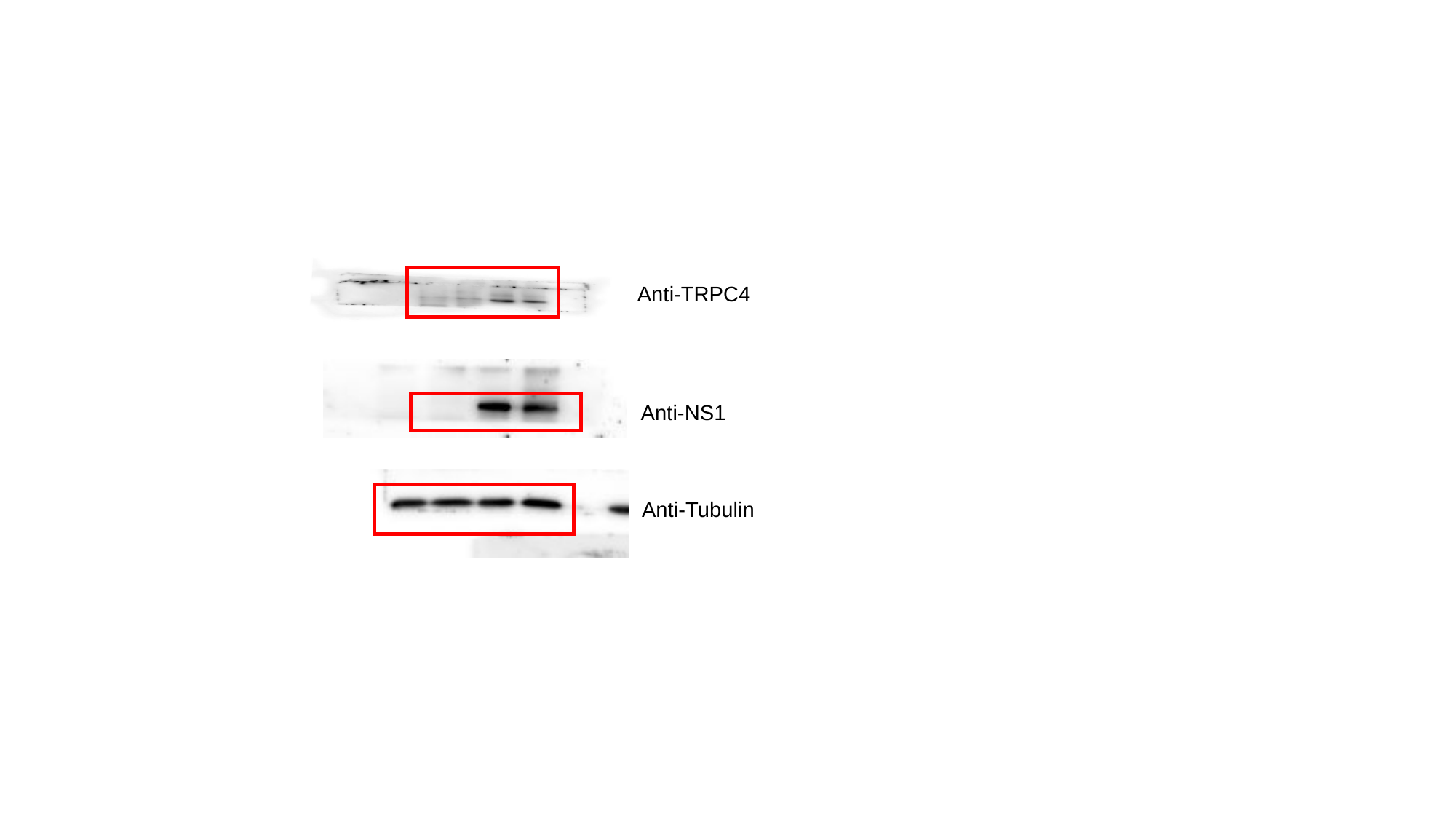

Anti-TRPC4
Anti-NS1
Anti-Tubulin

Supplement: Supplementary file 3 — Source data Fig. 1 [file 44321_2024_103_MOESM3_ESM.zip › Figure 1/1D/WB bands.pptx]

## Slide 1
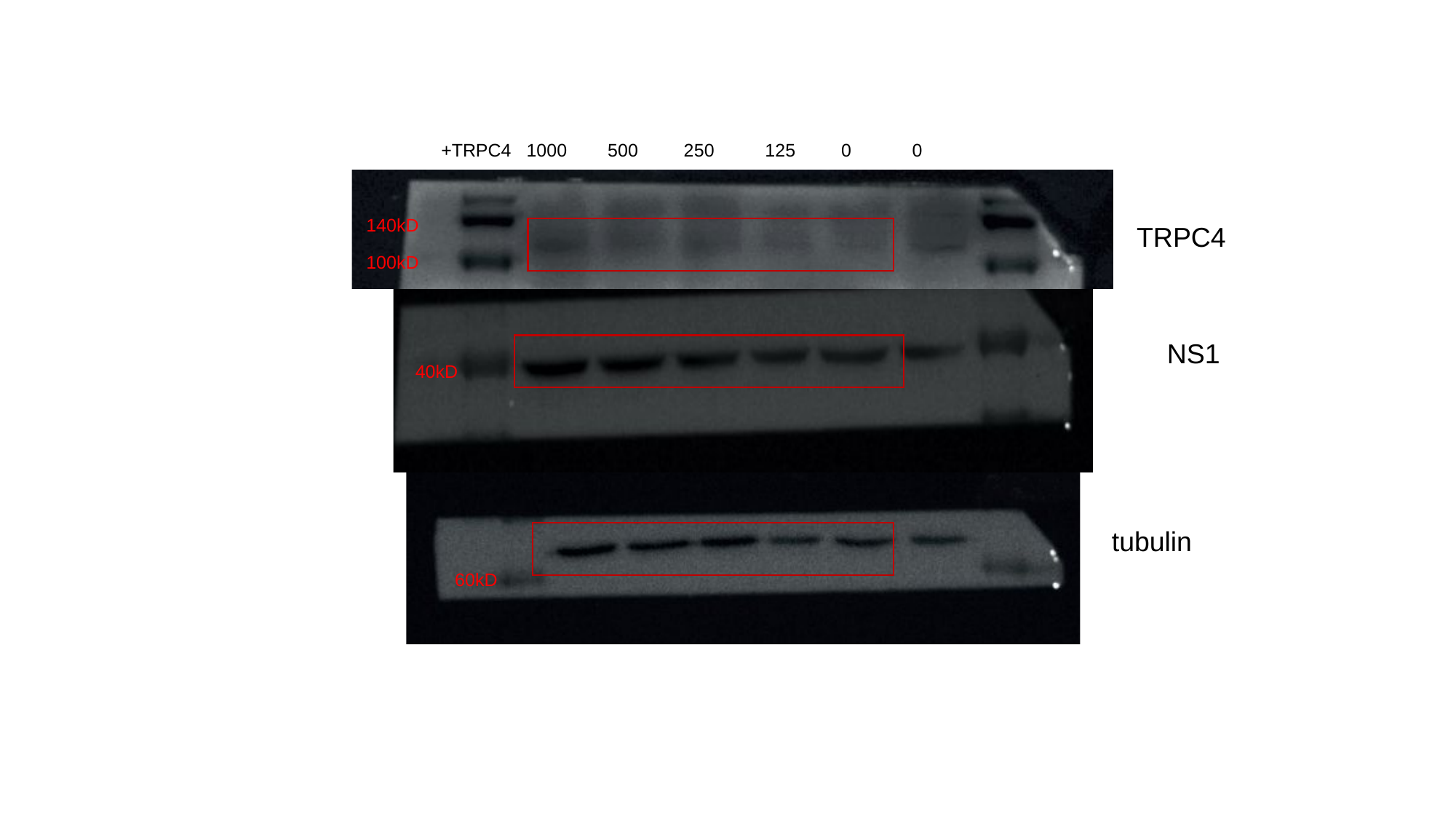

+TRPC4 1000 500 250 125 0 0
140kD
TRPC4
100kD
NS1
40kD
tubulin
60kD

Supplement: Supplementary file 3 — Source data Fig. 1 [file 44321_2024_103_MOESM3_ESM.zip › Figure 1/1E/WB bands.pptx]

## Slide 1
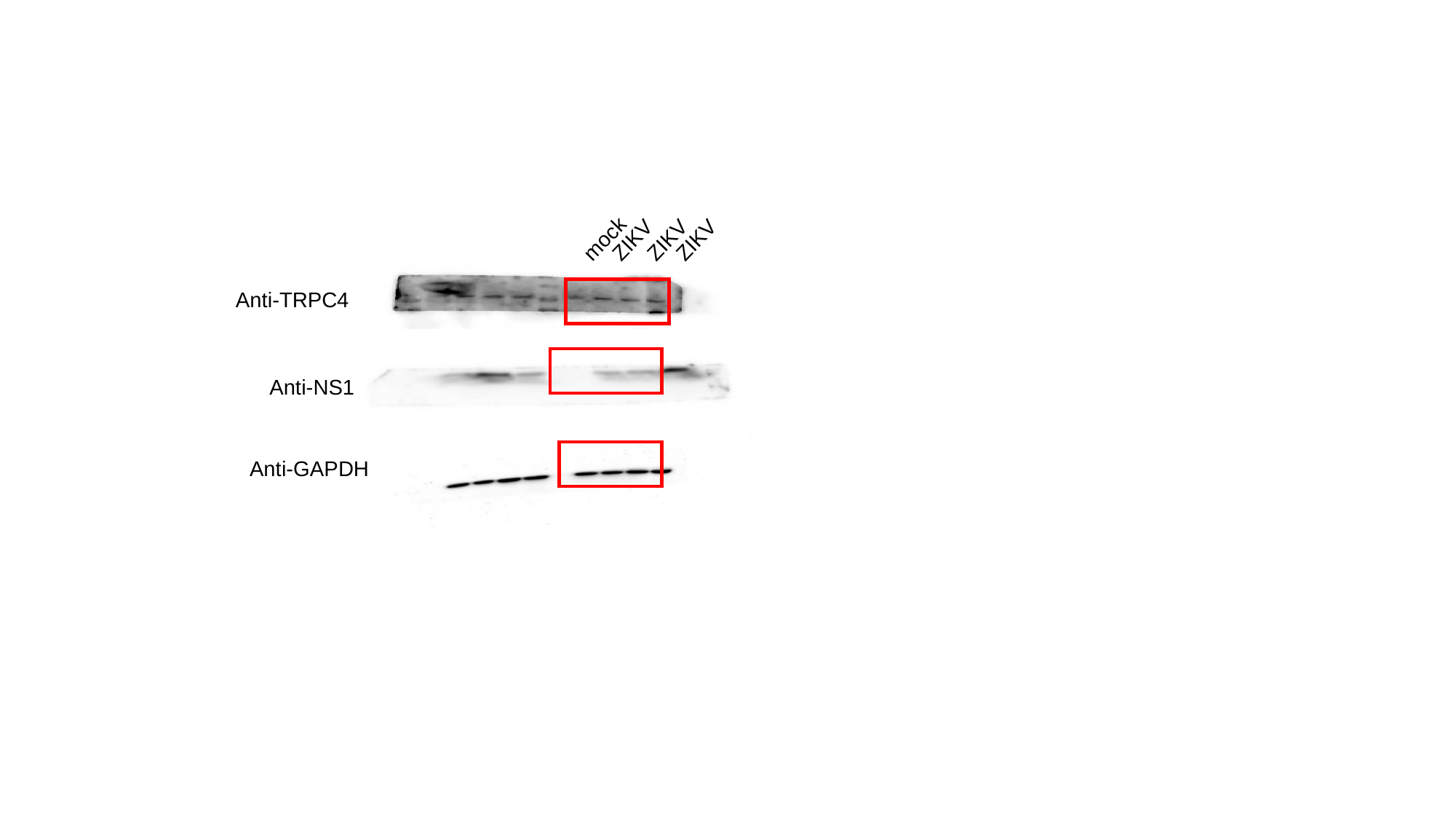

mock
ZIKV
ZIKV
ZIKV
Anti-TRPC4
Anti-NS1
Anti-GAPDH

Supplement: Supplementary file 3 — Source data Fig. 1 [file 44321_2024_103_MOESM3_ESM.zip › Figure 1/1H/WB bands.pptx]

## Slide 1
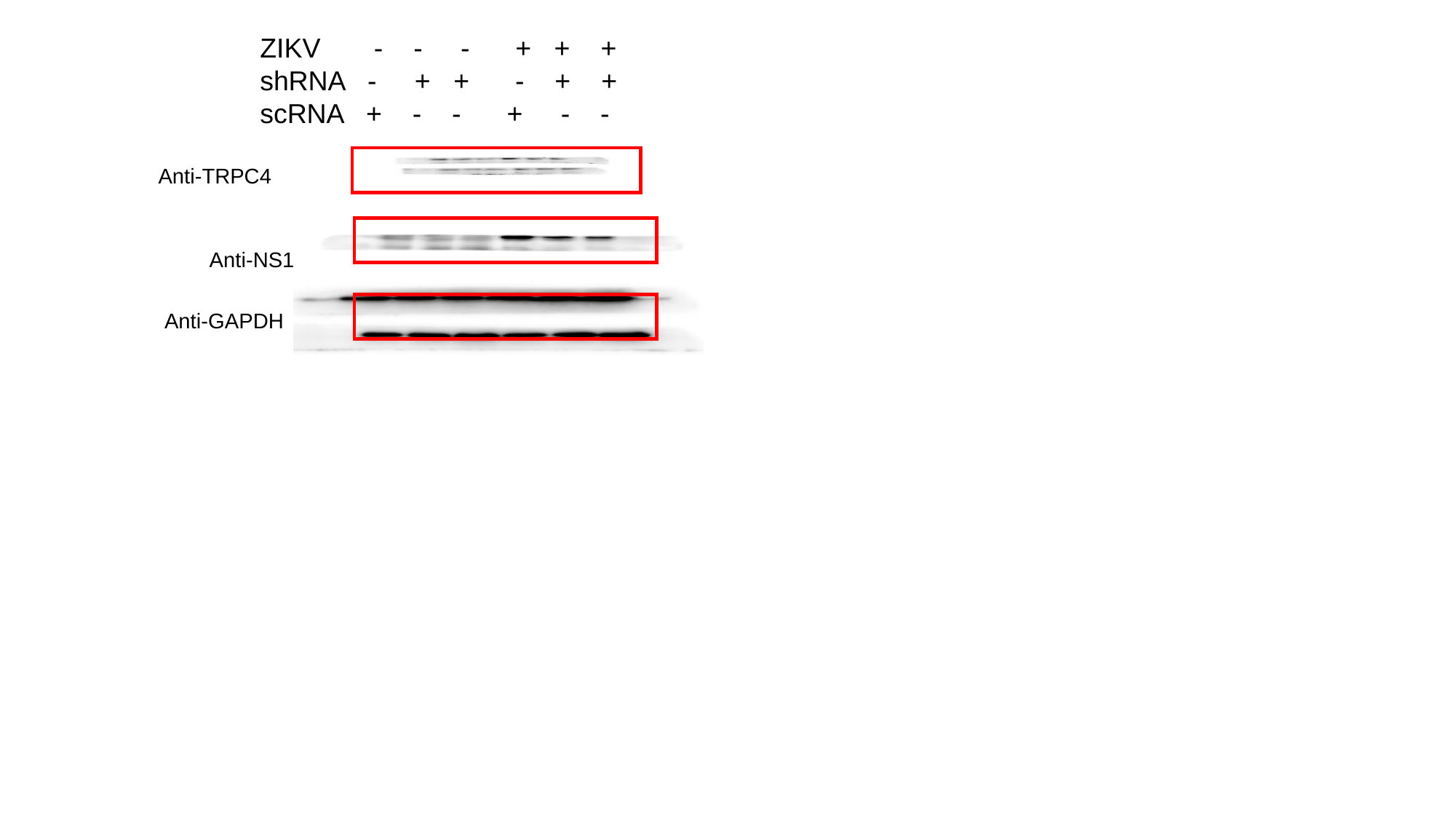

ZIKV - - - + + +
shRNA - + + - + +
scRNA + - - + - -
Anti-TRPC4
Anti-NS1
Anti-GAPDH

Supplement: Supplementary file 4 — Source data Fig. 2 [file 44321_2024_103_MOESM4_ESM.zip › Figure 2/2A/WB bands.pptx]

## Slide 1
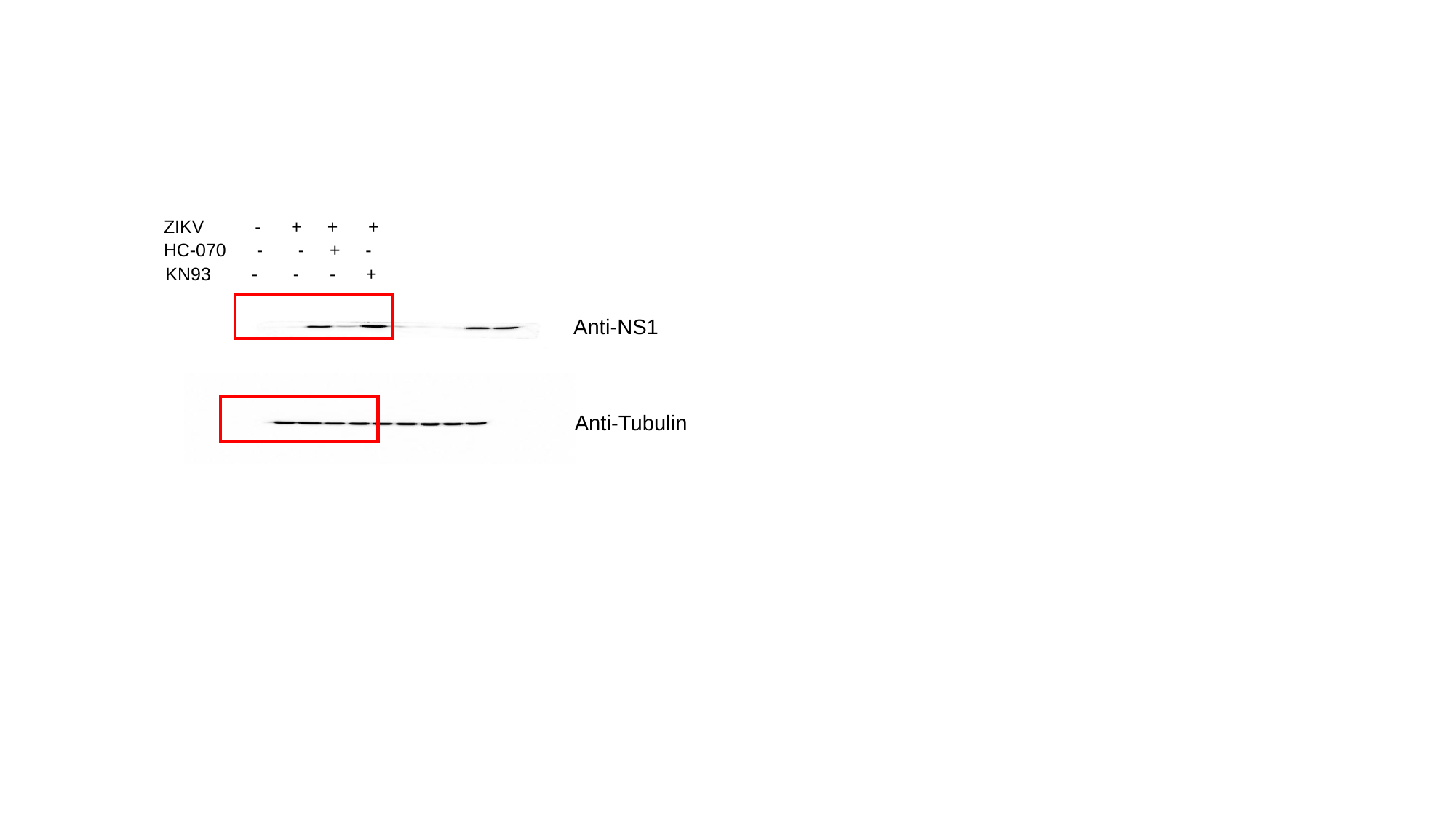

ZIKV - + + +
HC-070 - - + -
KN93 - - - +
Anti-NS1
Anti-Tubulin

Supplement: Supplementary file 5 — Source data Fig. 3 [file 44321_2024_103_MOESM5_ESM.zip › Figure 3/3C/WB bands.pptx]

## Slide 1
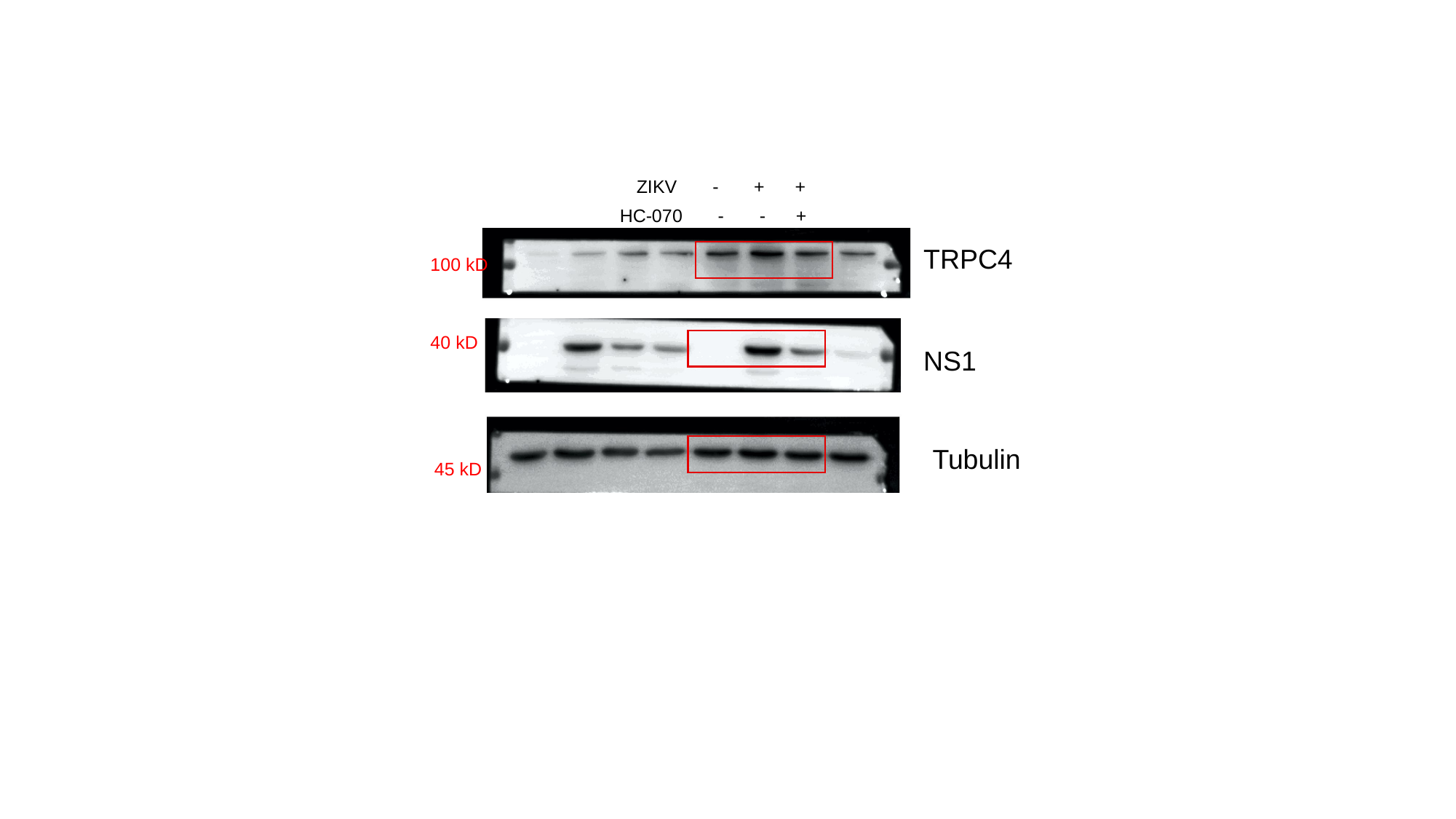

ZIKV - + +
HC-070 - - +
TRPC4
100 kD
40 kD
NS1
Tubulin
45 kD

Supplement: Supplementary file 5 — Source data Fig. 3 [file 44321_2024_103_MOESM5_ESM.zip › Figure 3/3D/WB bands.pptx]

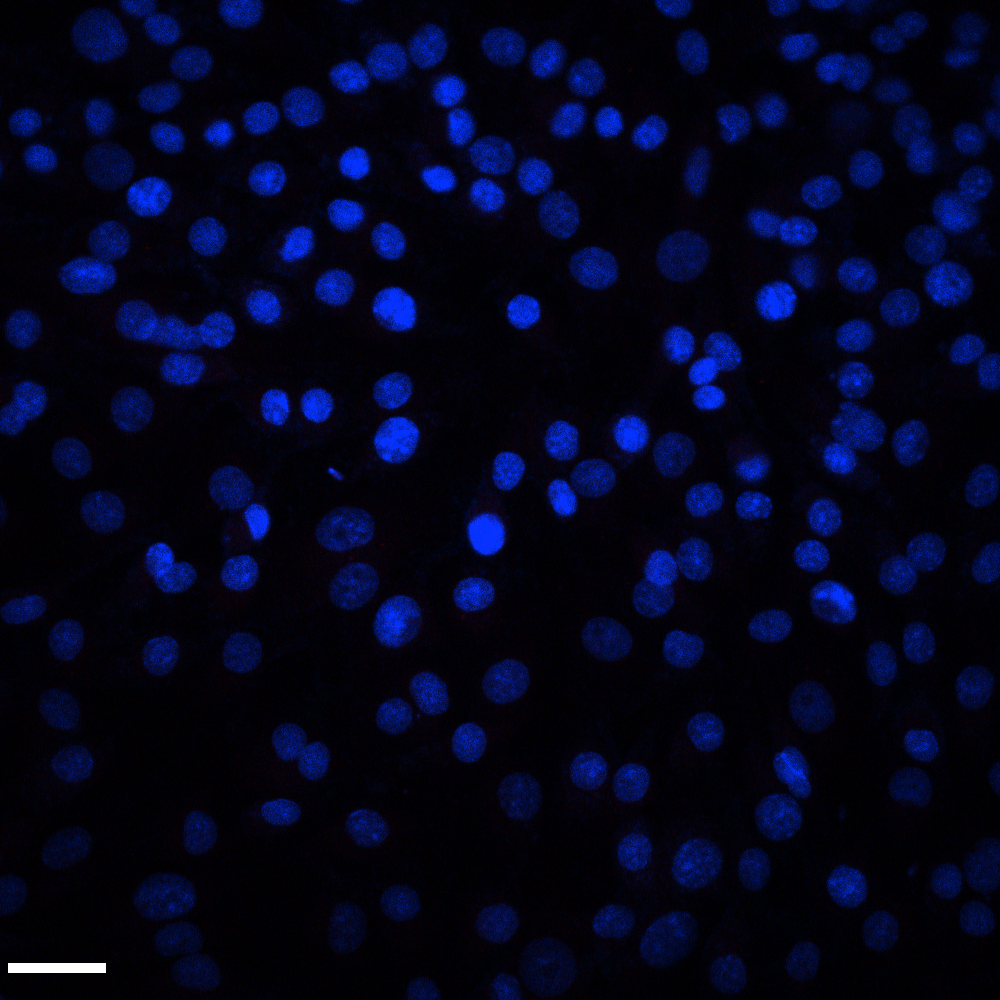

Supplement: Supplementary file 5 — Source data Fig. 3 [file 44321_2024_103_MOESM5_ESM.zip › Figure 3/3E/BHK-ZIKV 7DAY HC070 3 MERGED.tif]

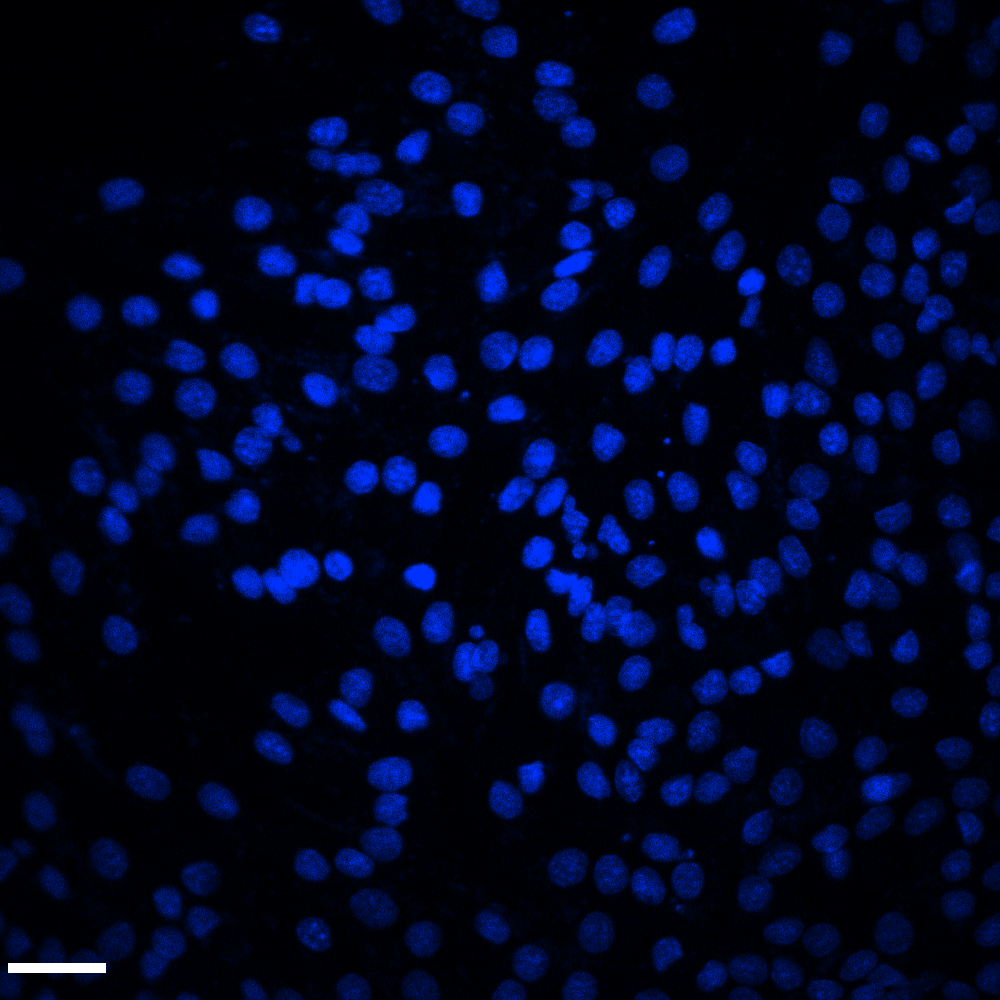

Supplement: Supplementary file 5 — Source data Fig. 3 [file 44321_2024_103_MOESM5_ESM.zip › Figure 3/3E/BHK-ZIKV AC1903 3 DAPI.tif]

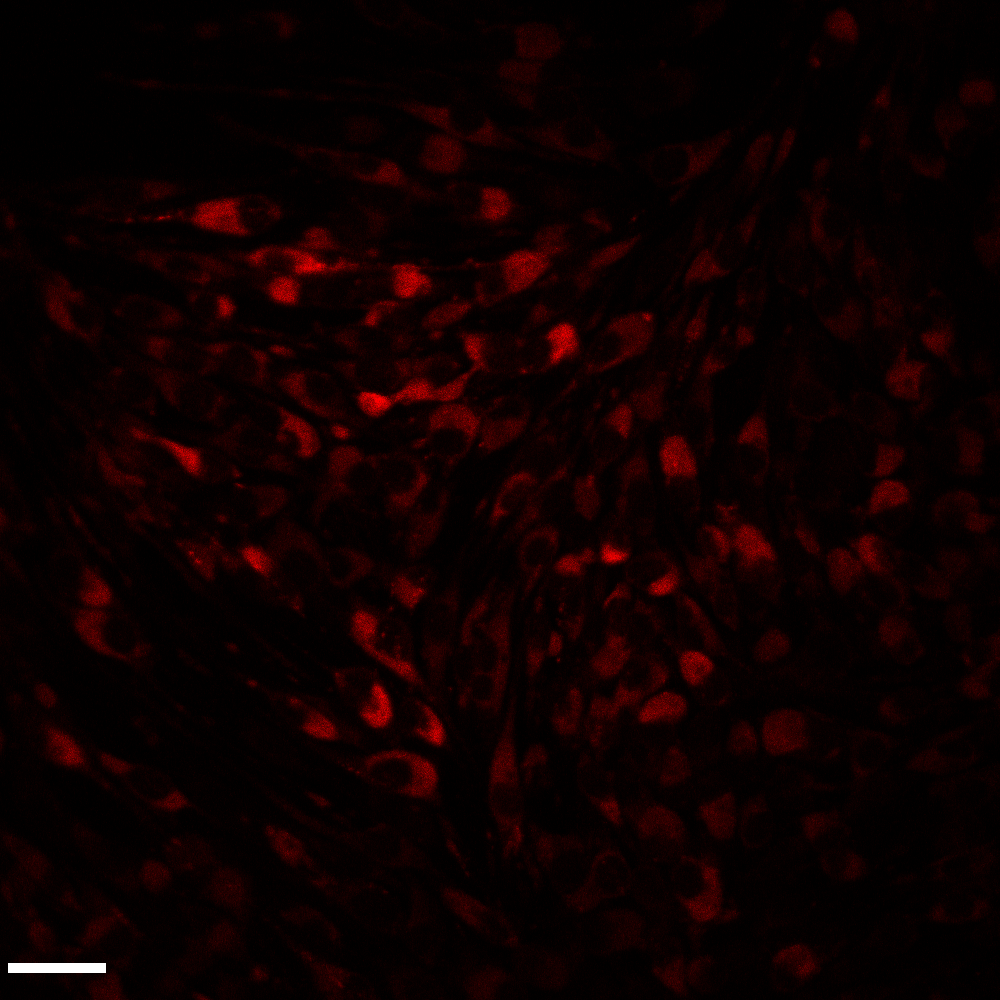

Supplement: Supplementary file 5 — Source data Fig. 3 [file 44321_2024_103_MOESM5_ESM.zip › Figure 3/3E/BHK-ZIKV AC1903 3 Eprotein.tif]

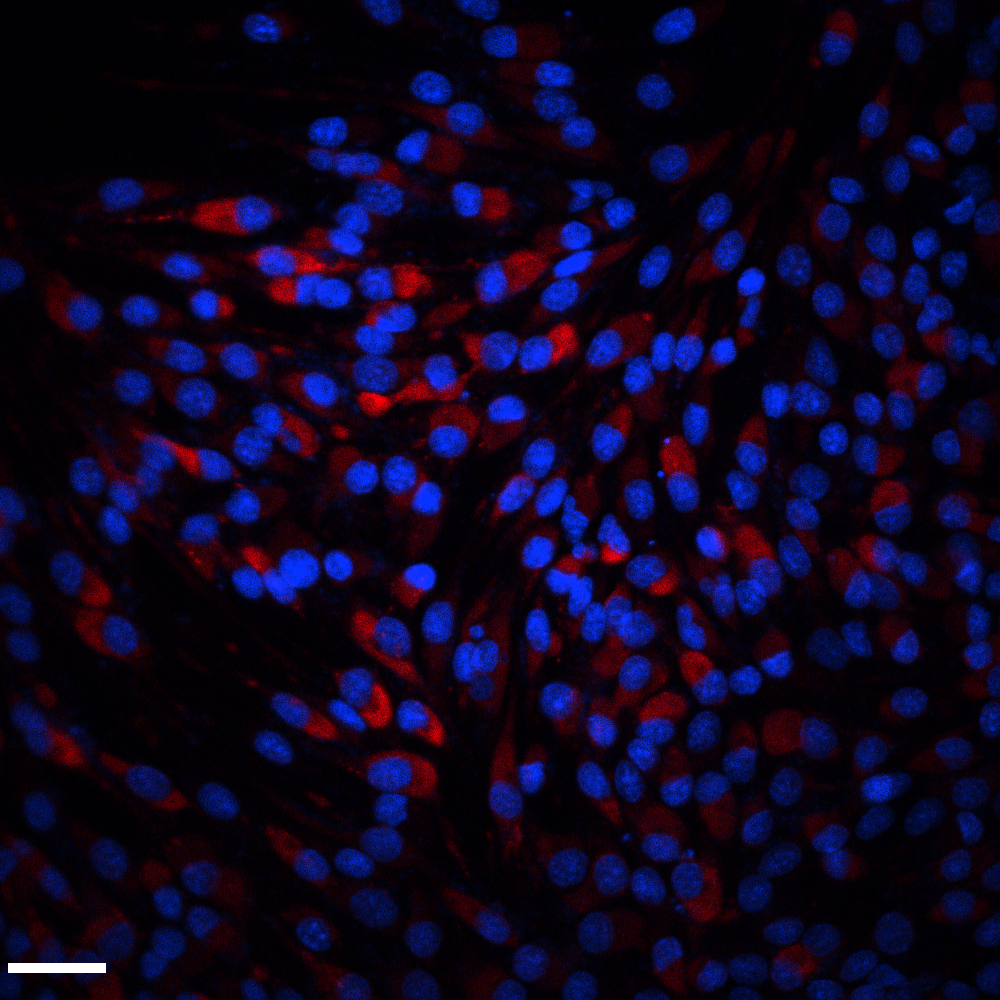

Supplement: Supplementary file 5 — Source data Fig. 3 [file 44321_2024_103_MOESM5_ESM.zip › Figure 3/3E/BHK-ZIKV AC1903 3 MERGED.tif]

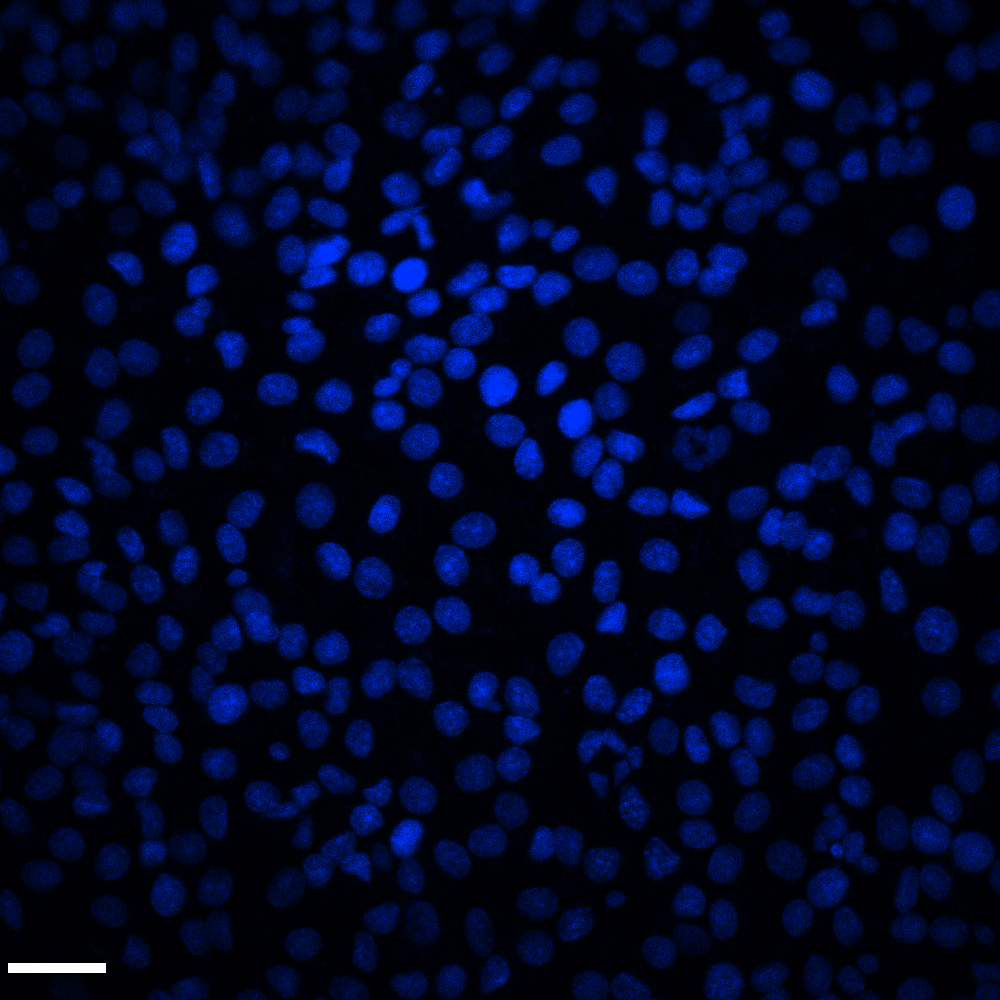

Supplement: Supplementary file 5 — Source data Fig. 3 [file 44321_2024_103_MOESM5_ESM.zip › Figure 3/3E/BHK-ZIKV DMSO 5 DAPI.tif]

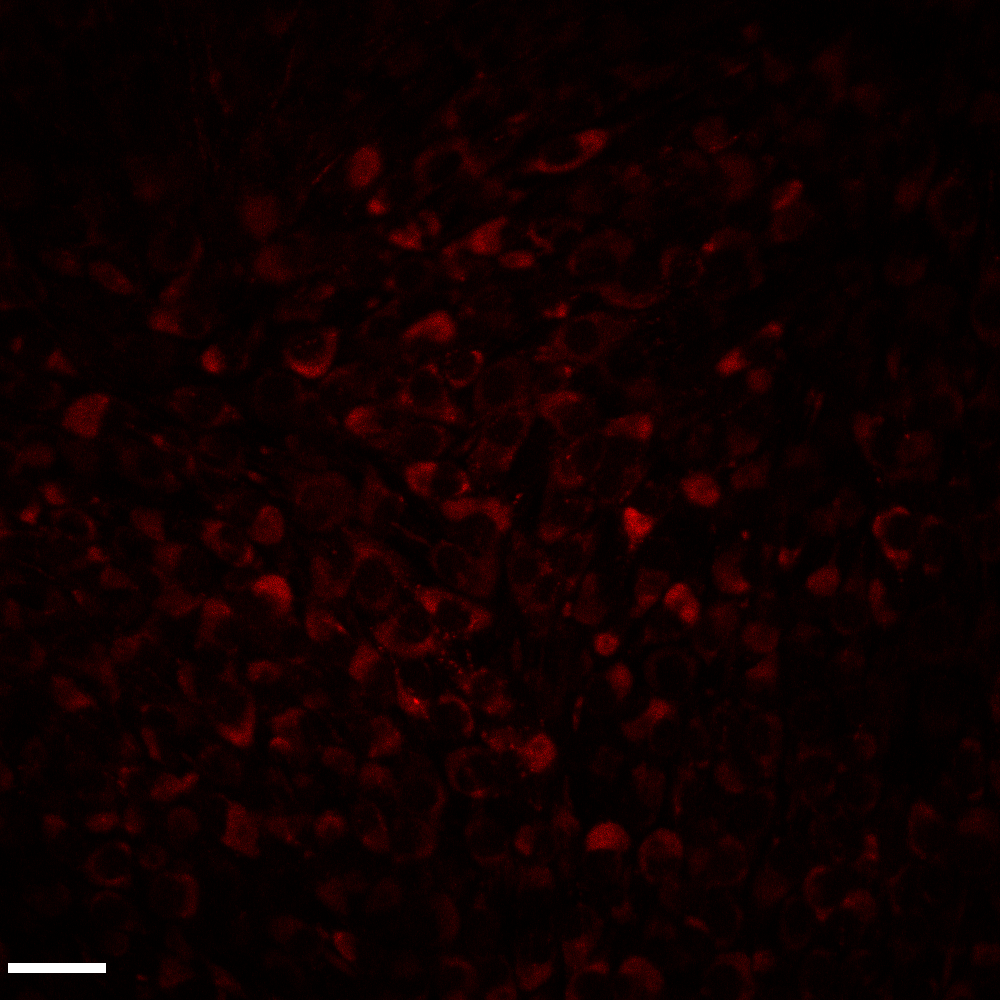

Supplement: Supplementary file 5 — Source data Fig. 3 [file 44321_2024_103_MOESM5_ESM.zip › Figure 3/3E/BHK-ZIKV DMSO 5 Eprotein.tif]

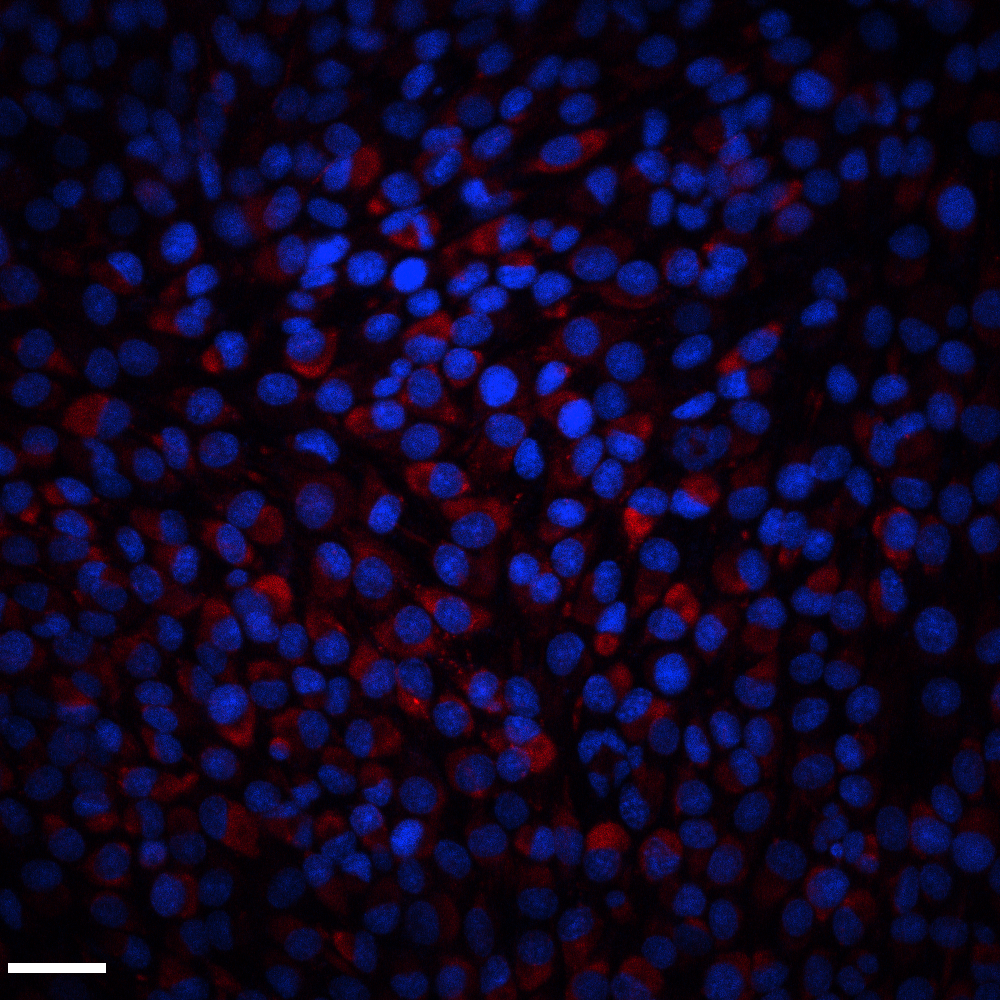

Supplement: Supplementary file 5 — Source data Fig. 3 [file 44321_2024_103_MOESM5_ESM.zip › Figure 3/3E/BHK-ZIKV DMSO 5 MERGED.tif]

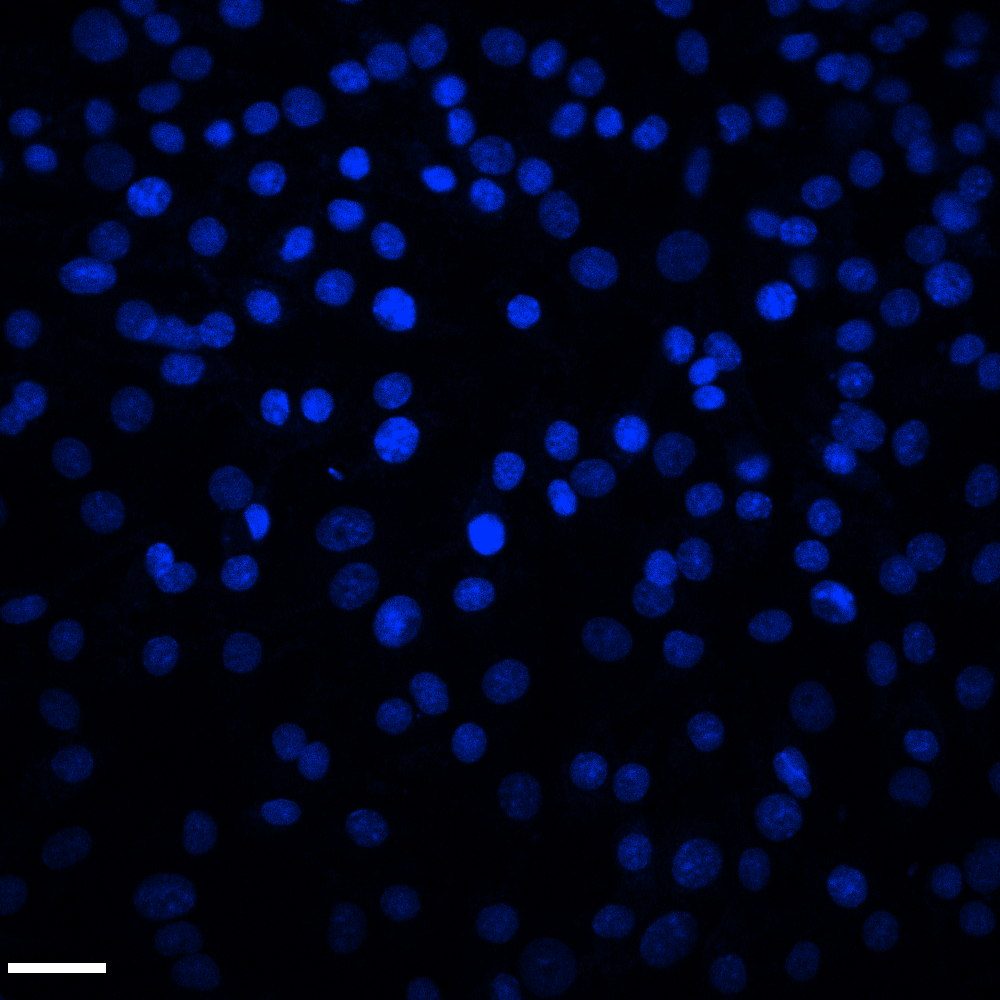

Supplement: Supplementary file 5 — Source data Fig. 3 [file 44321_2024_103_MOESM5_ESM.zip › Figure 3/3E/BHK-ZIKV HC070 3 DAPI.tif]

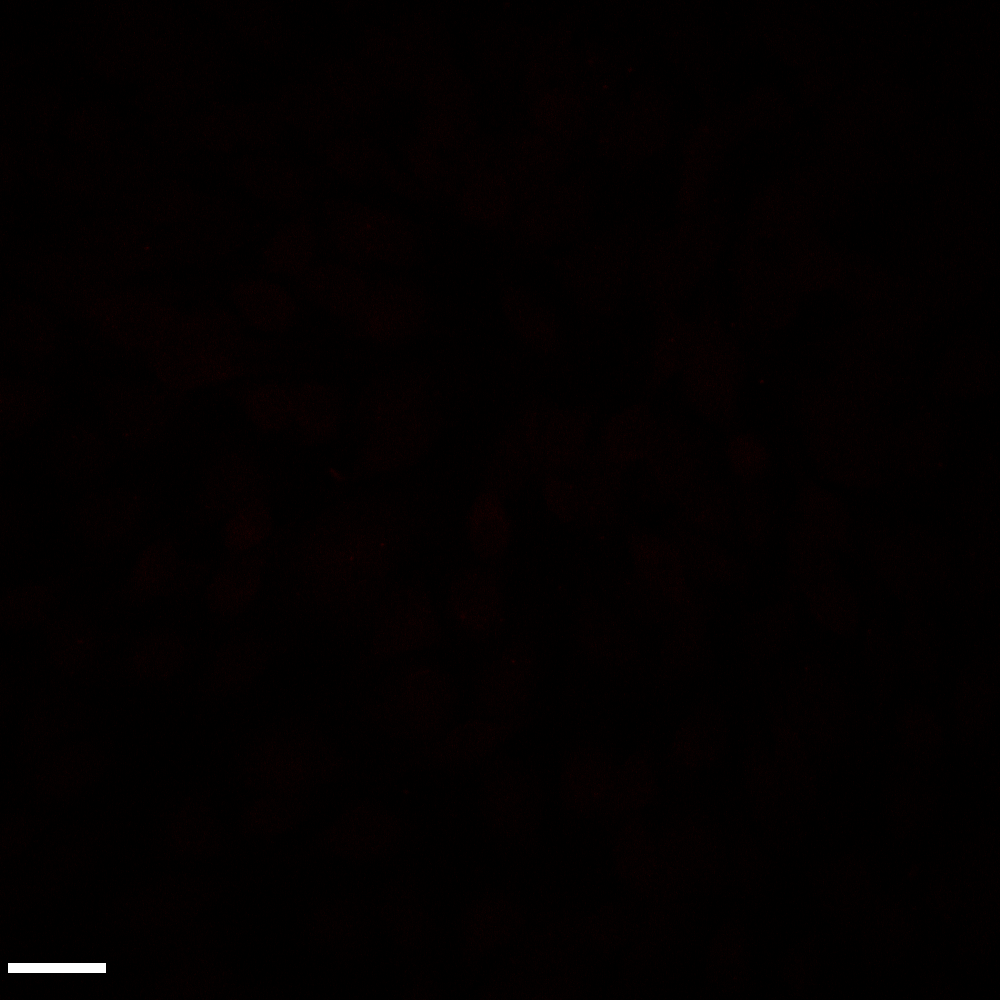

Supplement: Supplementary file 5 — Source data Fig. 3 [file 44321_2024_103_MOESM5_ESM.zip › Figure 3/3E/BHK-ZIKV HC070 3Eprotein.tif]

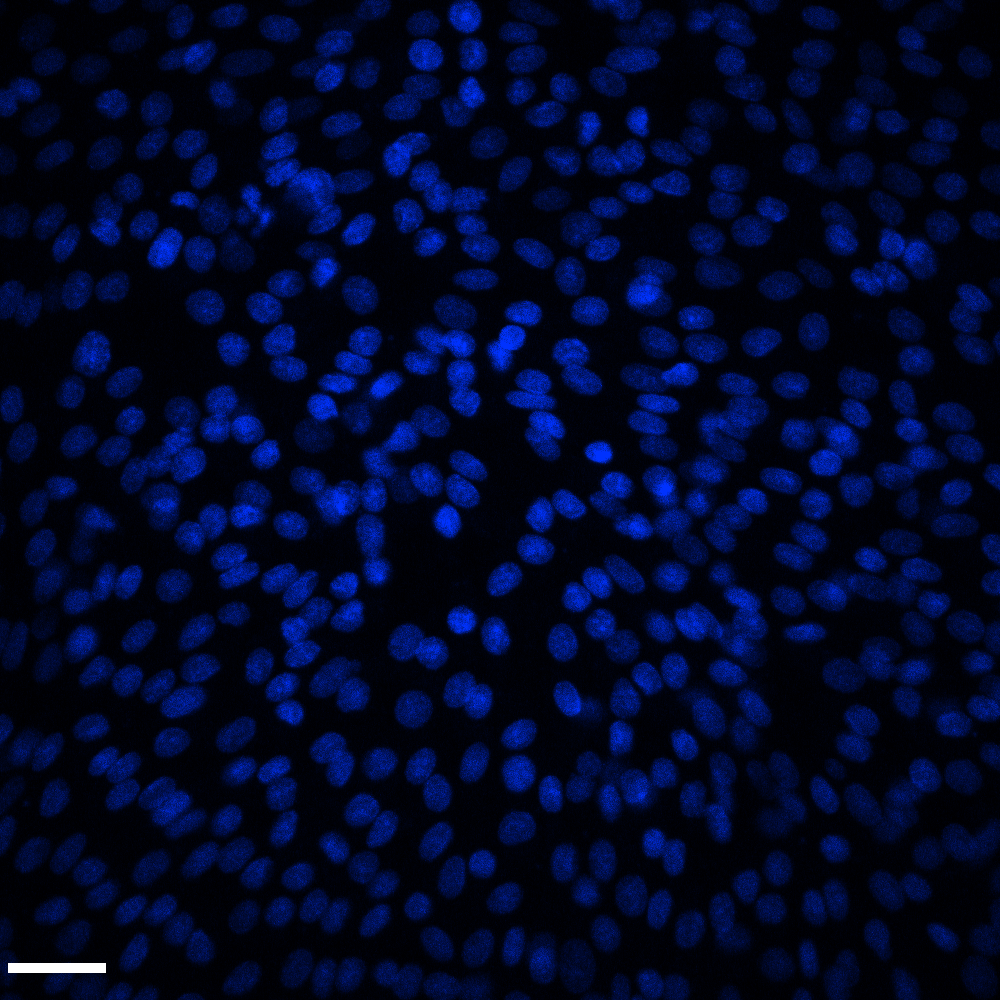

Supplement: Supplementary file 5 — Source data Fig. 3 [file 44321_2024_103_MOESM5_ESM.zip › Figure 3/3E/BHK-ZIKV mock DAPI.tif]

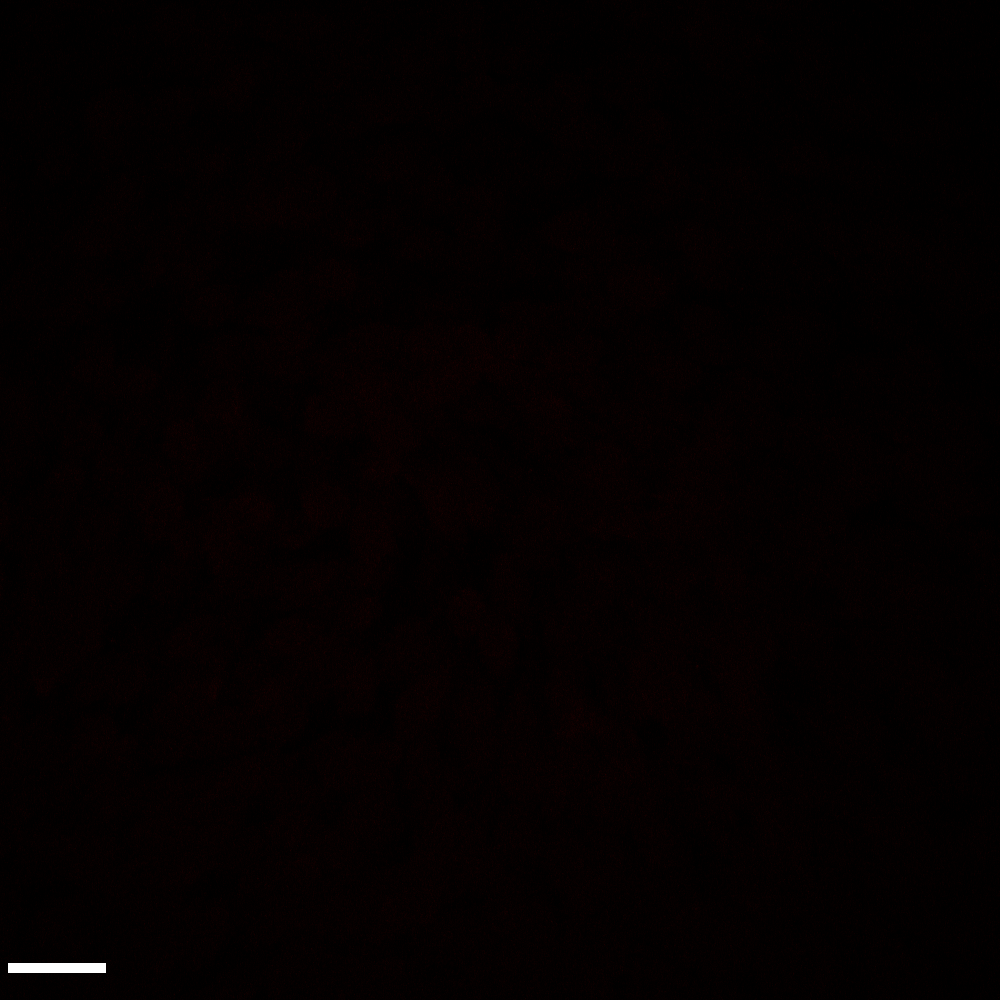

Supplement: Supplementary file 5 — Source data Fig. 3 [file 44321_2024_103_MOESM5_ESM.zip › Figure 3/3E/BHK-ZIKV mock E-protein.tif]

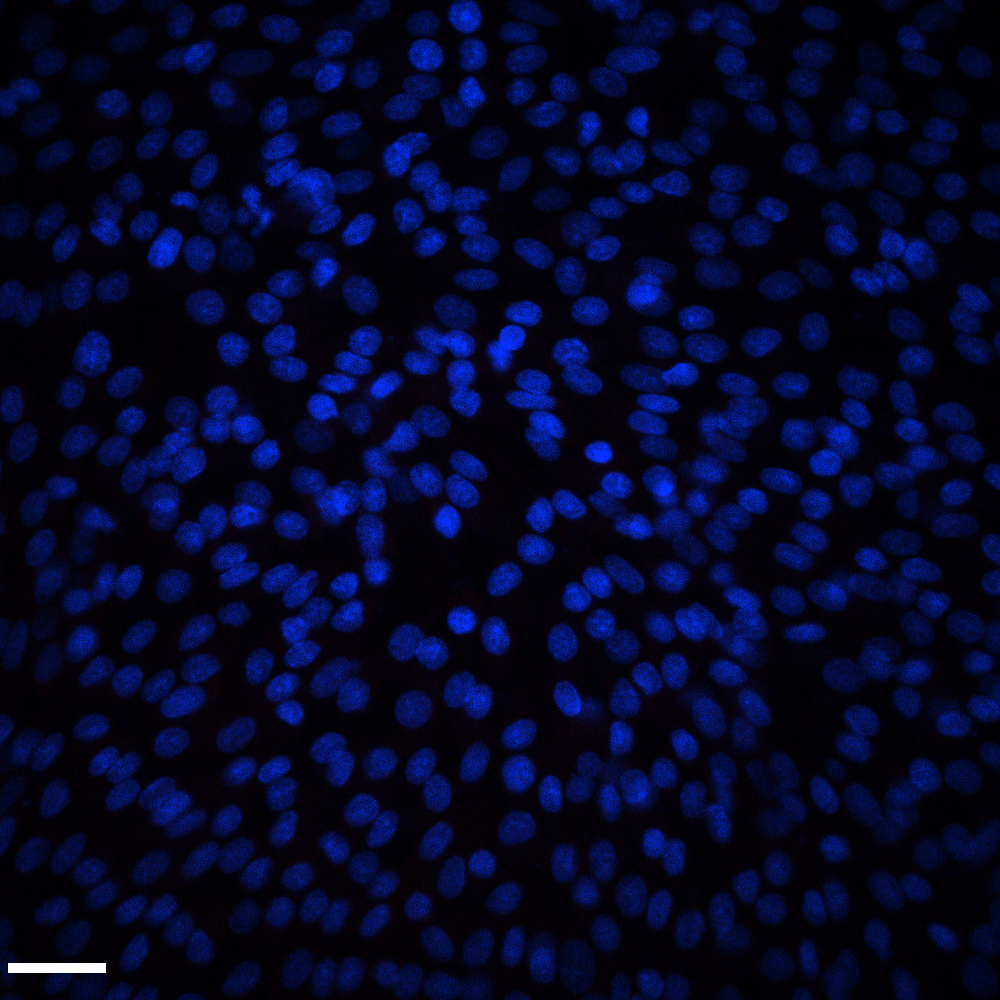

Supplement: Supplementary file 5 — Source data Fig. 3 [file 44321_2024_103_MOESM5_ESM.zip › Figure 3/3E/BHK-ZIKV mock MERGED.tif]

## Slide 1
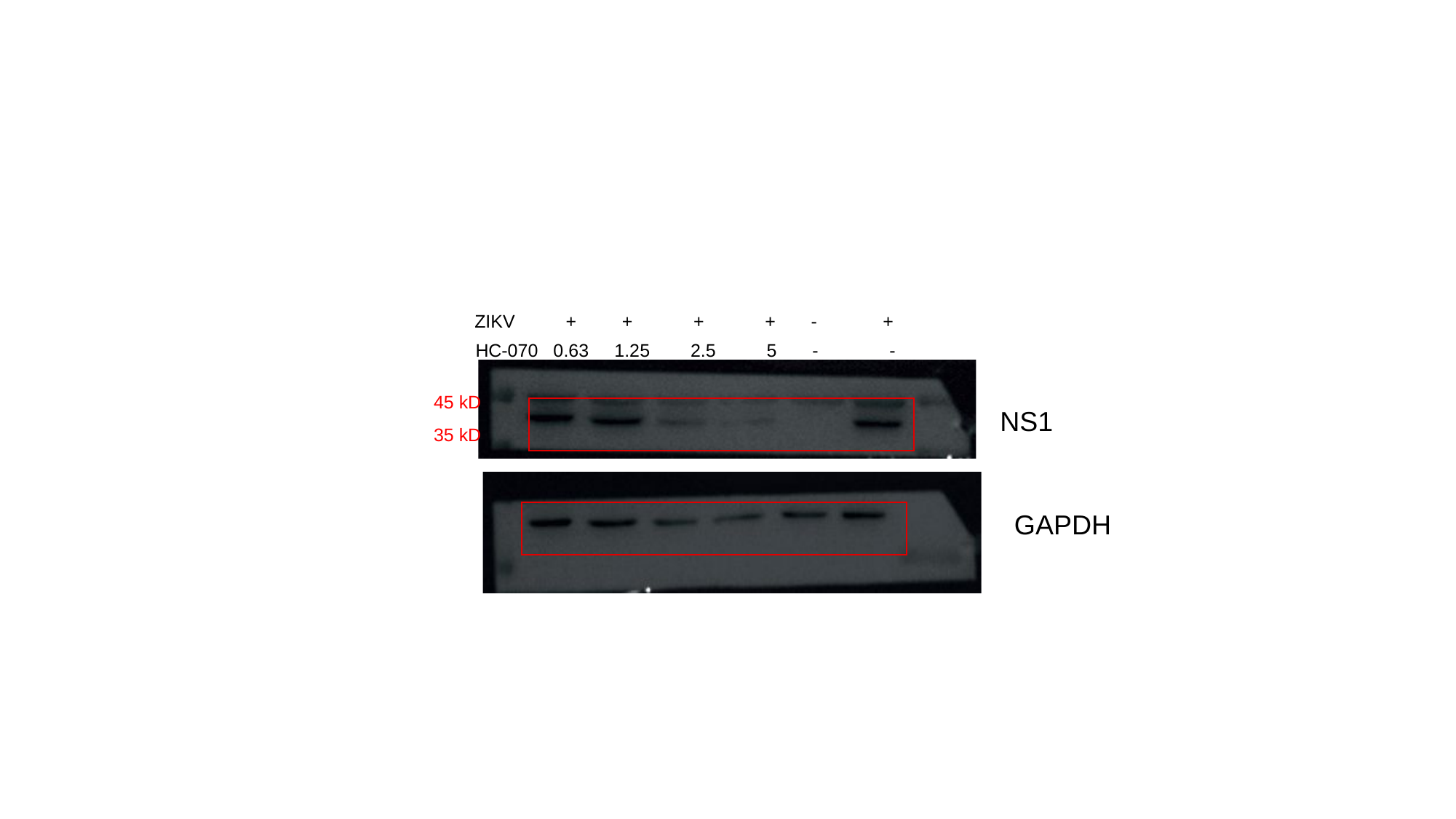

ZIKV + + + + - +
HC-070 0.63 1.25 2.5 5 - -
45 kD
NS1
35 kD
GAPDH

Supplement: Supplementary file 5 — Source data Fig. 3 [file 44321_2024_103_MOESM5_ESM.zip › Figure 3/3H/WB bands.pptx]

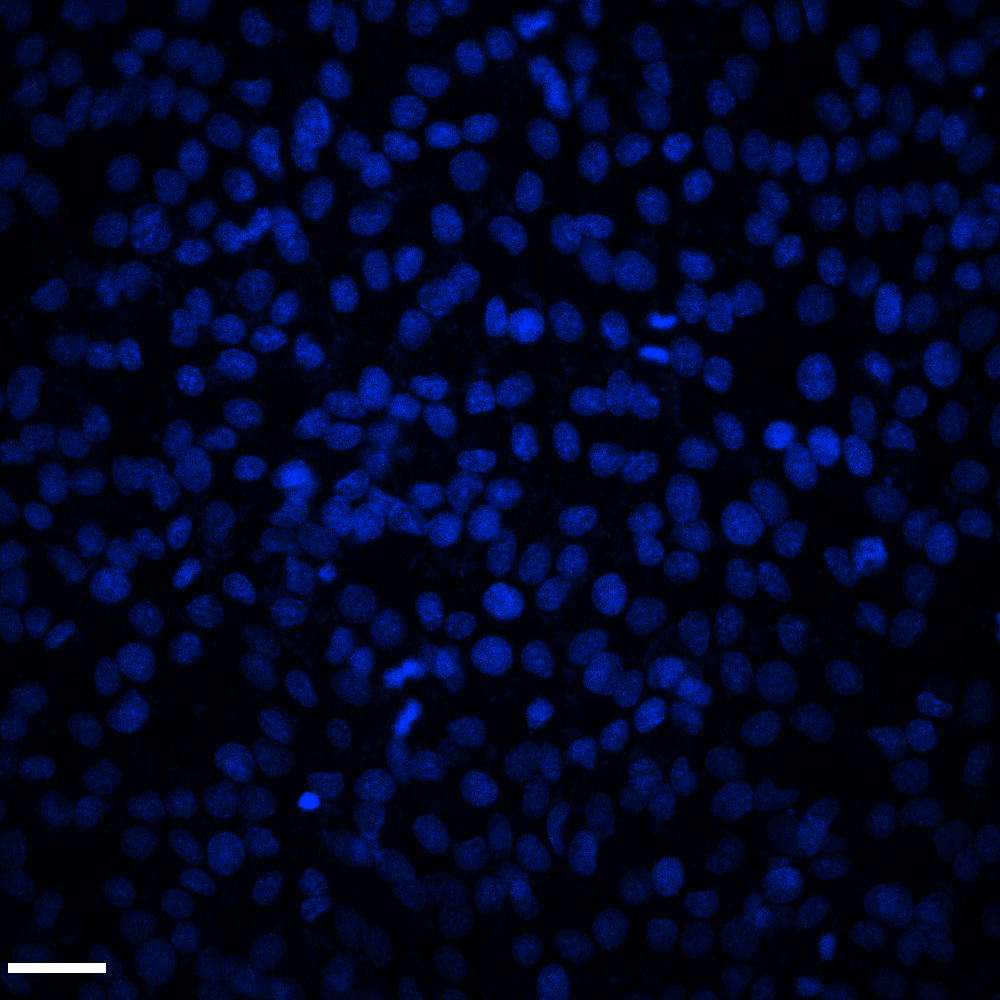

Supplement: Supplementary file 6 — Source data Fig. 4 [file 44321_2024_103_MOESM6_ESM.zip › Figure 4/4B/IF images/BHK-ZIKV 7DAY HC070 3 D.tif]

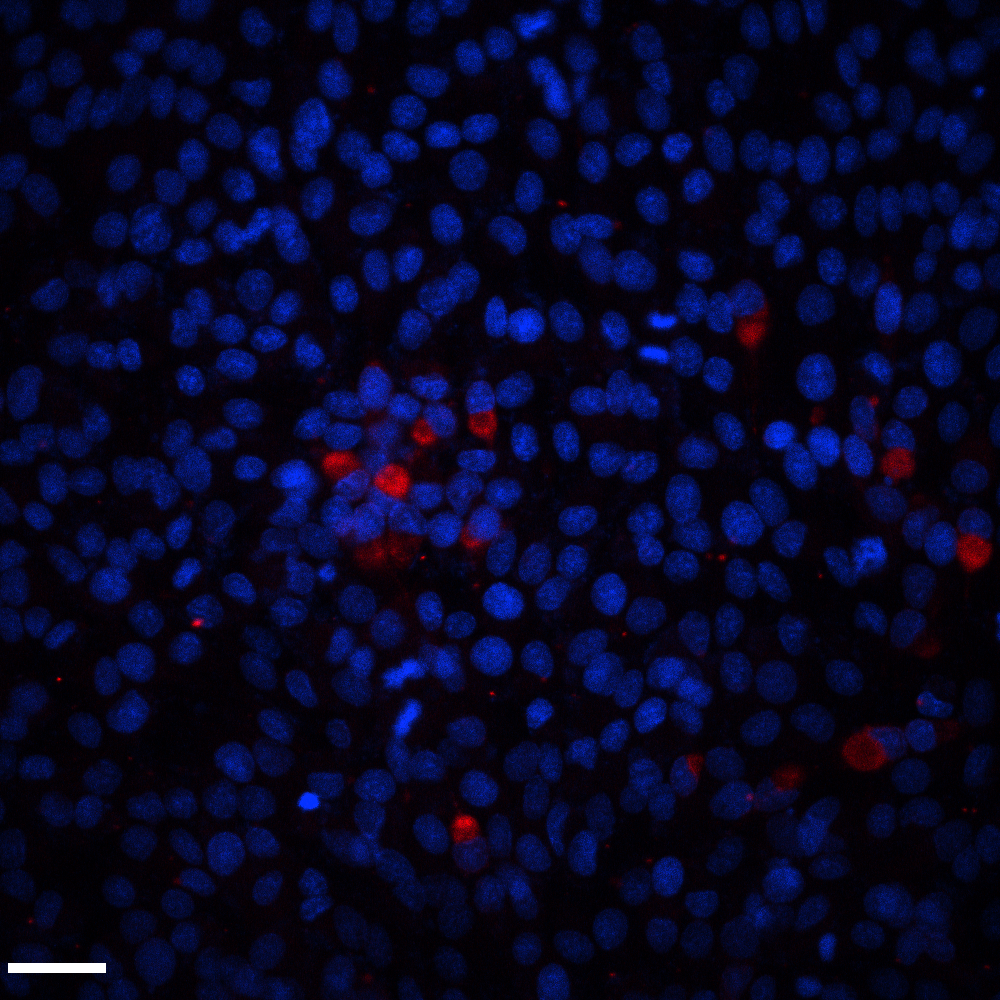

Supplement: Supplementary file 6 — Source data Fig. 4 [file 44321_2024_103_MOESM6_ESM.zip › Figure 4/4B/IF images/BHK-ZIKV 7DAY HC070 3 M.tif]

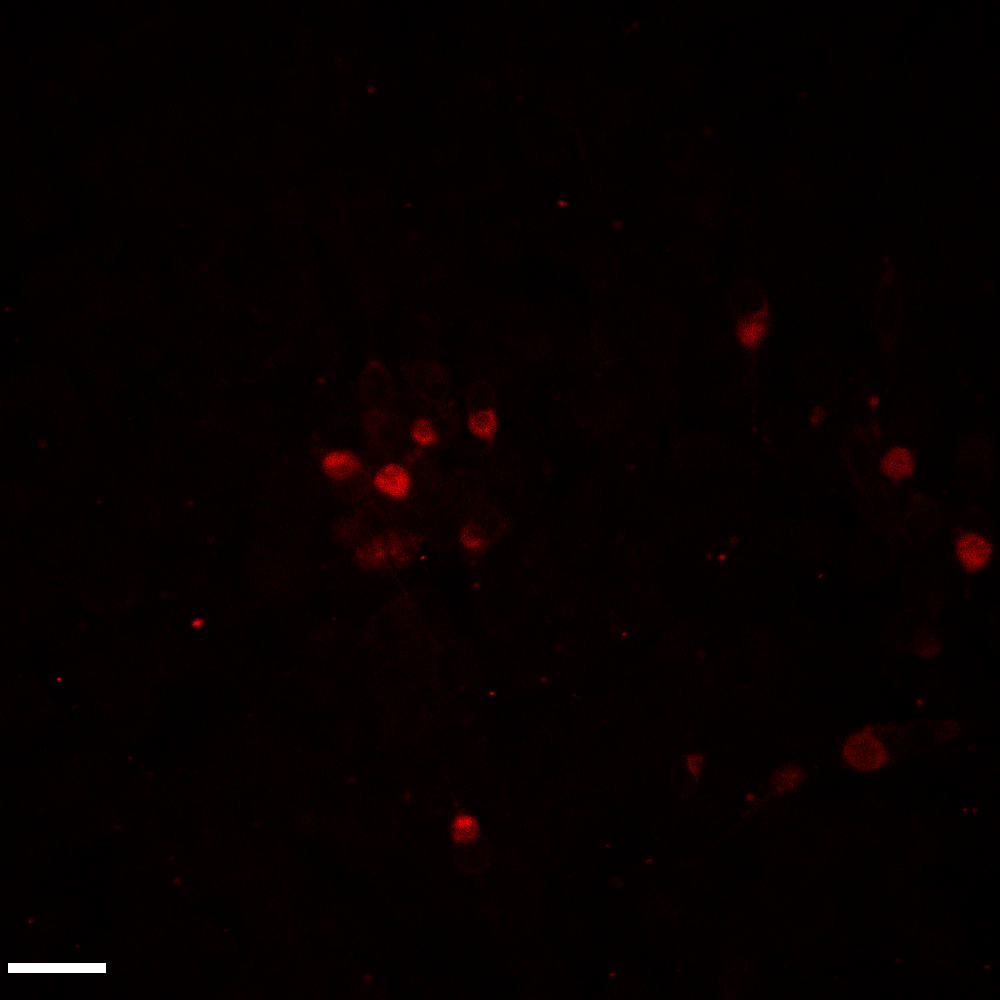

Supplement: Supplementary file 6 — Source data Fig. 4 [file 44321_2024_103_MOESM6_ESM.zip › Figure 4/4B/IF images/BHK-ZIKV 7DAY HC070 3 R.tif]

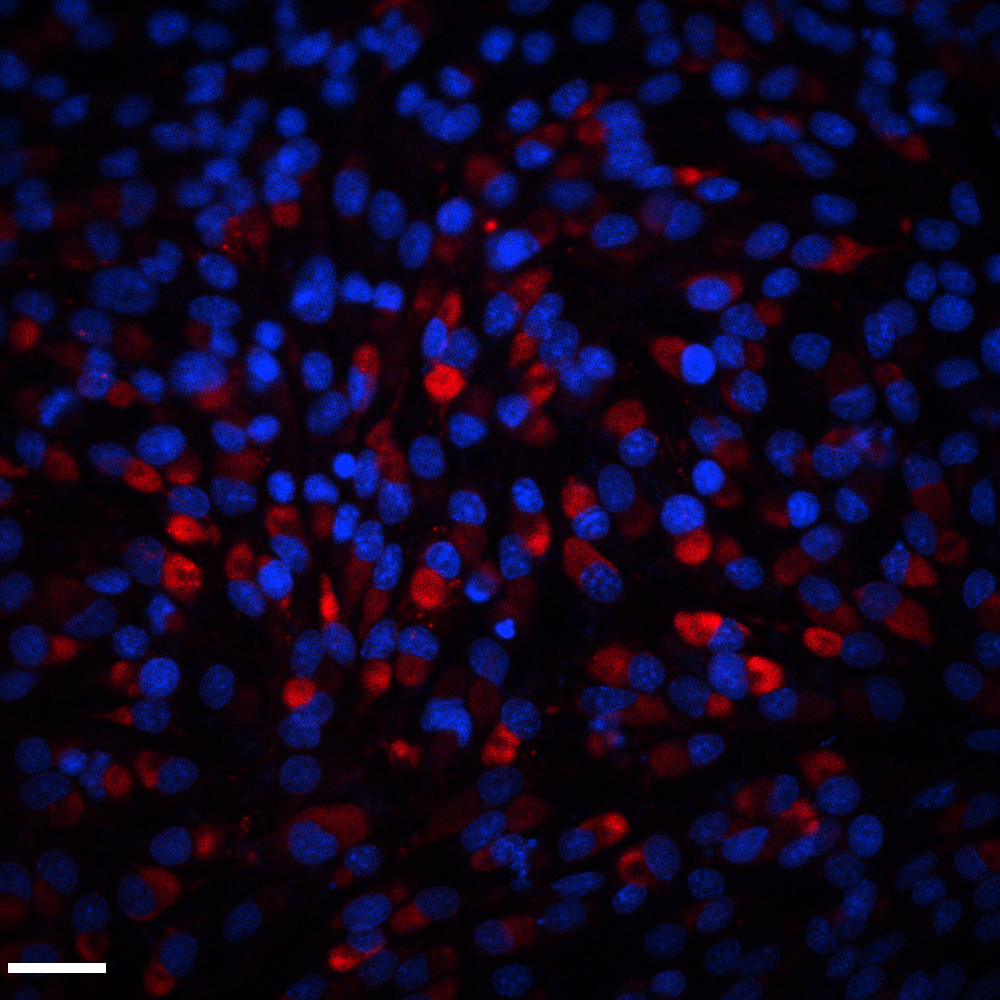

Supplement: Supplementary file 6 — Source data Fig. 4 [file 44321_2024_103_MOESM6_ESM.zip › Figure 4/4B/IF images/BHK-ZIKV 7DAY 4DMSO4 MERGED.tif]

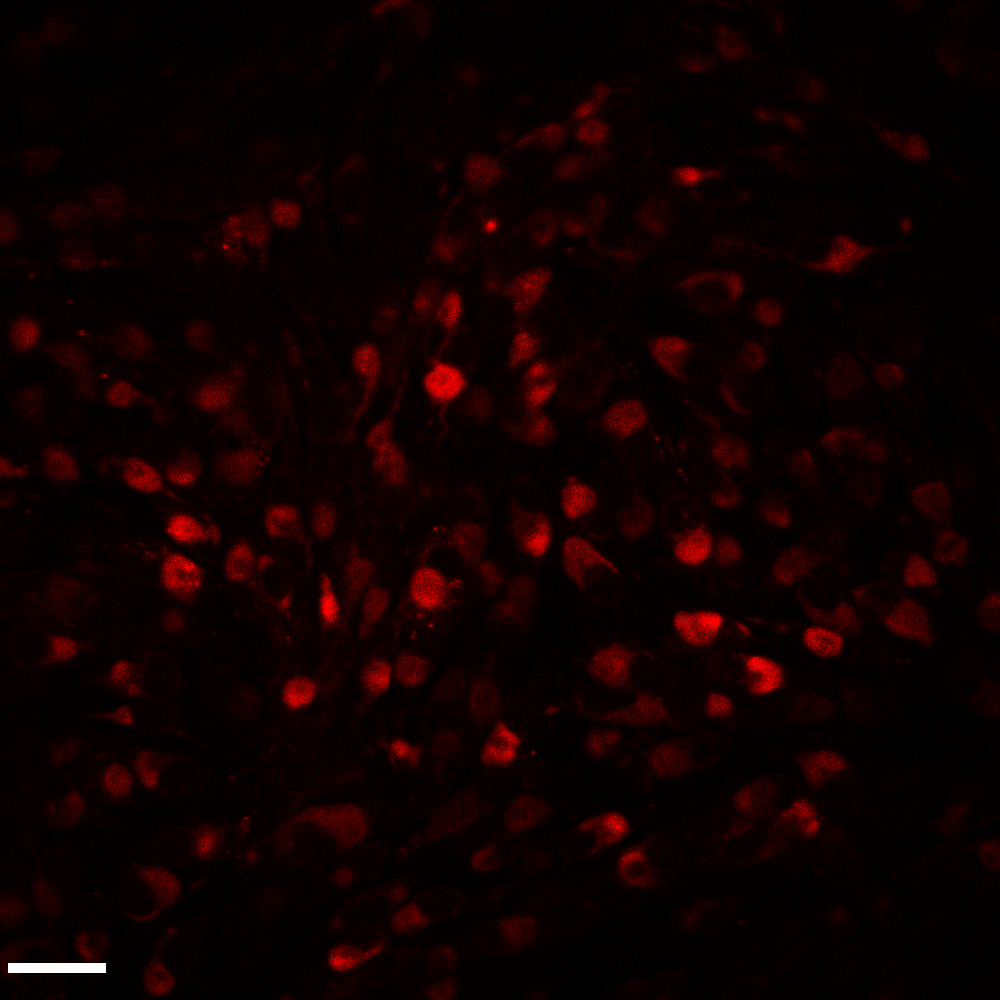

Supplement: Supplementary file 6 — Source data Fig. 4 [file 44321_2024_103_MOESM6_ESM.zip › Figure 4/4B/IF images/BHK-ZIKV 7DAY 4DMSO4 R.tif]

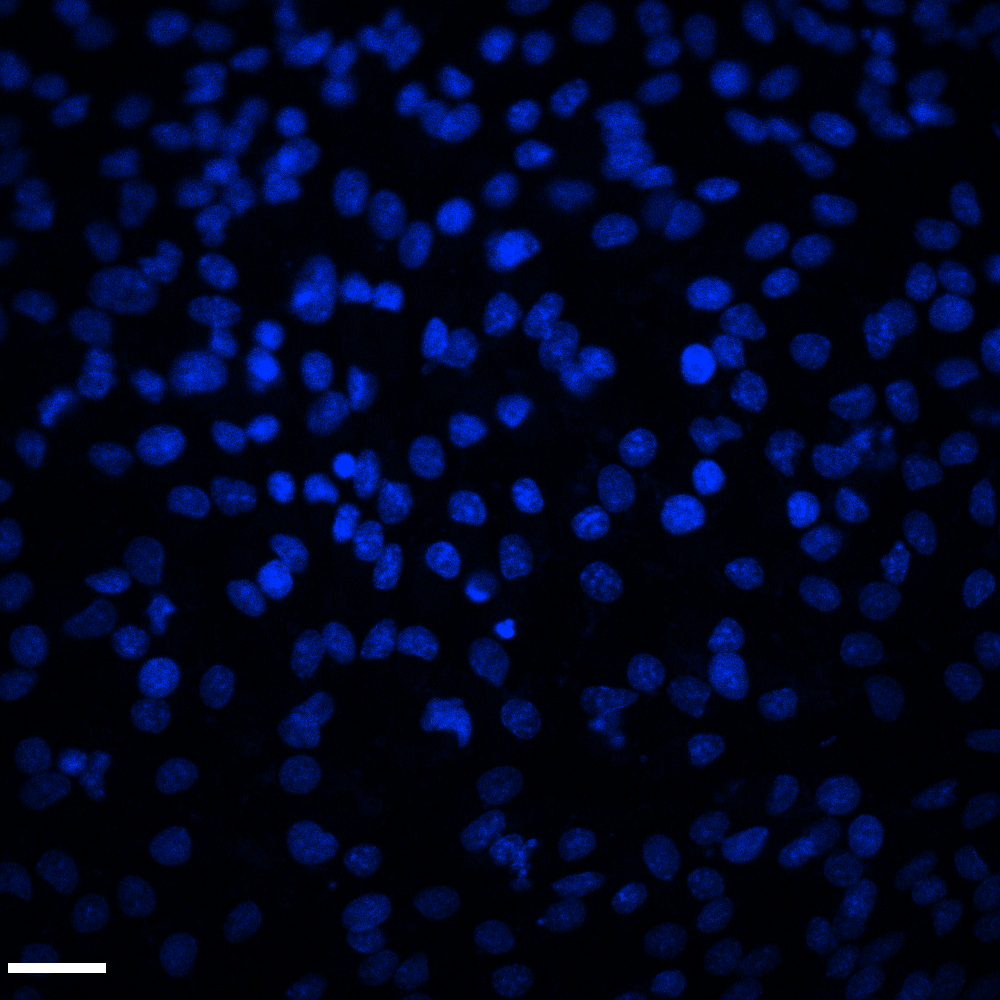

Supplement: Supplementary file 6 — Source data Fig. 4 [file 44321_2024_103_MOESM6_ESM.zip › Figure 4/4B/IF images/BHK-ZIKV 7DAY 4DMSO4.tif]

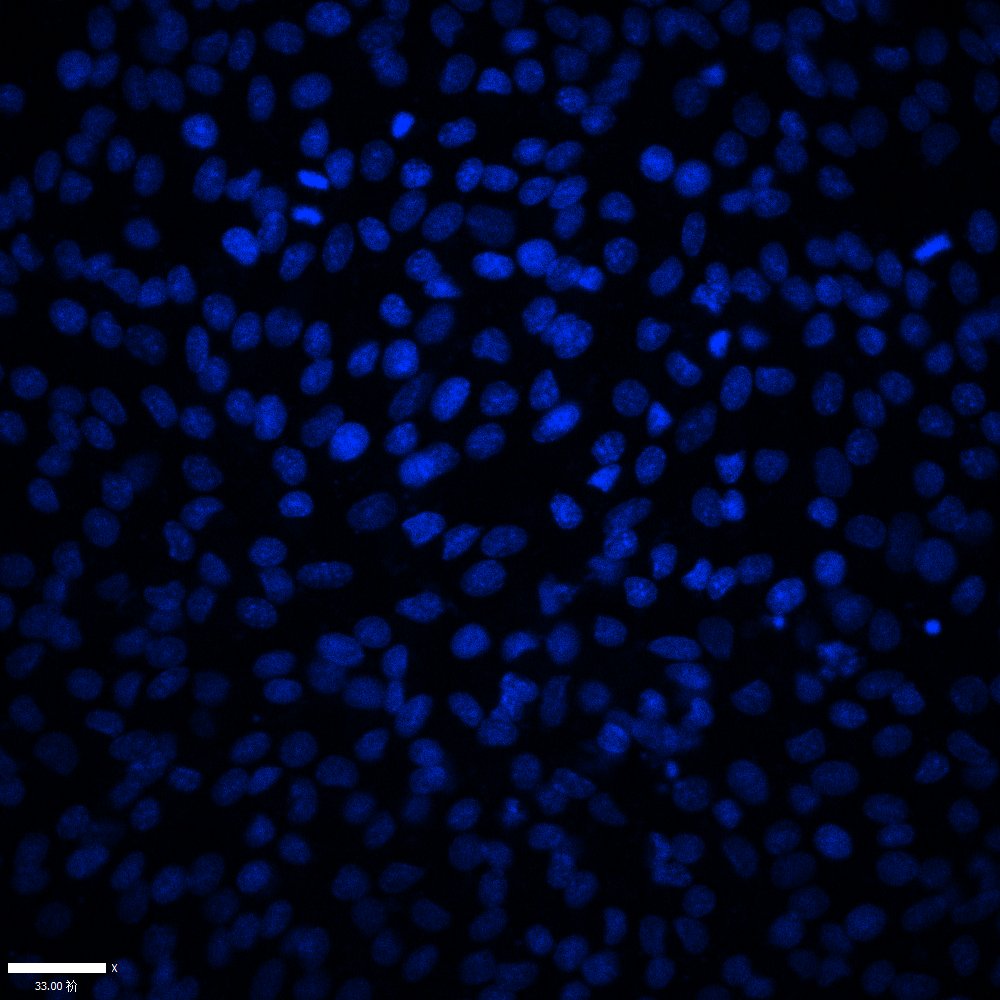

Supplement: Supplementary file 6 — Source data Fig. 4 [file 44321_2024_103_MOESM6_ESM.zip › Figure 4/4B/IF images/BHK-ZIKV 7DAY EGTA d.tif]

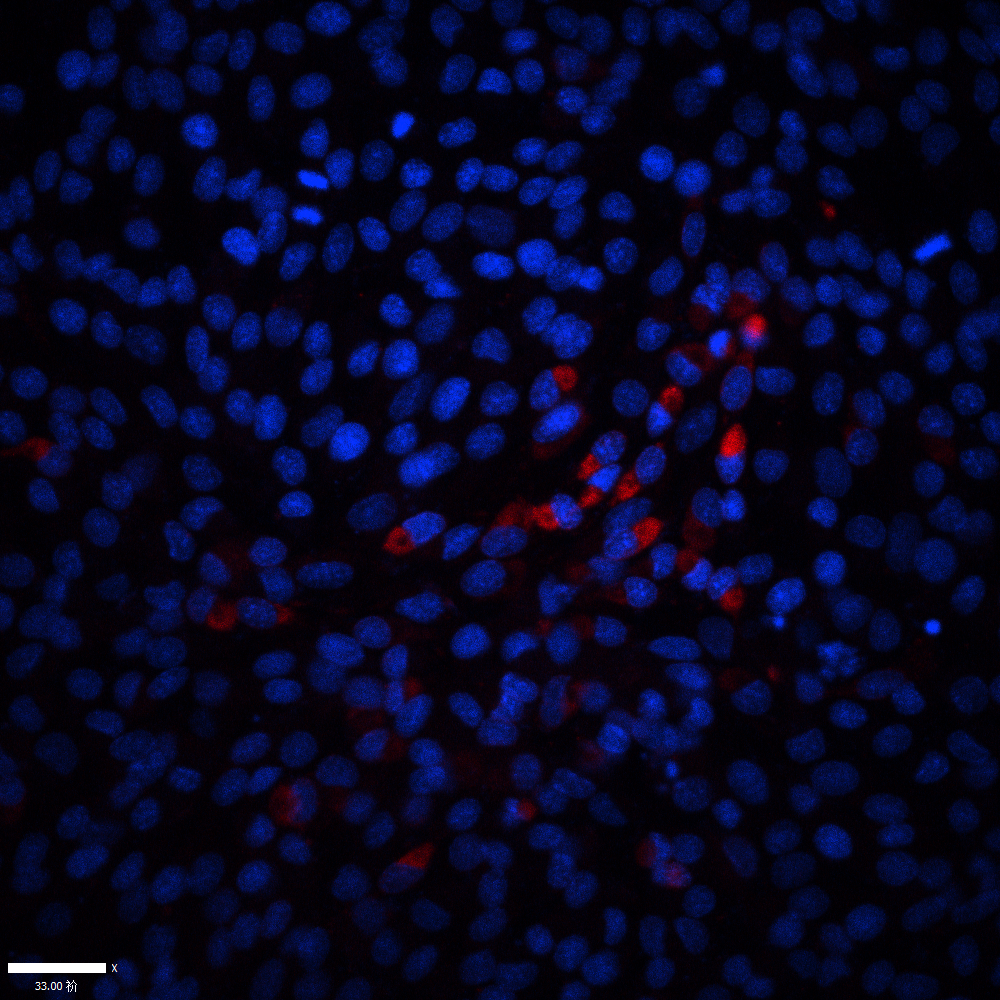

Supplement: Supplementary file 6 — Source data Fig. 4 [file 44321_2024_103_MOESM6_ESM.zip › Figure 4/4B/IF images/BHK-ZIKV 7DAY EGTA m.tif]

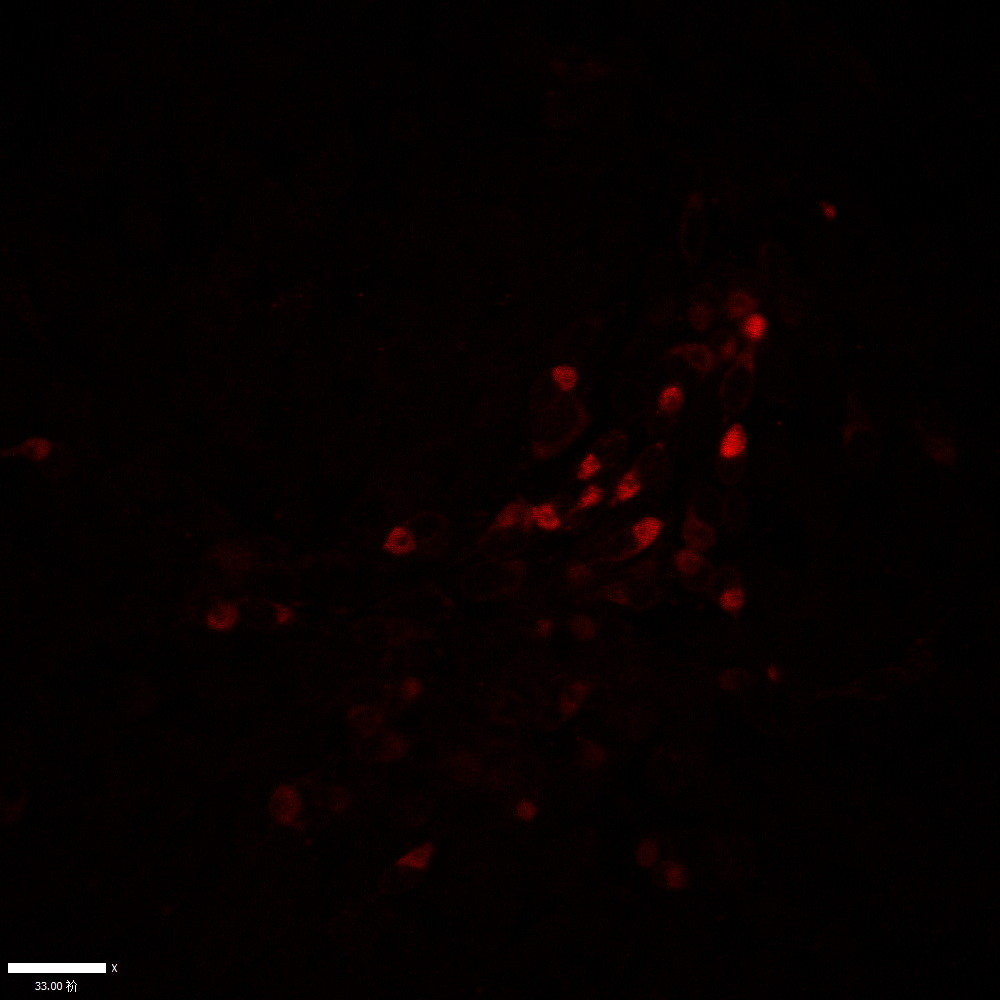

Supplement: Supplementary file 6 — Source data Fig. 4 [file 44321_2024_103_MOESM6_ESM.zip › Figure 4/4B/IF images/BHK-ZIKV 7DAY EGTA R.tif]

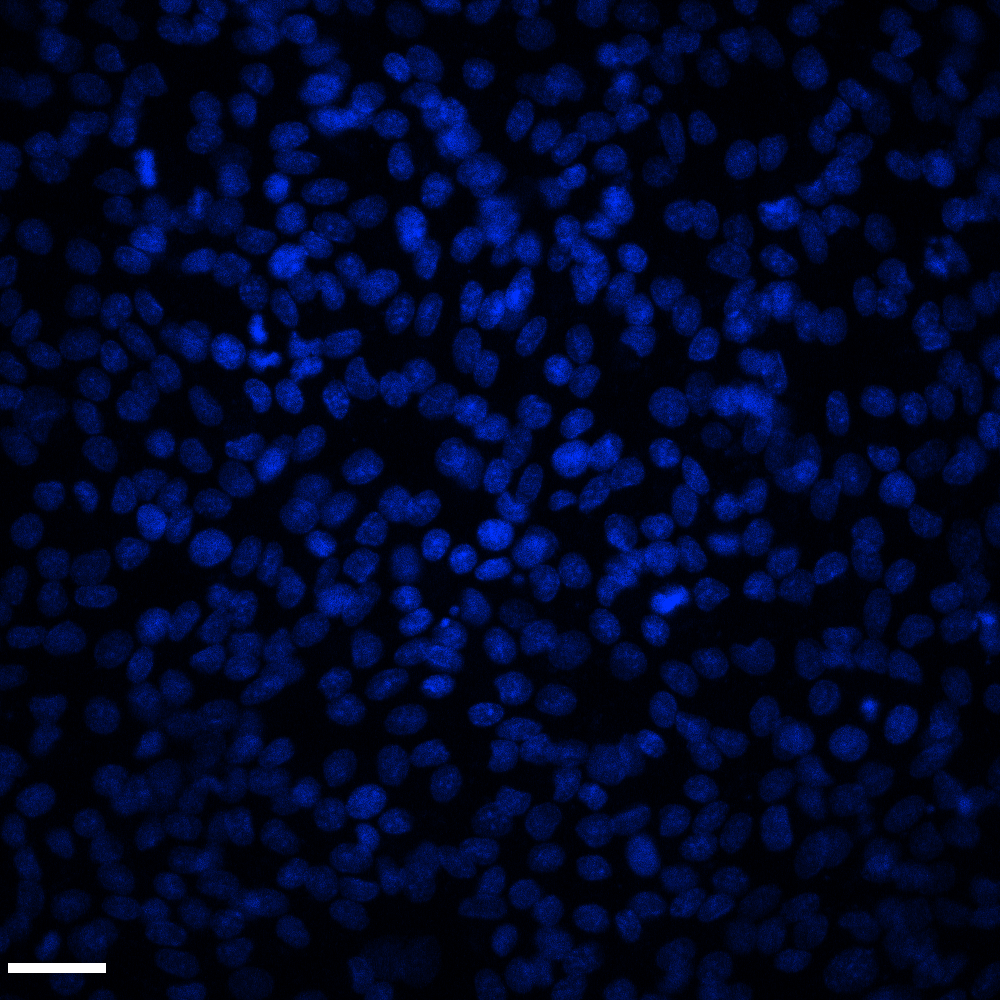

Supplement: Supplementary file 6 — Source data Fig. 4 [file 44321_2024_103_MOESM6_ESM.zip › Figure 4/4B/IF images/BHK-ZIKV 7DAY KN93 D.tif]

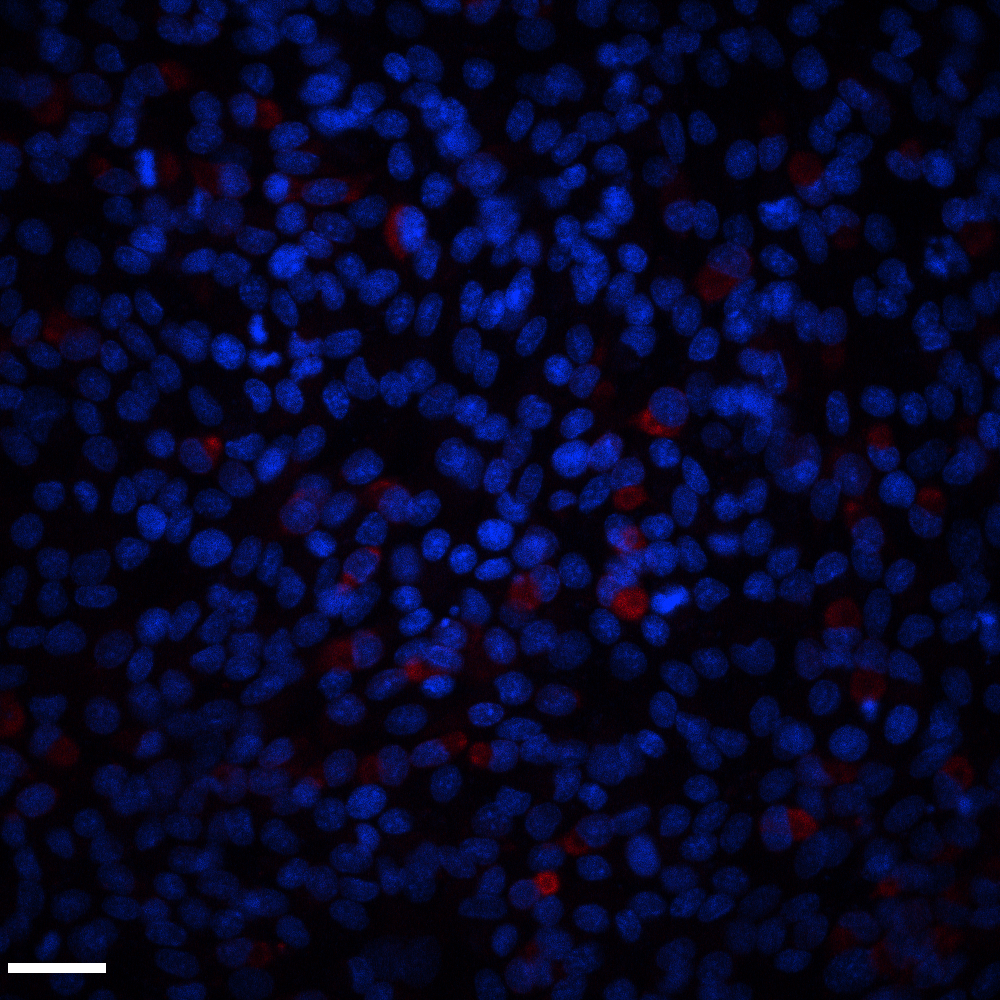

Supplement: Supplementary file 6 — Source data Fig. 4 [file 44321_2024_103_MOESM6_ESM.zip › Figure 4/4B/IF images/BHK-ZIKV 7DAY KN93 M.tif]

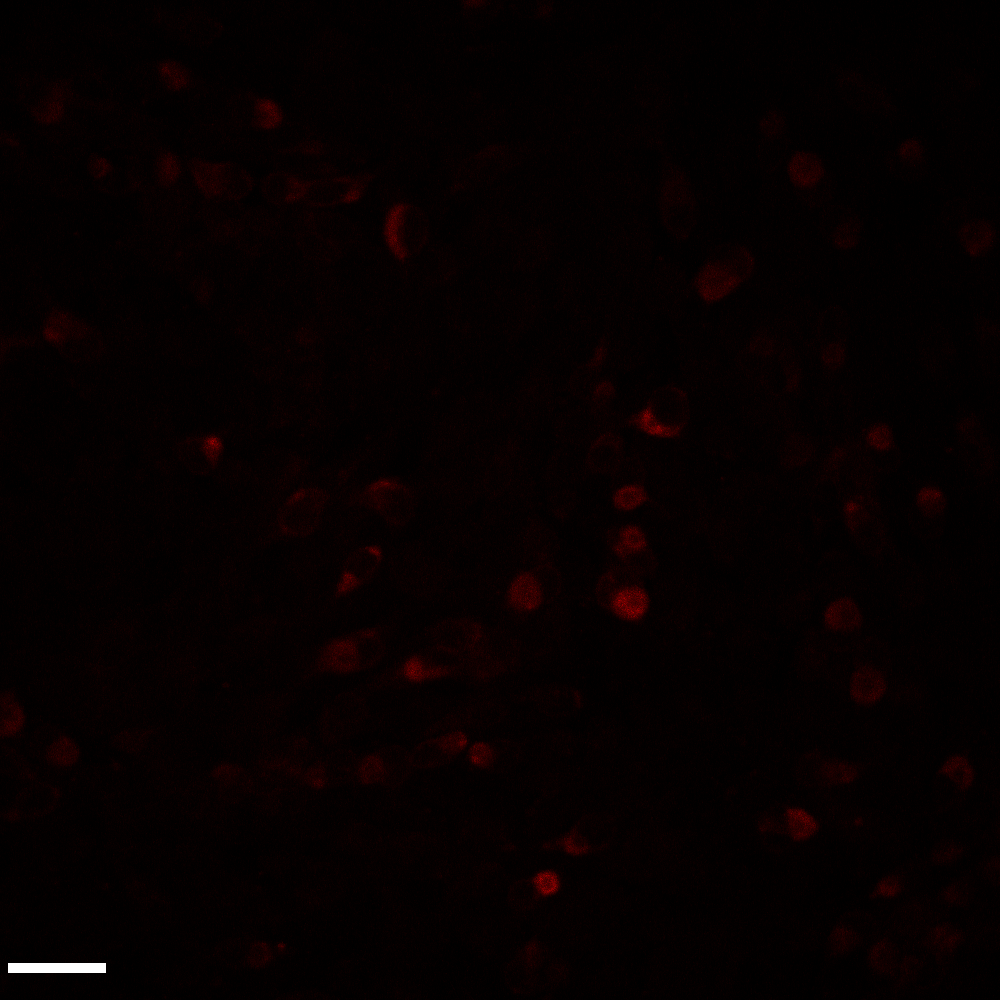

Supplement: Supplementary file 6 — Source data Fig. 4 [file 44321_2024_103_MOESM6_ESM.zip › Figure 4/4B/IF images/BHK-ZIKV 7DAY KN93 R.tif]

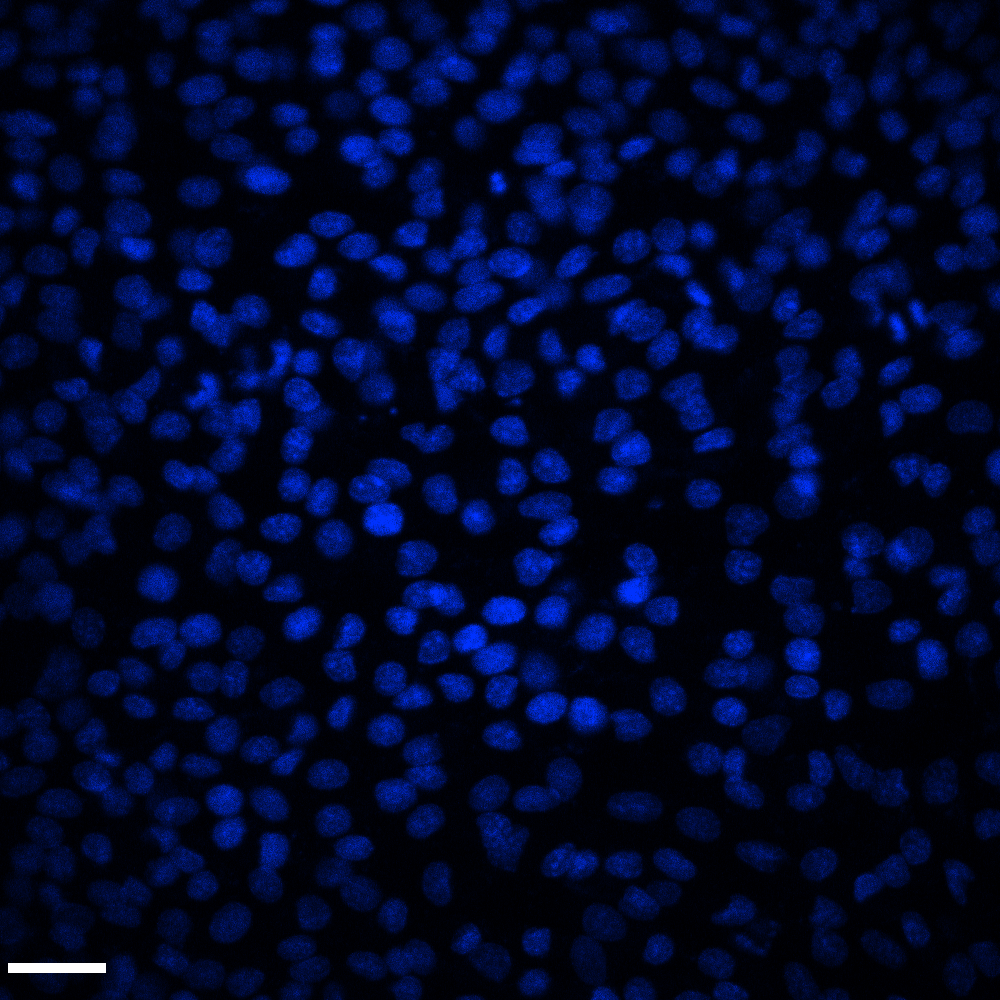

Supplement: Supplementary file 6 — Source data Fig. 4 [file 44321_2024_103_MOESM6_ESM.zip › Figure 4/4B/IF images/BHK-ZIKV 7DAY MOCK D.tif]

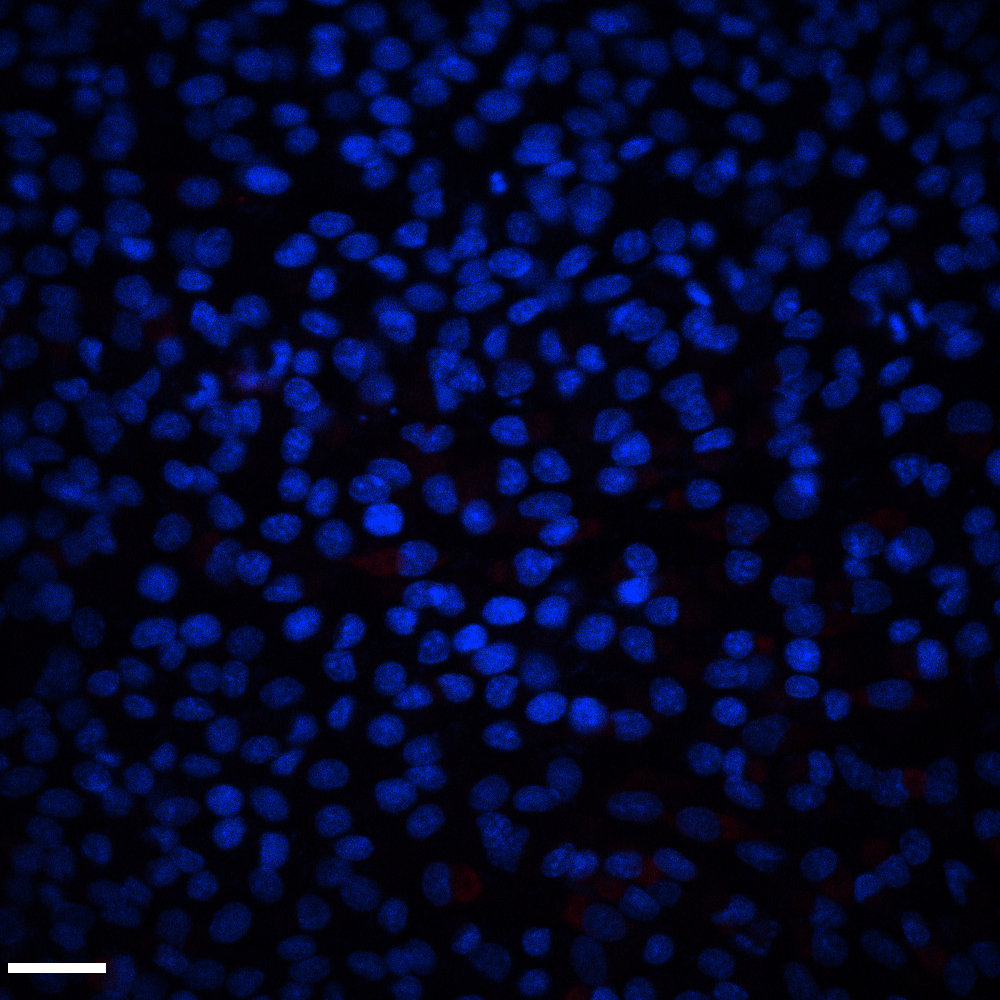

Supplement: Supplementary file 6 — Source data Fig. 4 [file 44321_2024_103_MOESM6_ESM.zip › Figure 4/4B/IF images/BHK-ZIKV 7DAY MOCK M.tif]

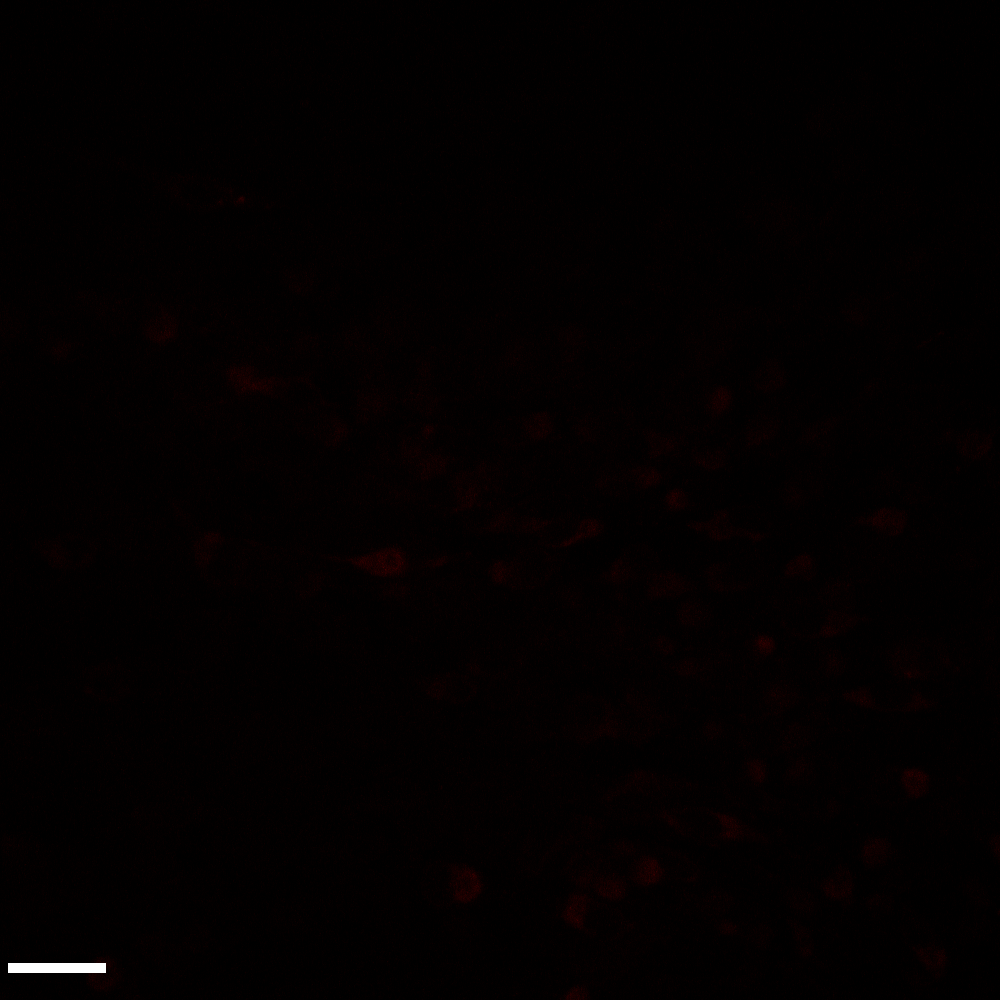

Supplement: Supplementary file 6 — Source data Fig. 4 [file 44321_2024_103_MOESM6_ESM.zip › Figure 4/4B/IF images/BHK-ZIKV 7DAY MOCK R.tif]

## Slide 1
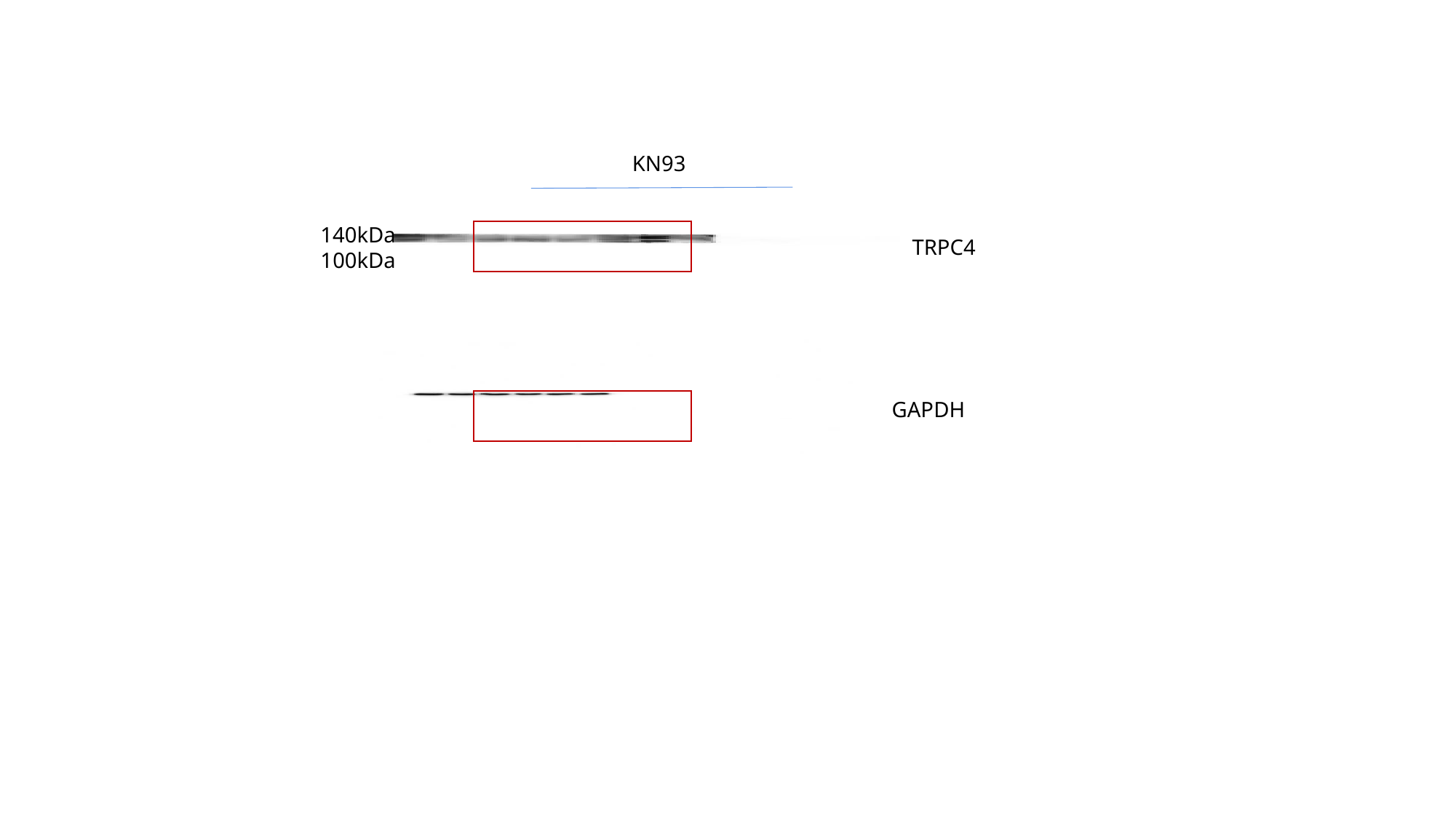

KN93
140kDa
100kDa
TRPC4
GAPDH

Supplement: Supplementary file 6 — Source data Fig. 4 [file 44321_2024_103_MOESM6_ESM.zip › Figure 4/4E/WB bands.pptx]

## Slide 1
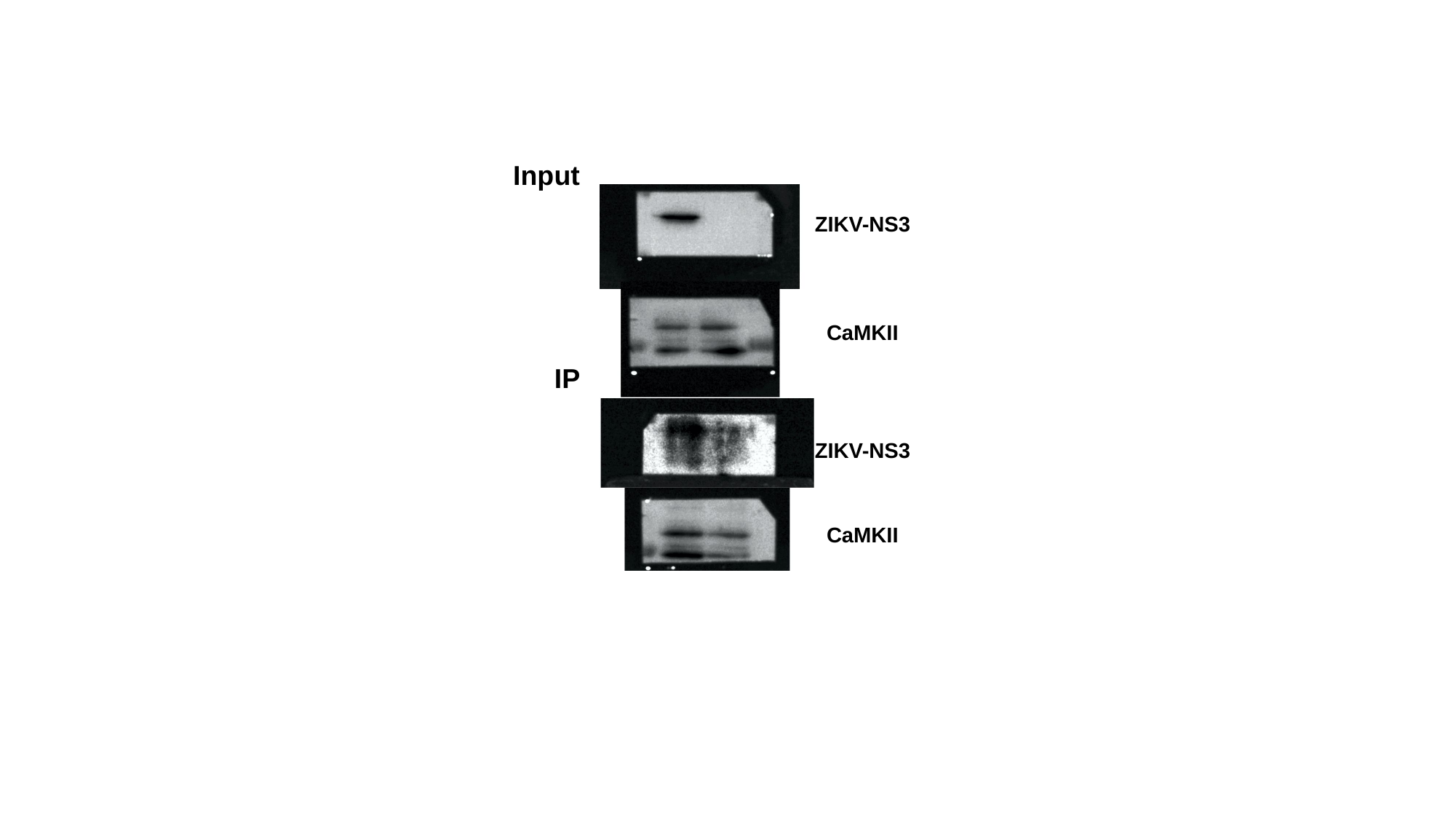

Input
ZIKV-NS3
CaMKII
IP
ZIKV-NS3
CaMKII

Supplement: Supplementary file 6 — Source data Fig. 4 [file 44321_2024_103_MOESM6_ESM.zip › Figure 4/4F/WB bands.pptx]

## Slide 1
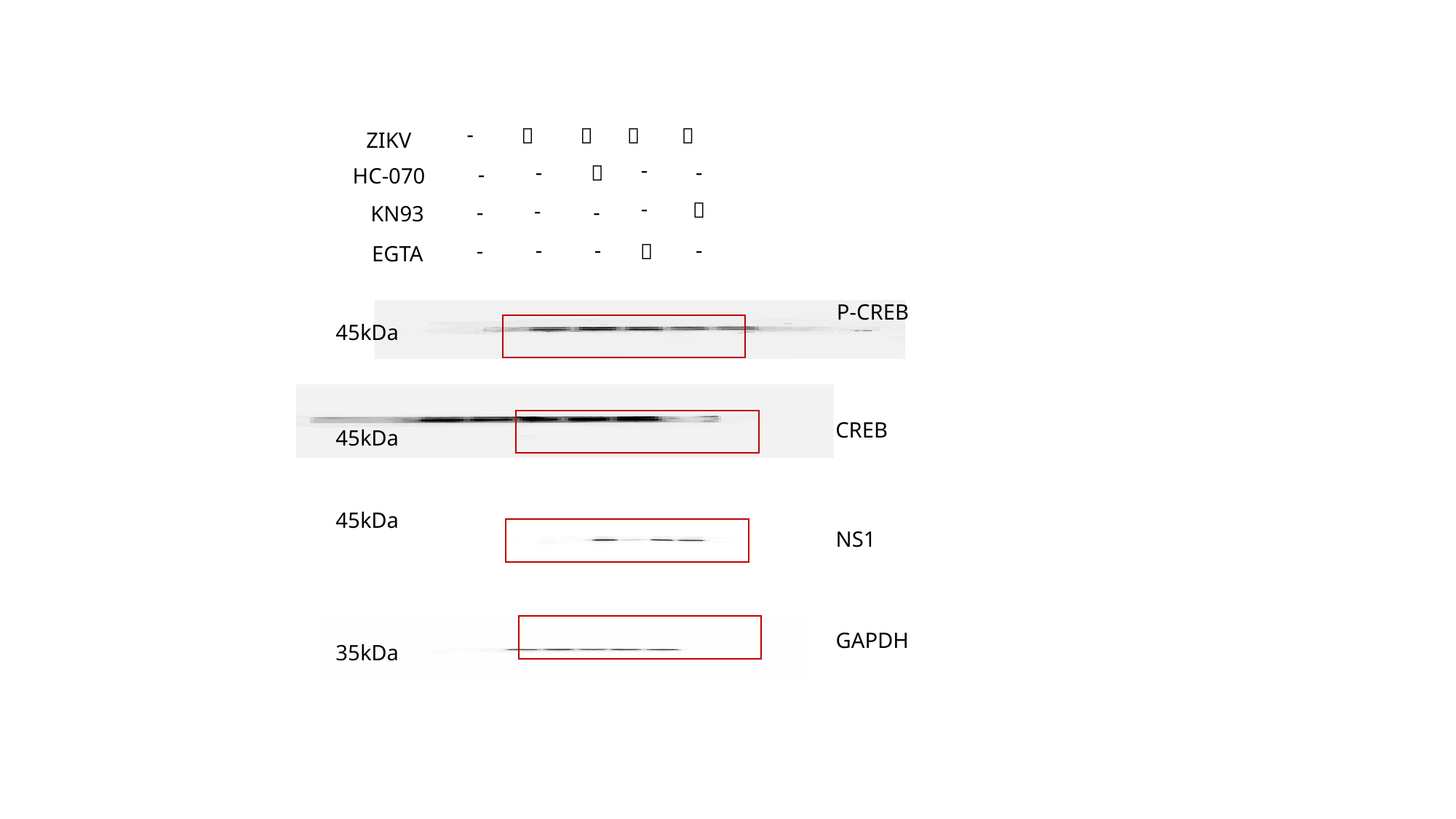

-
＋
＋
＋
＋
ZIKV
-
-
-
＋
-
HC-070
-
＋
-
-
-
KN93
-
-
-
-
＋
EGTA
P-CREB
45kDa
CREB
45kDa
45kDa
NS1
GAPDH
35kDa

Supplement: Supplementary file 6 — Source data Fig. 4 [file 44321_2024_103_MOESM6_ESM.zip › Figure 4/4H/WB bands.pptx]

## Slide 1
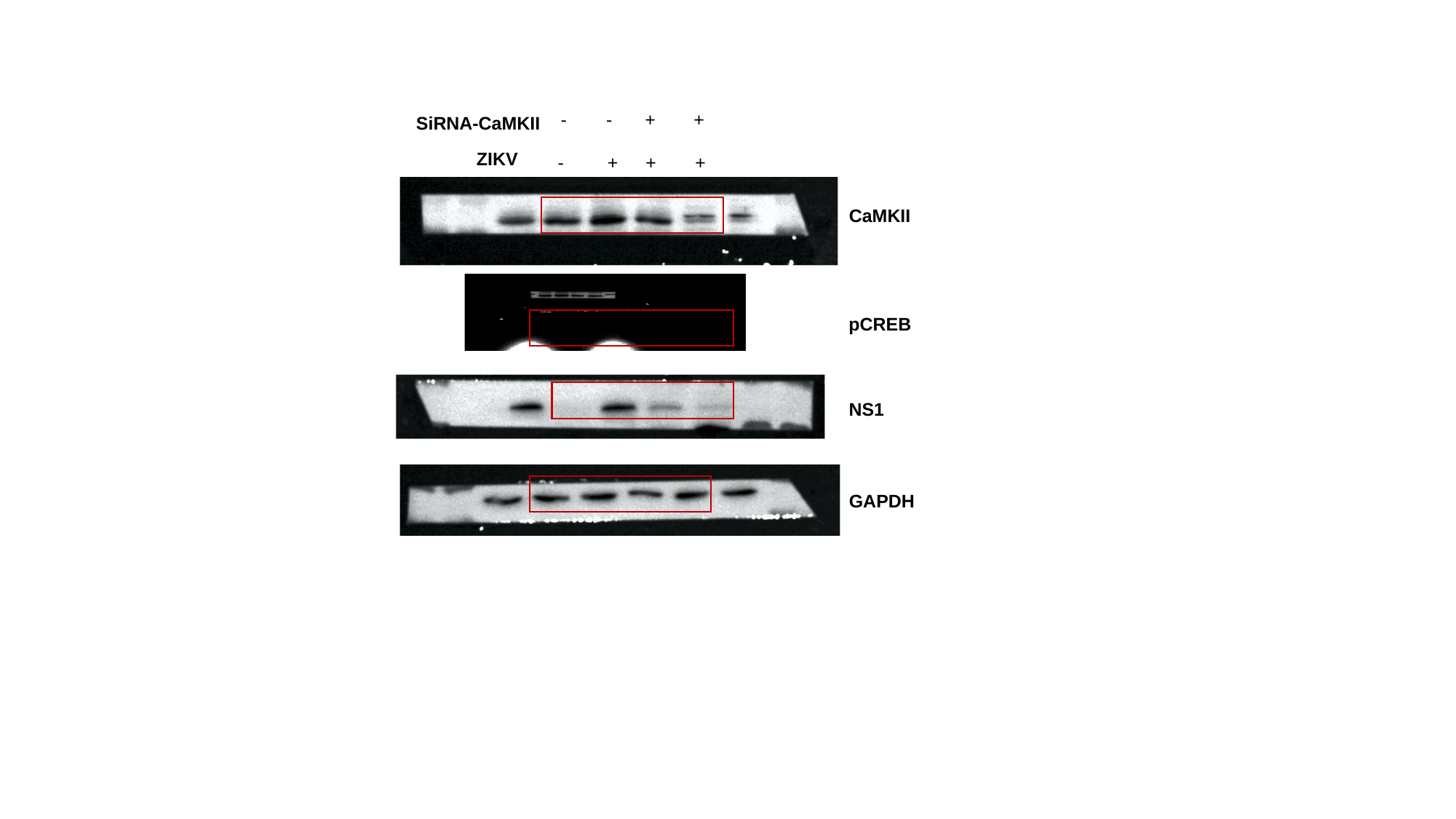

-
-
+
+
SiRNA-CaMKII
ZIKV
-
+
+
+
CaMKII
pCREB
NS1
GAPDH

Supplement: Supplementary file 6 — Source data Fig. 4 [file 44321_2024_103_MOESM6_ESM.zip › Figure 4/4I and J/WB bands.pptx]

## Slide 1
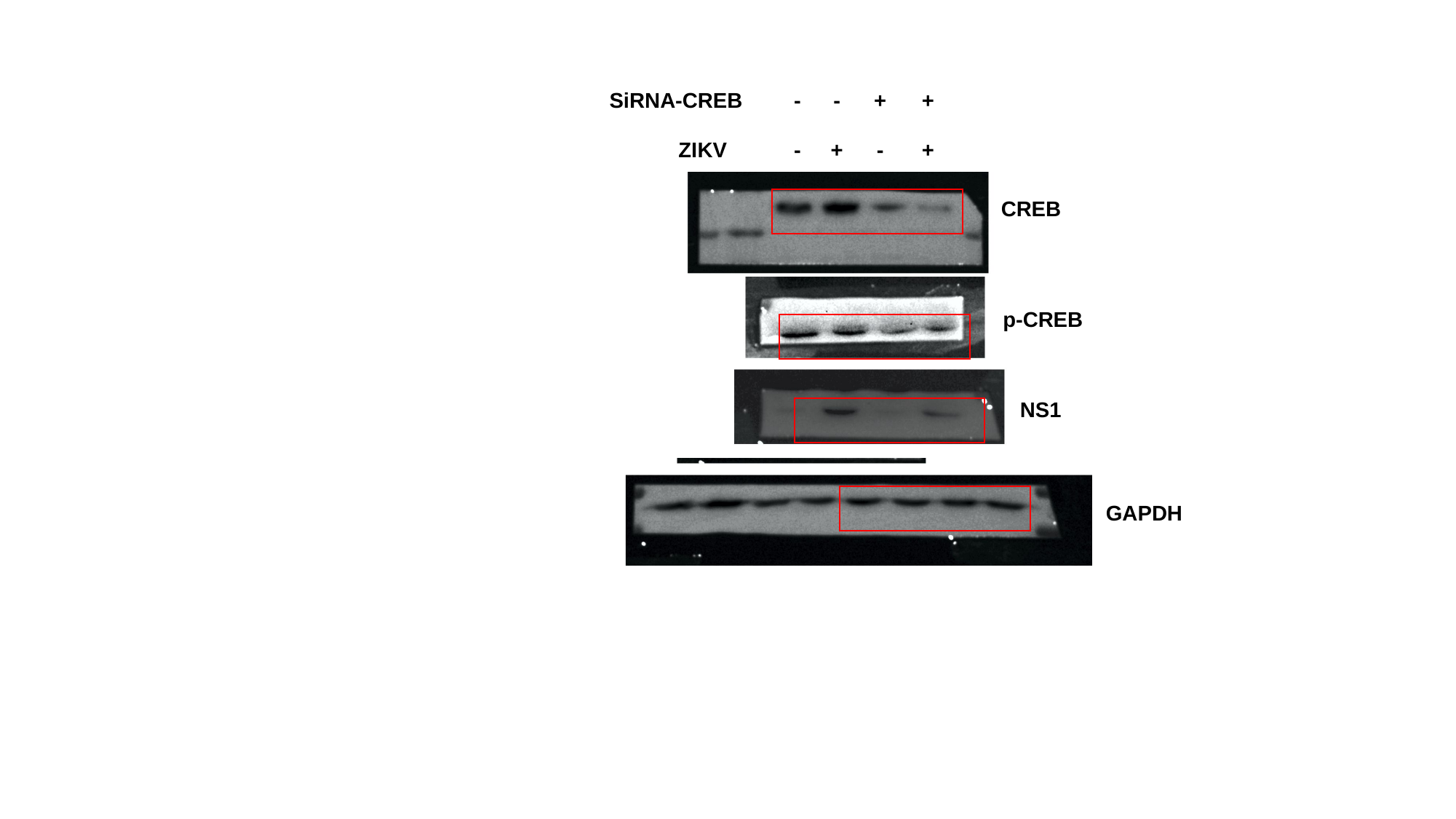

SiRNA-CREB
-
-
+
+
ZIKV
-
+
-
+
CREB
p-CREB
NS1
GAPDH

Supplement: Supplementary file 6 — Source data Fig. 4 [file 44321_2024_103_MOESM6_ESM.zip › Figure 4/4K and L/WB bands.pptx]

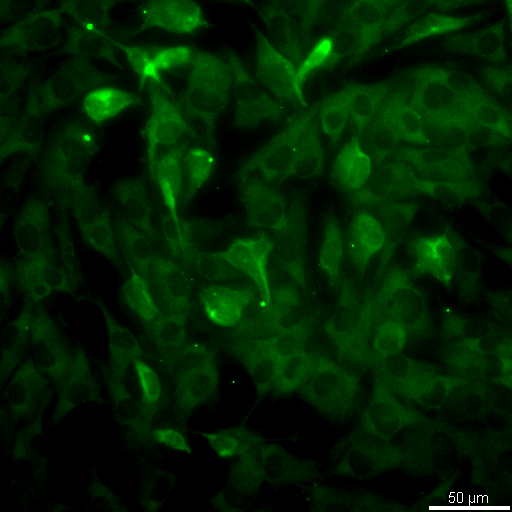

Supplement: Supplementary file 7 — Source data Fig. 5 [file 44321_2024_103_MOESM7_ESM.zip › Figure 5/5D/Mock-DDX3X.tif]

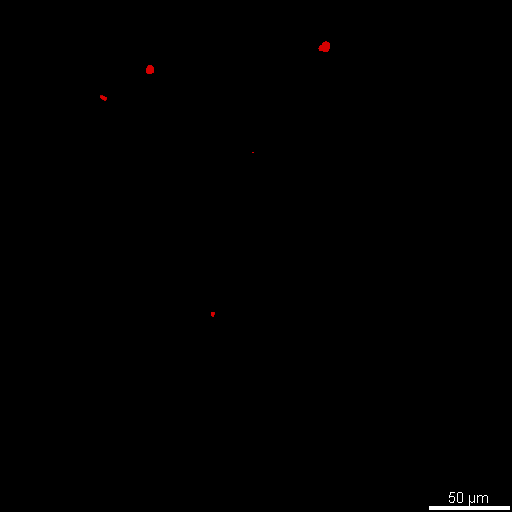

Supplement: Supplementary file 7 — Source data Fig. 5 [file 44321_2024_103_MOESM7_ESM.zip › Figure 5/5D/Mock-E protein.tif]

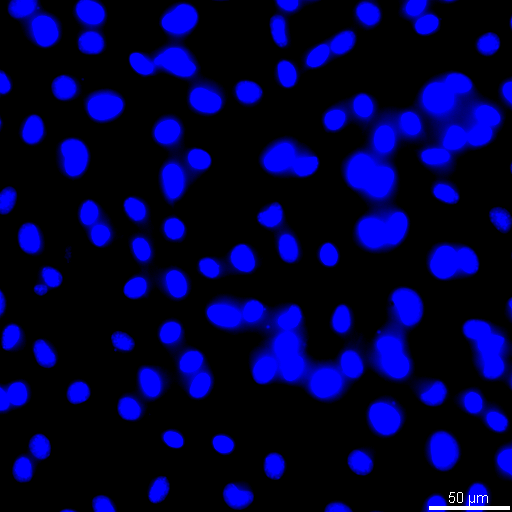

Supplement: Supplementary file 7 — Source data Fig. 5 [file 44321_2024_103_MOESM7_ESM.zip › Figure 5/5D/Mock-hoechst.tif]

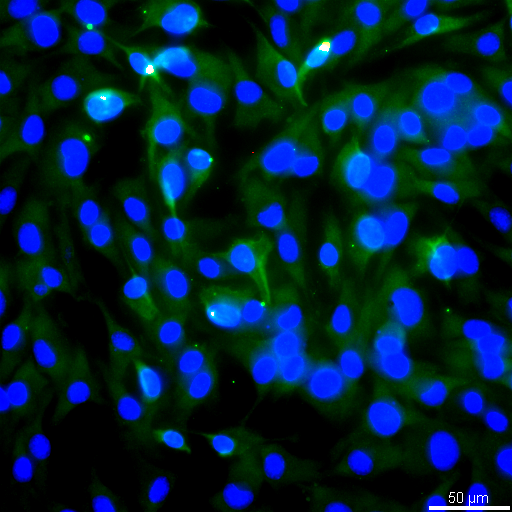

Supplement: Supplementary file 7 — Source data Fig. 5 [file 44321_2024_103_MOESM7_ESM.zip › Figure 5/5D/Mock-merge.tif]

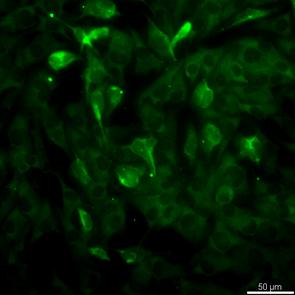

Supplement: Supplementary file 7 — Source data Fig. 5 [file 44321_2024_103_MOESM7_ESM.zip › Figure 5/5D/ZIKV+EGTA-DDX3X.jpg]

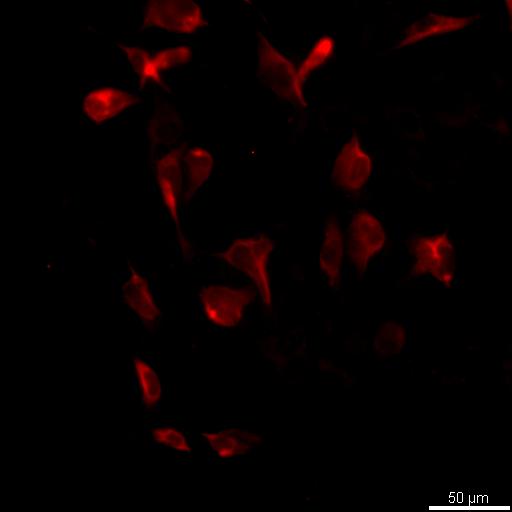

Supplement: Supplementary file 7 — Source data Fig. 5 [file 44321_2024_103_MOESM7_ESM.zip › Figure 5/5D/ZIKV+EGTA-E protein.jpg]

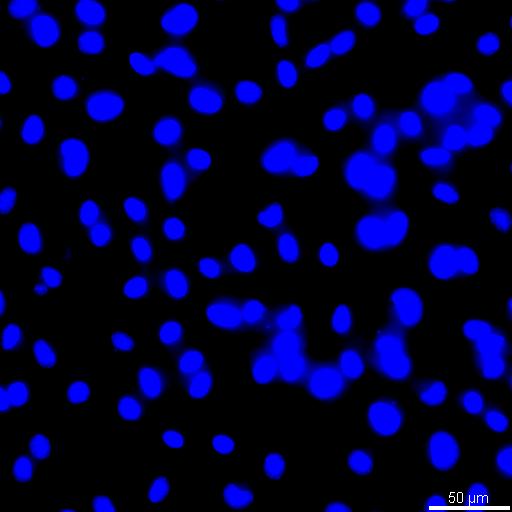

Supplement: Supplementary file 7 — Source data Fig. 5 [file 44321_2024_103_MOESM7_ESM.zip › Figure 5/5D/ZIKV+EGTA-hoechst.jpg]

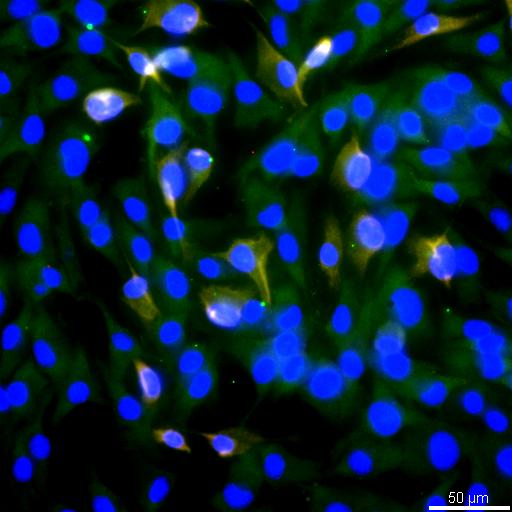

Supplement: Supplementary file 7 — Source data Fig. 5 [file 44321_2024_103_MOESM7_ESM.zip › Figure 5/5D/ZIKV+EGTA-merge.jpg]

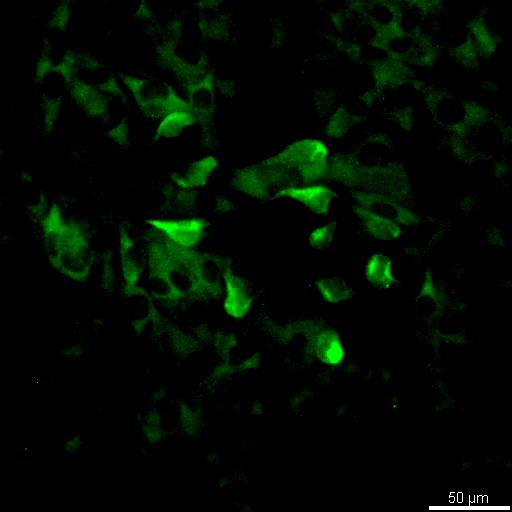

Supplement: Supplementary file 7 — Source data Fig. 5 [file 44321_2024_103_MOESM7_ESM.zip › Figure 5/5D/ZIKV+HC-070-DDX3X.jpg]

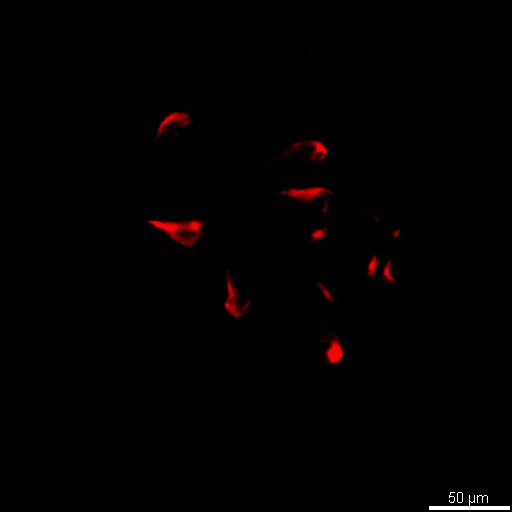

Supplement: Supplementary file 7 — Source data Fig. 5 [file 44321_2024_103_MOESM7_ESM.zip › Figure 5/5D/ZIKV+HC-070-E protein.jpg]

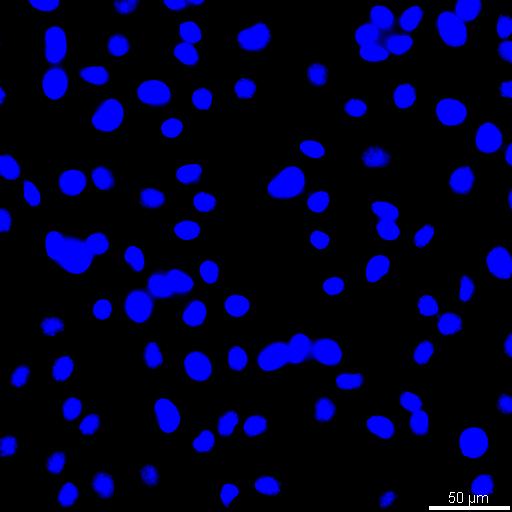

Supplement: Supplementary file 7 — Source data Fig. 5 [file 44321_2024_103_MOESM7_ESM.zip › Figure 5/5D/ZIKV+HC-070-hoechst.jpg]

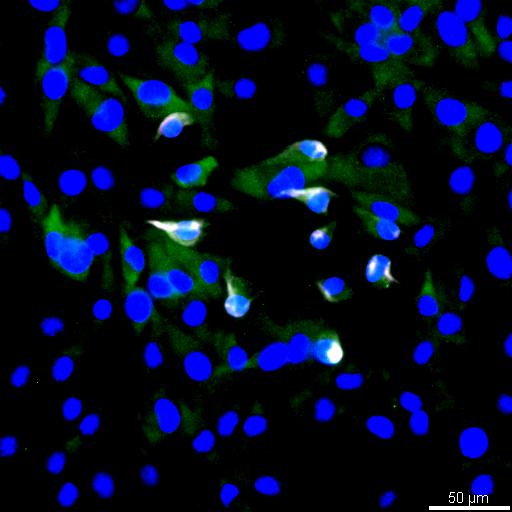

Supplement: Supplementary file 7 — Source data Fig. 5 [file 44321_2024_103_MOESM7_ESM.zip › Figure 5/5D/ZIKV+HC-070-merge.jpg]

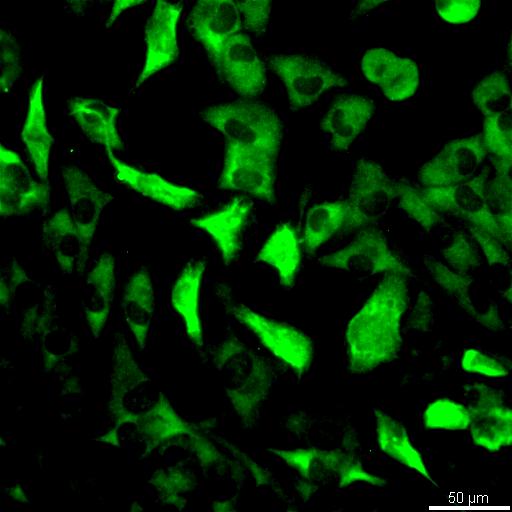

Supplement: Supplementary file 7 — Source data Fig. 5 [file 44321_2024_103_MOESM7_ESM.zip › Figure 5/5D/ZIKV-DDX3X.jpg]

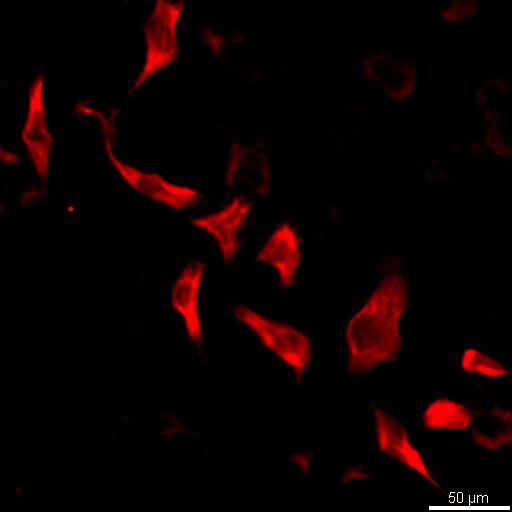

Supplement: Supplementary file 7 — Source data Fig. 5 [file 44321_2024_103_MOESM7_ESM.zip › Figure 5/5D/ZIKV-E protein.jpg]

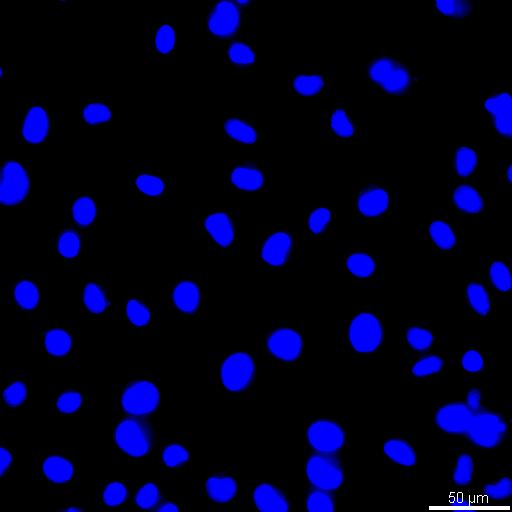

Supplement: Supplementary file 7 — Source data Fig. 5 [file 44321_2024_103_MOESM7_ESM.zip › Figure 5/5D/ZIKV-hochest.jpg]

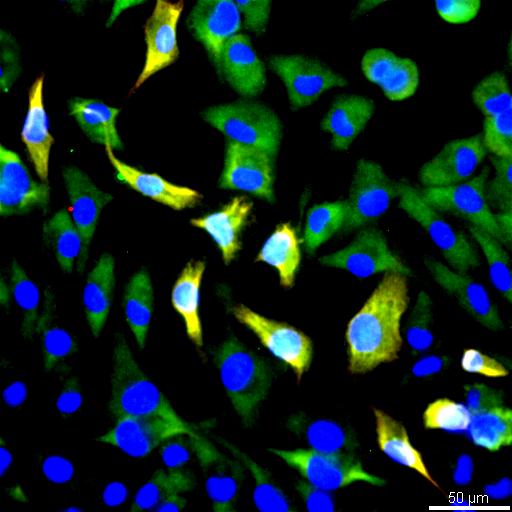

Supplement: Supplementary file 7 — Source data Fig. 5 [file 44321_2024_103_MOESM7_ESM.zip › Figure 5/5D/ZIKV-merge.jpg]

## Slide 1
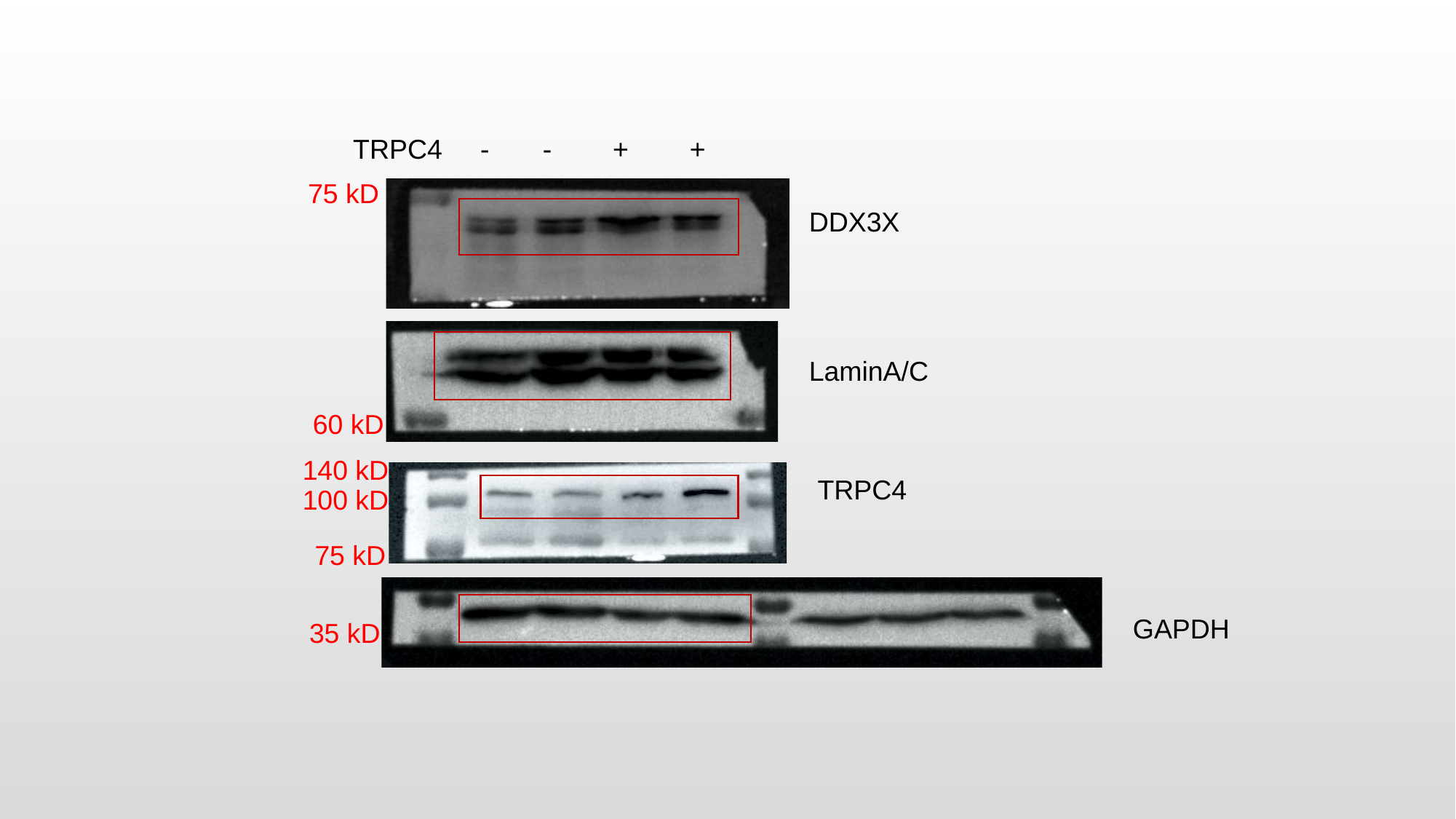

TRPC4 - - + +
75 kD
DDX3X
LaminA/C
60 kD
140 kD
TRPC4
100 kD
75 kD
GAPDH
35 kD

Supplement: Supplementary file 7 — Source data Fig. 5 [file 44321_2024_103_MOESM7_ESM.zip › Figure 5/5E/WB bands.pptx]
