# Supplementary material for: In vitro and in vivo inhibition of the host TRPC4 channel attenuates Zika virus infection
Source: EMBO Mol Med. 2024 Jul 15;16(8):3. doi: 10.1038/s44321-024-00103-4 (PMC11319825; doi:10.1038/s44321-024-00103-4)
Supplement: Supplementary file 6 — Source data Fig. 4 [file 44321_2024_103_MOESM6_ESM.zip › Figure 4/4C/WB bands.pptx]

## Slide 1
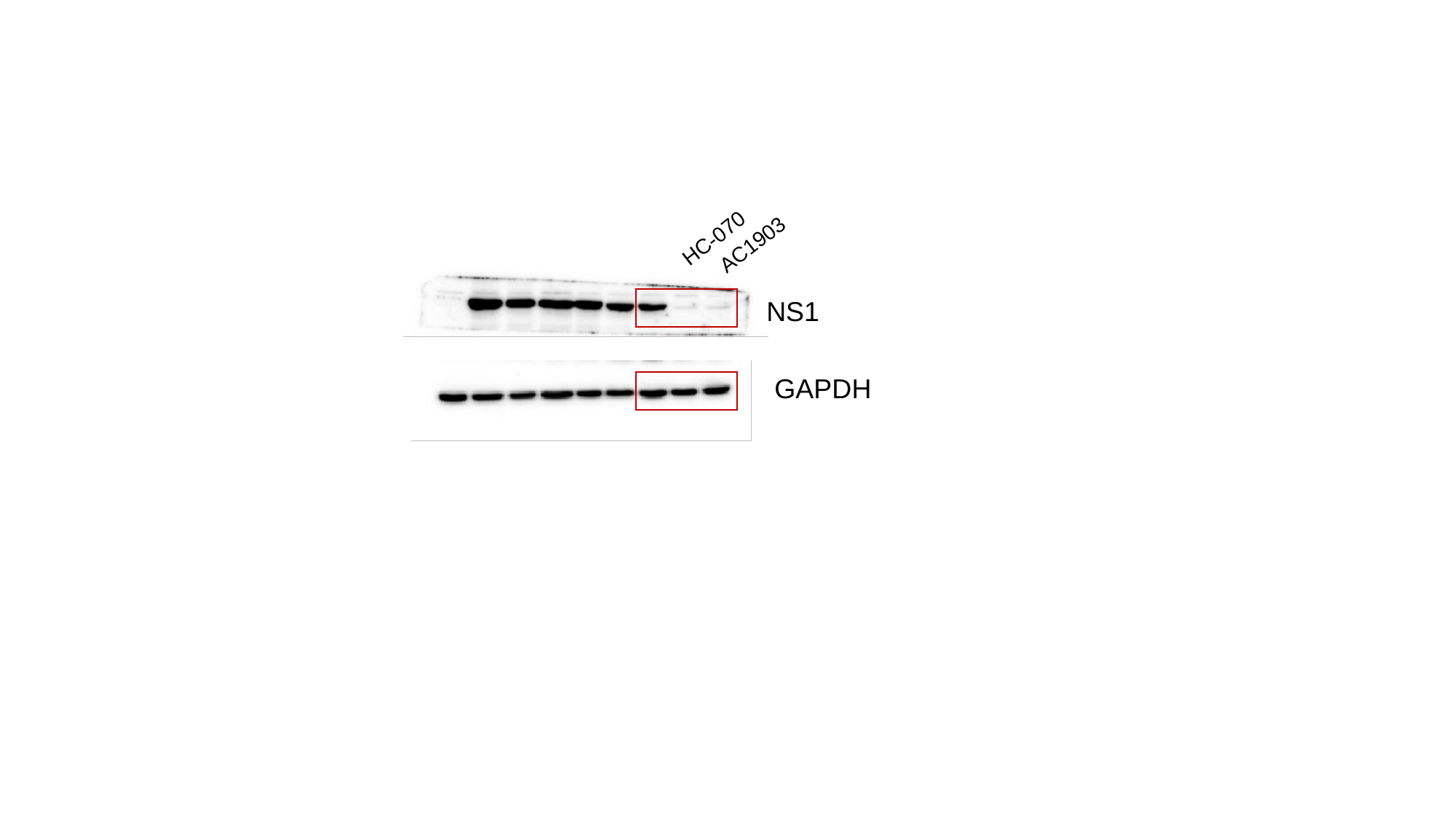

HC-070
AC1903
NS1
GAPDH
Figure 4D, The lines shown in the figure 4D are indicated with red boxes
Anti-GAPDH
Anti-NS1
