# Supplementary material for: In vitro and in vivo inhibition of the host TRPC4 channel attenuates Zika virus infection
Source: EMBO Mol Med. 2024 Jul 15;16(8):3. doi: 10.1038/s44321-024-00103-4 (PMC11319825; doi:10.1038/s44321-024-00103-4)
Supplement: Supplementary file 10 — Expanded View Figures [file 44321_2024_103_MOESM10_ESM.pdf]

Expanded View Figures

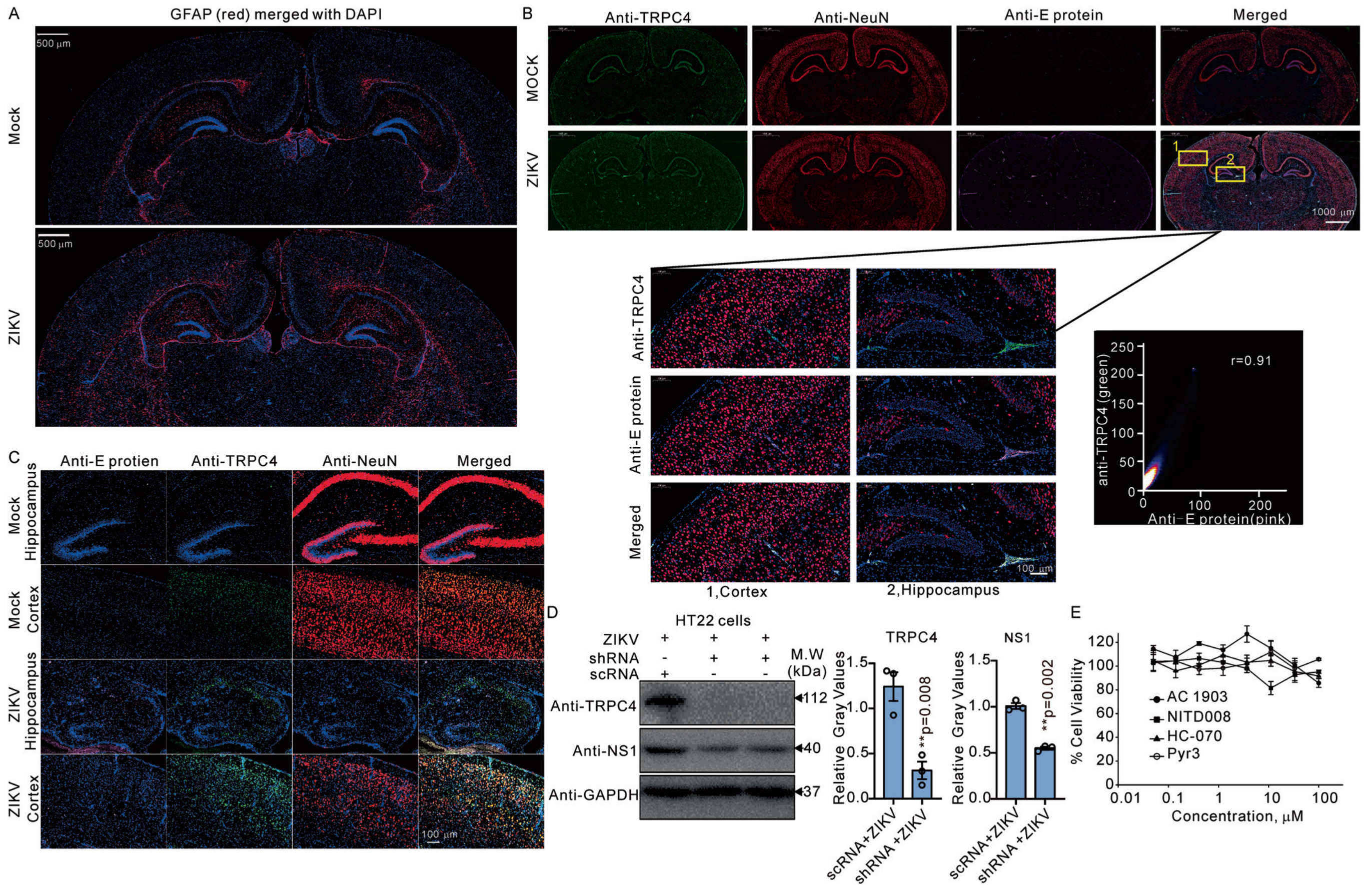

Figure EV1. The effects of ZIKV infection on brain inflammation and TRPC4 protein levels in A129 mice.

(**A**) ZIKV infection results in brain inflammation in A129 mice. Representative images of brain section immunolabeled for GFAP (red) and DAPI (blue). The scale bar represents 500 µm. (**B**) Representative images of the hippocampus and cortex from mock or ZIKV-infected A129 adult mice at 12 dpi immunolabeled for TRPC4 (green) and viral E protein (red). The scale bars represent 1000 µm and 100 µm, respectively. The right insert in (**B**) presents the Pearson correlation analysis, revealing an association between TRPC4 protein levels and viral E-protein levels ($r = 0.91$). (**C**) ZIKV infection also leads to an increase of the TRPC4 protein in neonatal mouse brain. The scale bar represents 100 µm. (**D**) the knockdown of TRPC4 by siRNA resulted in a reduction in NS1 production in HT22 cells ($n = 3$ biological replicates). (**E**) the mock-infected cell viability was assessed in the presence of either AC1903, NITD008, HC-070, or Pyr3 ($n = 3$ biological replicates). The unpaired *T* test (two-tailed) was employed to determine if there was a significant difference between two groups. Data information: In (**D**, **E**), data are presented as mean ± SEM, $^{**}P \le 0.001$.

© The Author(s)

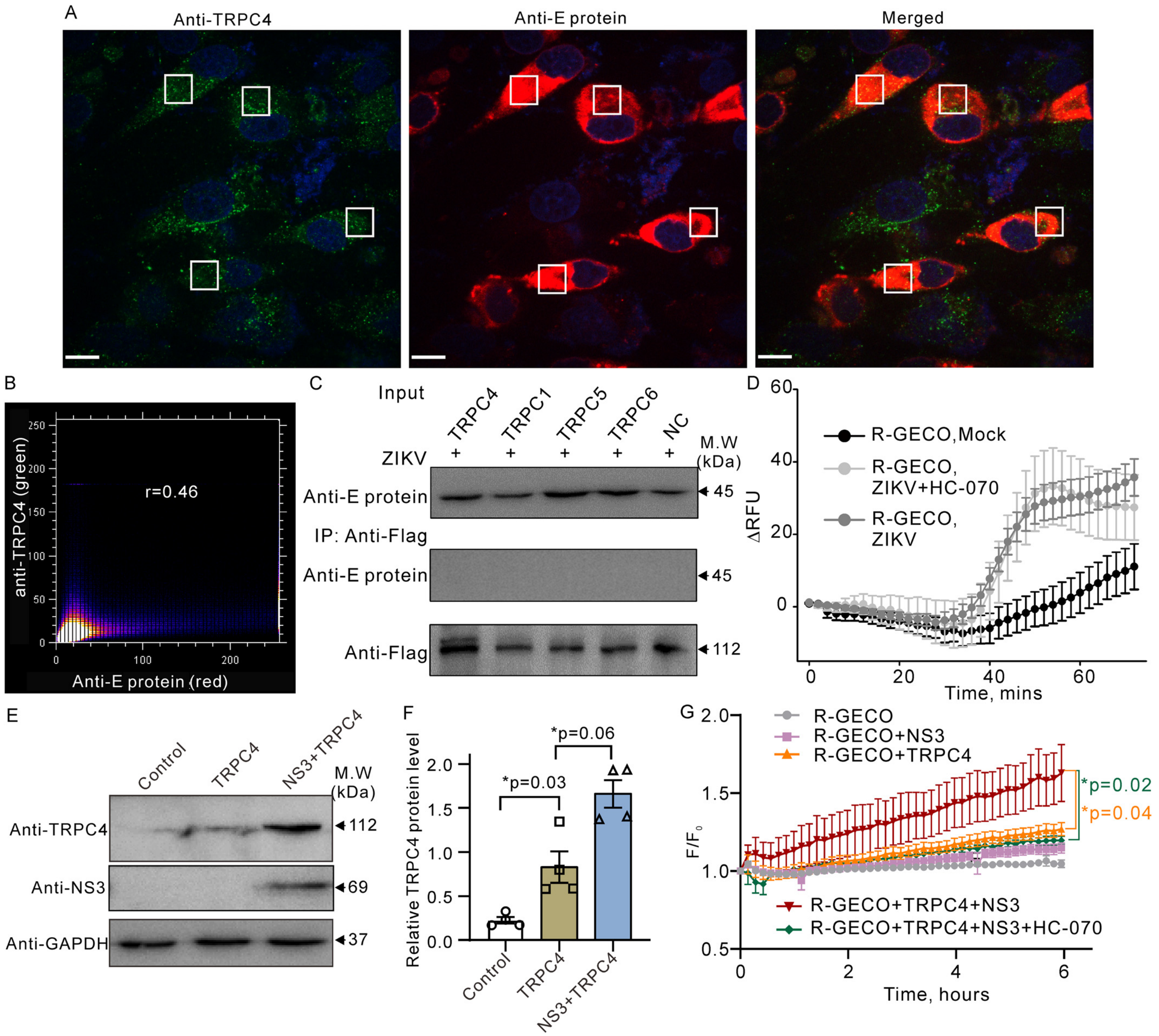

Figure EV2. The effect of ZIKV proteins on TRPC4 expression and function in BHK cells.

(**A**, **B**) BHK cells infected with ZIKV were co-stained with anti-TRPC4 (green) and anti-ZIKV-E-protein (red) antibodies. The scale bar represents 10 μm. The regions of interest (ROI), where correlation analyses are conducted, are depicted in the white boxes. Pearson's Coefficient ($r = 0.46$) was determined to assess the co-localization of the TRPC4 protein and the viral E protein. (**C**) the Co-IP of TRPCs protein and ZIKV-E protein in BHK cells. The anti-flag antibody was used to immunoprecipitate TRPC proteins tagged with flag from cell lysates. The immunoprecipitated complexes were subsequently analyzed using Western blots and probed using the anti-E-protein antibody. The data indicates that there was no association between TRPC and ZIKV-E proteins under our experimental conditions. (**D**) BHK cells were transfected with R-GECO Ca^{2+} sensor plasmid to monitor the intracellular Ca^{2+} levels in mock or ZIKV-infected cells ($n = 4$–5 biological replicates). ΔRFU represents the change in fluorescence intensity relative to the baseline. (**E**, **F**) A representative immunoblot and the results of densitometry analyses comparing relative TRPC4 protein levels in control, ZIKV-NS3, or ZIKV-NS3 + TRPC4 expressing BHK cells. GAPDH was used as a loading control. Co-expression of ZIKV-NS3 and TRPC4 cDNAs in BHK cells augmented the expression rate of the TRPC4 protein. (**G**) Normalized fluorescence intensity changes (F/F_o) in R-GECO cells co-expressing NS3 and/or TRPC4 in the presence or absence of 10 μM HC-070 ($n = 9$–12; 3–4 wells per each experiment; 3 biological replicates). The MANOVA test with Bonferroni correction was employed to determine if there was a significant difference among multiple groups. Data information: In (**D**, **F**, **G**), data are presented as mean ± SEM.

© The Author(s)

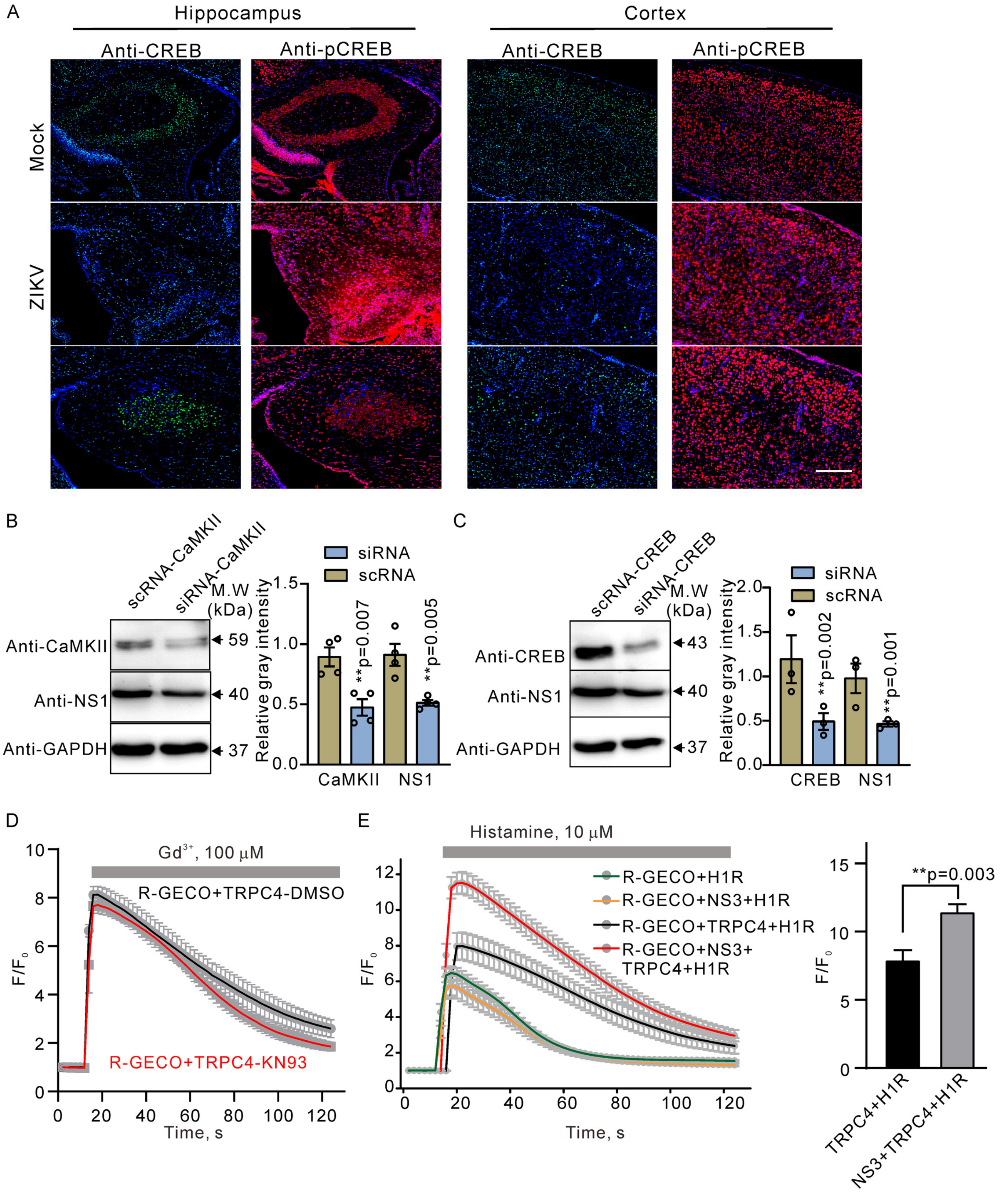
A
Hippocampus
Cortex
Anti-CREB
Anti-pCREB
Anti-CREB
Anti-pCREB
Mock
ZIKV
B
scRNA-CaMKII
siRNA-CaMKII
M.W (kDa)
Anti-CaMKII
Anti-NS1
Anti-GAPDH
59
40
37
siRNA
scRNA
Relative gray intensity
**p=0.007
**p=0.005
CaMKII
NS1
C
scRNA-CREB
siRNA-CREB
M.W (kDa)
Anti-CREB
Anti-NS1
Anti-GAPDH
43
40
37
siRNA
scRNA
Relative gray intensity
**p=0.002
**p=0.001
CREB
NS1
D
Gd3+, 100 μM
R-GECO+TRPC4-DMSO
R-GECO+TRPC4-KN93
F/F0
Time, s
E
Histamine, 10 μM
R-GECO+H1R
R-GECO+NS3+H1R
R-GECO+TRPC4+H1R
R-GECO+NS3+ TRPC4+H1R
F/F0
Time, s
**p=0.003
F/F0
TRPC4+H1R
NS3+TRPC4+H1R

◀ **Figure EV3. ZIKV-NS3 protein enhances TRPC4-mediated calcium signaling via the CaMKII-CREB pathway in neuronal cells.**

(**A**) ZIKV infection of the neonatal mouse brain elevates the protein level of pCREB. 1-day-old ICR mice were infected with ZIKV, and then the brains were collected at 12 dpi. Tissues were fixed and stained with anti-CREB (green), and anti-pCREB (red) antibodies. The scale bar represents 100 µm. (**B**, **C**) Representative Western blot images demonstrate the effectiveness of siRNA-induced reduction of ZIKV-NS1 protein production in HT22 cells ($n = 4$ biological replicates). Prior to exposing the cells to ZIKV (MOI 0.01), a 48-h transfection was performed using scRNAs or siRNAs that specifically target CaMKII ($n = 4$ biological replicates) or CREB ($n = 3$ biological replicates). (**D**) cells were transfected with R-GECO Ca^{2+} sensor plasmid to monitor the intracellular Ca^{2+} levels. No difference was observed between the Gd^{3+} (100 µM)-induced fluorescence increases in the presence of the vehicle (DMSO) or 10 µM KN-93 in R-GECO + TRPC4 expressing cells ($n = 6$ biological replicates). Gd^{3+} was applied at the times indicated with the horizontal bar. (**E**) The averaged normalized fluorescence increases induced by histamine (10 µM) in H1R (histamine receptor, green line), H1R + NS3 (orange line), TRPC4 + H1R (black line), or NS3 + TRPC4 + H1R (red line) expressing cells are shown (n = 6 biological replicates). Histamine was added at the times indicated by the horizontal bar. (**E**) Right panel, a comparison of averaged peak values for data shown in the left panel. The unpaired *T* test (two-tailed) was employed to determine if there was a significant difference between two groups. Data information: In (**B–E**), data are presented as mean ± SEM, $^{**}P \leq 0.001$.
